# Stepwise Assembly of Quinary Multivariate Metal–Organic Frameworks via Diversified Linker Exchange and Installation

**DOI:** 10.1021/jacs.3c03421

**Published:** 2023-06-15

**Authors:** Yuchen Hu, Xin Zhang, Rebecca Shu Hui Khoo, Christian Fiankor, Xu Zhang, Jian Zhang

**Affiliations:** † Department of Chemistry, University of Nebraska-Lincoln, Lincoln, Nebraska 68588, United States; ‡ Beijing Key Laboratory for Green Catalysis and Separation and Department of Chemical Engineering, Faculty of Environment and Life, Beijing University of Technology, Beijing 100124, China; § The Molecular Foundry, Lawrence Berkeley National Laboratory, Berkeley, California 94720, United States; ∥ School of Chemistry and Chemical Engineering, Huaiyin Normal University, Jiangsu Engineering Laboratory for Environment Functional Materials, Jiangsu Collaborative Innovation Center of Regional Modern Agriculture & Environmental Protection, No. 111, West Changjiang Road, Huaian, Jiangsu 223300, China

## Abstract

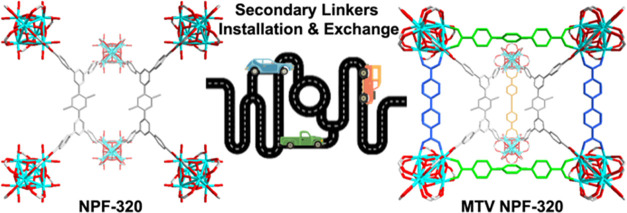

Multivariate MOFs (MTV-MOFs) constructed from multiple components with atomistic precision hold the promise for many fascinating developments in both fundamental sciences and applications. Sequential linker installation can be an effective method to introduce different functional linkers into an MOF that contains coordinatively unsaturated metal sites. However, in many cases, these linkers must be installed according to a specific sequence and the complete synthetic flexibility and freedom is yet to be realized. Here, we rationally decreased the size of the primary ligand used in NPF-300, a Zr-MOF with **scu** topology (NPF = Nebraska Porous Framework), and synthesized its isostructure, NPF-320. NPF-320 exhibits optimized pocket sizes which allow for the post-synthetic installation of three secondary linkers in all six permuted sequences via both linker exchange and installation, forming a final quinary MTV-MOF via single-crystal-to-single-crystal transformation. With the functionalization of the linkers from the quinary MOF system, one will be able to construct MTV-MOFs not only with variable porosity but also with unprecedented complexity and encoded synthetic sequence information. The utility of sequential linker installation was further demonstrated by the construction of a donor–acceptor pair-based energy transfer system.

## Introduction

Metal–organic frameworks (MOFs) are a class of crystalline solids constructed by the self-assembly of metal-containing nodes and organic linkers through coordination bonds and represent an emerging class of nanoporous solids with modular nature.^1−5^ The high level of structural and functional tunability of MOFs has led to many fascinating applications in gas storage, separation, chemical sensing, catalysis, energy harvesting, and biomedicine.^6−12^ Realizing advanced functionalities in MOFs typically requires more complex structures and pore environments.^13−16^ Therefore, constructing MOFs from multiple components, i.e., multivariate MOFs (MTV-MOFs),^17,18^ is one effective pathway to achieve highly complex structures with advanced properties, including emergent synergistic effects in cooperative catalysis and gas adsorption.^19−27^ One direct and facile approach to construct MTV-MOFs is to use organic linkers with similar length, geometry, and connectivity but different functional groups.^28,29^ However, the random distribution of the different functionality presents a challenge for structural characterization^29,30^ and thus the structure–property relationship. Although one-pot synthesis involving multiple linkers with different symmetry and connectivity has been reported, it is still deemed synthetically challenging to prepare complicated MTV-MOFs with atomic-level precision.^31−34^ This is particularly true for MTV-MOFs containing high-valent metals (i.e., Zr^4+^, Ti^4+^, Al^3+^, etc.) since the robust metal–ligand bonds in these systems typically limit the coordinative reversibility needed to form ordered crystalline structures.

On the other hand, post-synthetic linker installation appears to be a more reliable and effective method to precisely place multiple functional groups into predetermined positions with atomic-level precision.^35−41^ One prominent class of MOFs that can be subjected to linker installation is Zr-MOFs with low connectivity.^42^ The Zhou group first demonstrated the kinetically controlled installation of up to three different linear linkers into PCN-700 with **bcu** net, a coordinatively unsaturated Zr-MOF consisting of 8-connected Zr_6_O_4_(OH)_8_(H_2_O)_4_ SBUs.^43−46^ By replacing terminal OH^–^ and H_2_O ligands attached to Zr_6_-based clusters, up to three different linear linkers can be installed. A similar phenomenon has also been reported by Su and co-workers, where the same scaffold was utilized to place various functional groups containing linkers.^47−51^ We demonstrated the use of stepwise linker installation with up to three different kinds of extraneous linkers of different lengths in NPF-300 (NPF = Nebraska Porous Framework) with the **scu** topology, also consisting of 8-connected Zr_6_O_4_(OH)_8_(H_2_O)_4_ SBUs.^52^ A stepwise substitution of terminal ligands with three kinds of ditopic linkers yielded a quinary MOF with precisely placed functionalities.^52^ The flexibility of the tetratopic primary linker in NPF-300 ensures the formation of three crystallographically distinct pockets in the frameworks. Later, the Zhou group designed a trapezoidal to lower the crystal structure symmetry to create similar three distinct pockets in PCN-609,^53^ which can be used to accommodate three linear linkers of various lengths, creating unprecedented multivariate pore environments. One key finding in these two studies is that the size matching between the linkers and the vacancy sites is crucial for successful linker installation to form MTV-MOFs.

Despite the fascinating development, it is important to note that the three installation steps in either NPF-300 or PCN-609 must be performed in a specific sequence; otherwise only one or two linkers can be installed. Thus, the complete synthetic flexibility is yet to be realized. Herein, we report the stepwise assembly of quinary MTV-MOFs via both linker exchange and installation. Building on our previous work on NPF-300, we further decrease the size of the primary ligand and consequently the dimensions of the pockets in the resulting isostructural Zr-MOF named NPF-320. We demonstrate that the subtle size change of the parent framework affords a significant enhancement of synthesis freedom: through six different linker installation sequences three ditopic linkers with distinct lengths can thus be incorporated into the framework in a stepwise, single-crystal-to-single-crystal transformation fashion involving both linker exchange and linker installation processes. Furthermore, we demonstrate the utility of NPF-320 by sequential installation of an energy donor–acceptor pair and construction of an efficient energy transfer system for enhanced light harvesting. Our work paves the way for building multifunctional MOF materials for synergistic catalysis and gas storage applications, among others.^54,55^

## Results and Discussions

**Figure 1:**
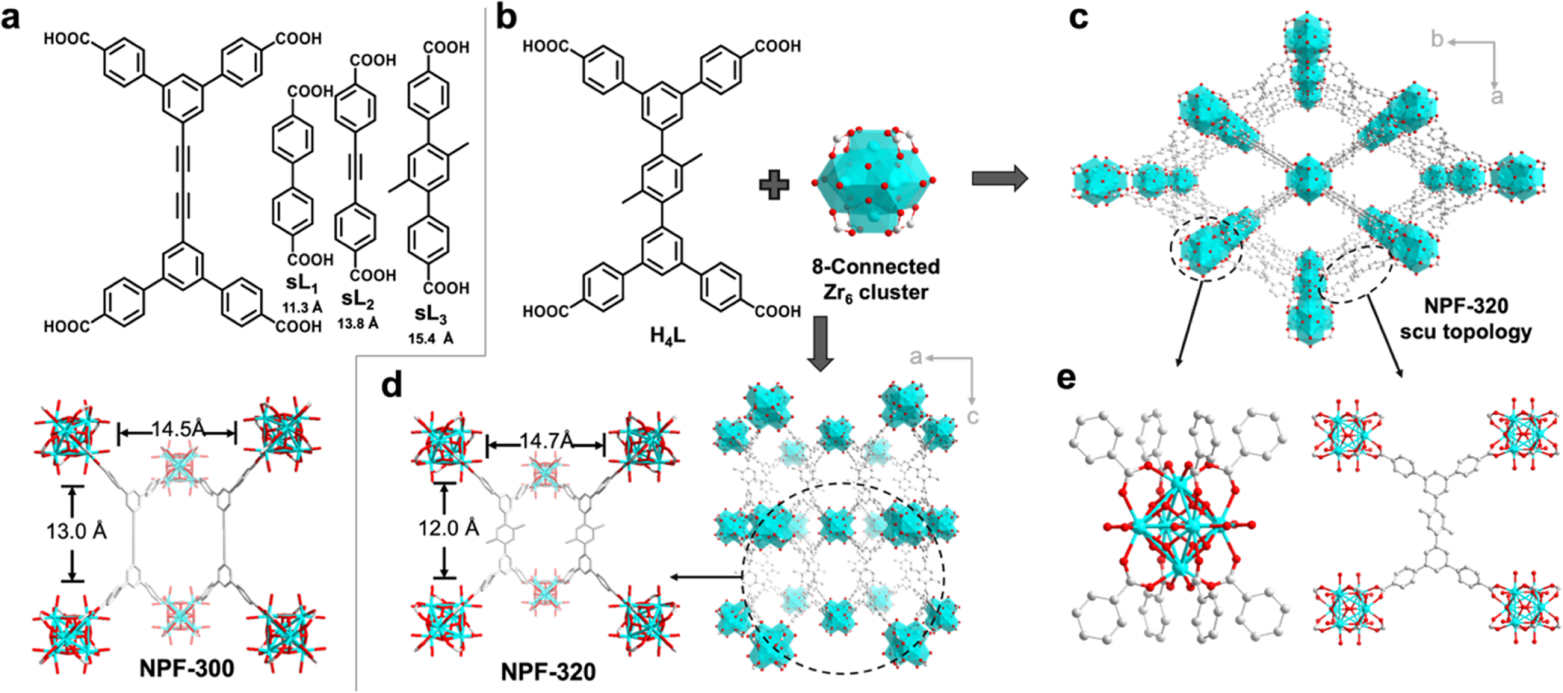
(a) Structures of the primary tetratopic ligand in NPF-300 and three secondary linkers, and a simplified structure of NPF-300 showing the pocket sizes along the *a* and *c* axes; (b) primary tetratopic ligand H_4_**L** and Zr_6_ cluster, their topological representation (C, gray; O, red; Zr, cyan polyhedron); (c) structure and topology of NPF-320; (d) simplified structures of NPF-320 showing the pocket sizes along the *a* and *c* axes; (e) connectivity of ligand **L** and Zr_6_ cluster.

**Figure 2:**
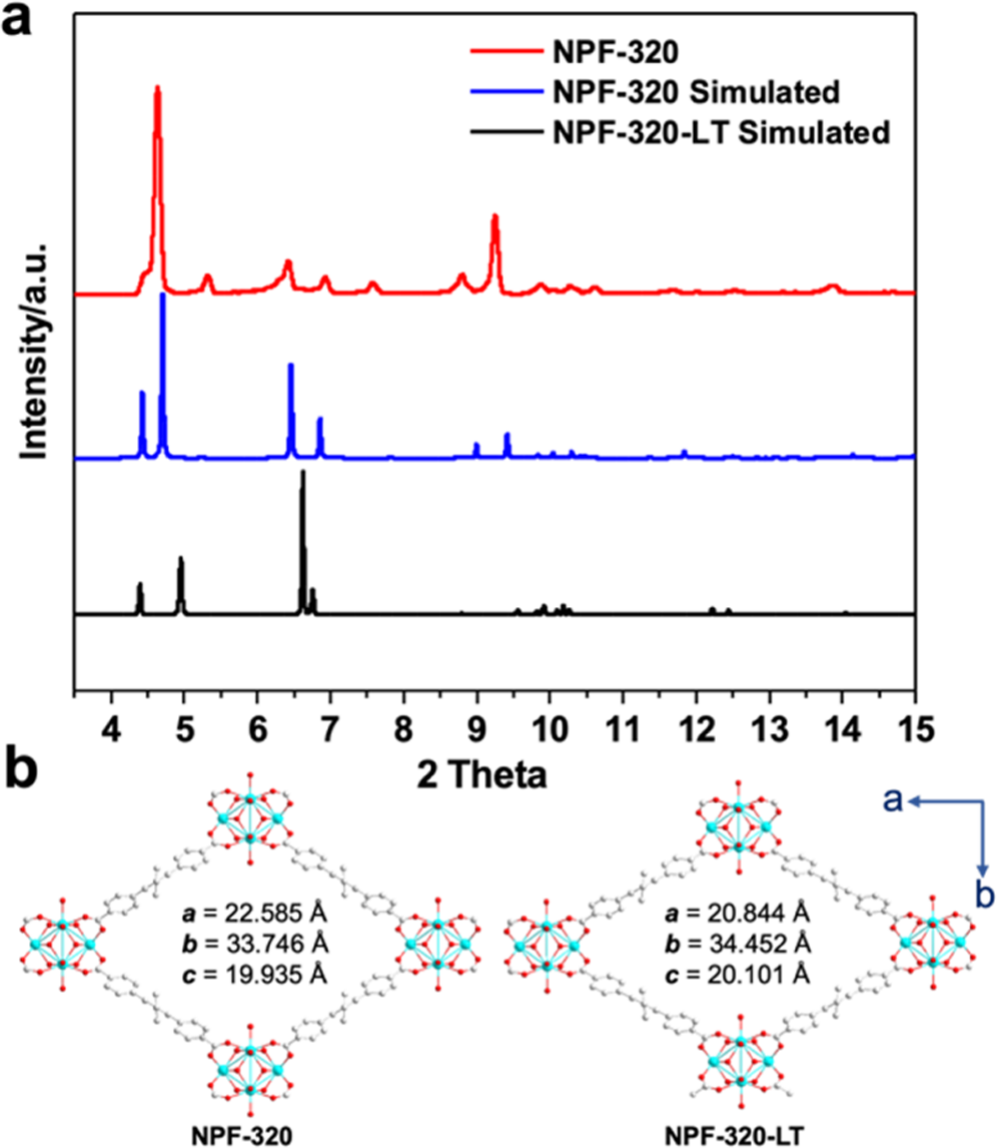
(a) Experimental and simulated powder XRD patterns of NPF-320 and NPF-320-LT. (b) Structural information and simplified structures of NPF-320 and NPF-320-LT are viewed from the *c* axis.

Our previous work has demonstrated that NPF-300 with the **scu** topology offers an ideal platform for linker installation in the empty pockets between the adjacent Zr_6_ clusters.^52^ Three secondary linkers **sL**_**1**_ (11.3 Å), **sL**_**2**_ (13.8 Å), and **sL**_**3**_ (15.4 Å) can be installed along the *a* and *c* axes based on the size-matching principle (Figure 1a). In this work, we decrease the size of the primary ligand used in NPF-300 by replacing the dialkyne group with a phenyl group and consequently the pocket size in the resulting Zr-MOF with the expectation to expand the flexibility of secondary linker installation. The tetratopic ligand H_4_**L** with reduced dimension (Figure 1b) was synthesized via the typical Suzuki couplings followed by saponification in basic aqueous solution (see Supporting Information for detailed procedures). Note that, the dimethyl groups were added in the central phenyl ring to increase the ligand solubility. A solvothermal reaction of ZrOCl_2_ with H_4_**L** in dimethylformamide (DMF) with benzoic acid as the modulating agent gave rise to light-yellow crystals of NPF-320. A single-crystal X-ray diffraction (sc-XRD) study at room temperature indicates that NPF-320 crystallizes in the *Cmmm* space group (No. 65; Table S1) of the orthorhombic system (Figure 1c). The powder X-ray diffraction (PXRD) patterns of the solvated NPF-320 exhibit an excellent agreement with the simulation, which confirms the bulk purity of the material (Figure 2a). As expected, NPF-320 is isostructural to NPF-300 with the same **scu** topology: each ligand is coordinated to four Zr_6_ clusters and each Zr_6_ cluster is connected to eight ligands with four above and four below the equatorial plane (Figure 1e). With the additional eight terminal H_2_O/OH^–^ groups in the equatorial plane, the overall formula of NPF-320 is Zr_6_O_4_(OH)_8_(H_2_O)_4_(**L**)_2_.

**Figure 3:**
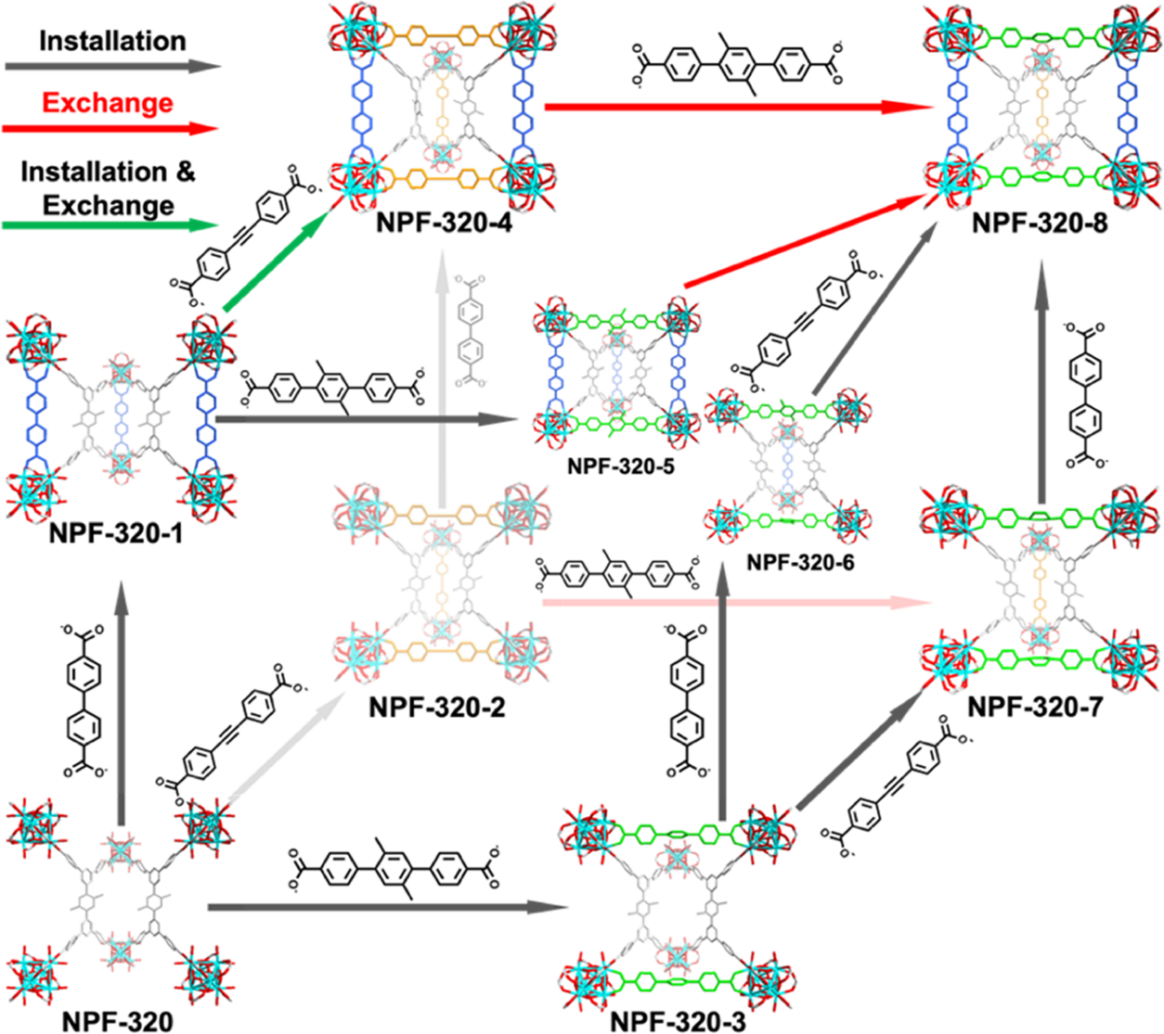
Stepwise installation of secondary linkers in NPF-320: gray arrows, linker installation only; red arrows, linker exchange only; green arrows, both linker installation and exchange.

Like NPF-300, NPF-320 also exhibits a temperature-dependent deformation, exemplified by the overall 5% decrease of unit cell volume based on the crystallographic data of NPF-320-LT measured at 100 K (Figure 2 and Table S1). This is resulted from a 7.7% decrease of the crystallographic *a* axis and a 2 and 0.8% increase of the *b* and *c* axes, respectively (Figure 2b). Noticeably, the decrease in the unit cell volume of NPF-320 is less than 7% of NPF-300, which might suggest a slight reduction of flexibility (*vide infra*). As we previously reported, the pocket size along the *c* axis in NPF-300, defined as the O–O distance between the opposing OH^–^/H_2_O groups on the Zr_6_ clusters, is 13.0 Å, which is longer than the length of **sL**_**1**_ (11.3 Å). In fact, this size mismatch fails to install **sL**_**1**_ as the first secondary linker in NPF-300.^52^ In NPF-320, however, this pocket size decreases to 12.0 Å, which is expected to fit more favorably to the dimension of **sL**_**1**_ (Figure 1). As such, we first carried out linker installation of **sL**_**1**_ in NPF-320 by incubating the parent MOF crystals in a DMF solution of **sL**_**1**_ at 60 °C for 24 h (Figure 3). Indeed, single-crystal-to-single-crystal transformation was realized, and the presence of the installed linker **sL**_**1**_ was unambiguously observed in the crystallographically resolved structure, termed as NPF-320-1, which crystallizes in the same *Cmmm* space group as NPF-320, with the full occupancy of **sL**_**1**_ at the expected pockets along the *c* axis. The overall composition of Zr_6_O_4_(OH)_6_(H_2_O)_2_(**L**)_2_(**sL**_**1**_) is confirmed by ^1^H NMR of a digested sample (*vide infra*, Table 1 and Figure S7).

**Table 1:** Linker Ratio of NPF-320 MTV-MOFs

installation route	1st linker	2nd linker	3rd linker	theoretical linker ratio	experimental linker ratio	product	
1	sL_1_			L:sL_1_ = 2:1	2.00:1.37	NPF-320-1	ternary
2	sL_2_			L:sL_2_ = 2:1.5	2.00:1.58	NPF-320-2	
3	sL_3_			L:sL_3_ = 2:1	2.00:1.20	NPF-320-3	
4	sL_2_	sL_1_		L:sL_2_:sL_1_ = 2:1.5:0.5	2.00:1.49:0.67	NPF-320-4	quaternary
5	sL_1_	sL_2_		L:sL_1_:sL_2_ = 2:0.5:1.5	2.00:0.54:1.51		
6	sL_1_	sL_3_		L:sL_1_:sL_3_ = 2:1:1	2.00:1.02:1.00	NPF-320-5	
7	sL_3_	sL_1_		L:sL_3_:sL_1_ = 2:1:0.5	2.00:1.02:1.15	NPF-320-6	
8	sL_3_	sL_2_		L:sL_3_:sL_2_ = 2:1:0.5	2.00:0.92:0.81	NPF-320-7	
9	sL_2_	sL_3_		L:sL_2_:sL_3_ = 2:0.5:1	2.00:0.54:0.98		
10	sL_3_	sL_2_	sL_1_	L:sL_3_:sL_2_:sL_1_ = 2:1:0.5:0.5	2.00:0.98:0.53:0.63	NPF-320-8	quinary
11	sL_2_	sL_3_	sL_1_	L:sL_2_:sL_3_:sL_1_ = 2:0.5:1:0.5	2.00:0.74:0.92:0.58		
12	sL_3_	sL_1_	sL_2_	L:sL_3_:sL_1_:sL_2_ = 2:1:0.5:0.5	2.00:1.00:0.69:0.55		
13	sL_1_	sL_3_	sL_2_	L:sL_1_:sL_3_:sL_2_ = 2:0.5:1:0.5	2.00:0.51:0.94:0.57		
14	sL_2_	sL_1_	sL_3_	L:sL_2_:sL_1_:sL_3_ = 2:0.5:0.5:1	2.00:0.53:0.63:1.04		
15	sL_1_	sL_2_	sL_3_	L:sL_1_:sL_2_:sL_3_ = 2:0.5:0.5:1	2.00:0.49:0.69:1.26		

Next, we attempted to install **sL**_**2**_ in NPF-320-1 along the *a* axis (Figure 3), and the resulting crystalline material, named NPF-320-4, crystallized in the **Immm** space group (No. 71; Table S1). To our surprise, in addition to the installation of **sL**_**2**_ in the expected pocket along the *a* axis, half of **sL**_**1**_ linkers installed along the *c* axis were replaced by **sL**_**2**_ in an alternating fashion, which gives rise to the overall formula of Zr_6_O_4_(OH)_4_(**L**)_2_(**sL**_**1**_)_0.5_(**sL**_**2**_)_1.5_. This represents a very rare single-crystal-to-single-crystal transformation involving both linker exchange and installation. We further reversed the order of linker installation since it is known to be an important factor dictating the formation of a particular product.^43^ Indeed, when **sL**_**2**_ was used as the first secondary linker in the sequential installation process, it was interestingly incorporated in the pockets not only along the *a* axis but also along the *c* axis with half occupancy and alternating fashion, forming NPF-320-2 in the *Immm* space group with the overall formula of Zr_6_O_4_(OH)_5_(H_2_O)(**L**)_2_(**sL**_**2**_)_1.5_. As expected, a subsequent installation of **sL**_**1**_ results in the formation of NPF-320-4. Taking together, in this work, the order of secondary linker installation of **sL**_**1**_ and **sL**_**2**_ does not make a difference to prepare NPF-320-4.

The co-presence of linker exchange and installation during the single-crystal-to-single-crystal transformation from NPF-320-1 to NPF-320-4 strongly points to a greater synthesis freedom in our system, that is, the order of linker installation might be freely changed to obtain the same final product. Encouraged by this finding, we carried out a study where the order of the secondary linker installation is systematically altered. The findings are outlined as follows:

(1) Overall, the synthesis first generates three ternary MOFs (i.e., NPF-320-1, -2, -3). By linker installation and/or exchange, four quaternary MOFs (i.e., NPF-320-4, -5, -6, -7) can be obtained, which are further converted into the final quinary NPF-320-8. To the best of our knowledge, this is the first time that six permuted installation sequences for three secondary linkers can all successfully result in the same final quinary MTV-MOF.
(2) The longest linker **sL**_**3**_ occupies the pocket along the *a* direction, the shortest **sL**_**1**_ occupies the pocket along the *c* direction, and **sL**_**2**_ with the intermediate length can occupy pockets along both directions. This is largely dictated by the size matching between the linker length and the pocket dimension along two directions, which is the same as observed in NPF-300 series.
(3) The shorter linker can be exchanged by a longer linker. There are four reactions involving linker exchange among all 15 linker installation routes: (a) **sL**_**1**_ exchanged by **sL**_**2**_ (NPF-320-1 → NPF-320-4 and NPF-320-5 → NPF-320-8) and (b) **sL**_**2**_ exchanged by **sL**_**3**_ (NPF-320-4 → NPF-320-8 and NPF-320-2 → NPF-320-7). Such a phenomenon was first observed by Rosi and co-workers in the transformation from Bio-MOF-101 to Bio-MOF-103, which also underwent a similar process where the shorter linker is replaced by the longer one.^56^
(4) Among the six routes to construct NPF-320-8 (Table 1, routes 10–15), only two do not involve linker exchange: route 10 (NPF-320 → NPF-320-3 → NPF-320-7 → NPF-320-8) and route 12 (NPF-320 → NPF-320-3 → NPF-320-6 → NPF-320-8), and both involve the installation of the longest **sL**_**3**_ along the *a* direction first, followed by the installation of the other two shorter linkers along the *c* direction, in which the order does not affect the final product.
(5) Although most of the transformations proceeded at a relatively low temperature of 60 °C, the linker exchange of **sL**_**2**_ by **sL**_**3**_ along the *a* direction requires a higher temperature of 80 °C. This is consistent with the better flexibility of the pocket along this direction and consequently a greater tolerance for linkers with different lengths.
(6) The formation of NPF-320-5 and NPF-320-6 indicates that in some cases the sequence of linker installation still plays a role (i.e., in the **sL**_**1**_/**sL**_**3**_ pair) and their difference lies in the full occupancy of **sL**_**1**_ in NPF-320-5 while the half occupancy in NPF-320-6. However, this does affect the formation of the final quinary MTV-MOF since both can result in NPF-320-8 via linker exchange from NPF-320-5 and linker installation from NPF-320-6.
(7) All eight MTV-MOFs were obtained as single crystals and characterized by sc-XRD. The PXRD patterns reveal the crystallinity of the bulk material and further confirm the nature of single-crystal-to-single-crystal transformation (Figures S22–S23). The composition of each framework product is determined by the ^1^H NMR spectrum of a base-digested sample (Table 1 and Figures S7–S21), which is consistent with the sc-XRD result. A few values deviated from the theoretical linker ratio. For example, the ratio of **L**:**sL**_**1**_ in NPF-320-1 is 2.00:1.37 instead of 2:1 (Table 1). A similar deviation occurs in NPF-320-6, of which the ratio of **L:sL**_**3**_**:sL**_**1**_ is 2.00:1.02:1.15 instead of 2:1:0.5. This can be explained by the random dangling of the secondary linker to the uncoordinated Zr_6_ cluster via one carboxylate.^34,50^ A similar deviation has also been observed in our previous study on NPF-300 series.^52^

The thermal stability of NPF-320 series was first evaluated by thermogravimetric analysis. All MOFs exhibit a high decomposition temperature of around 450–550 °C, indicating good thermal stability at elevated temperatures (Figure S24). Excellent crystallinity also remains after treatment in H_2_O, acidic (pH = 1), and basic (pH = 11) conditions (Figures S25–S26). However, the chemical stability of this series of MTV-MOFs is better quantified by digestion analysis since the partial or complete dissociation of secondary linkers does not necessarily result in a change of crystallinity. Indeed, the ^1^H NMR spectra of the base-digested samples revealed the dissociation of secondary linkers to various extents. Using the primary ligand **L** as the internal reference, the relative molar ratio before and after the treatments was determined as shown in Table 2 and Figures S27–S34.

**Table 2:** Chemical Stability of NPF-320 MTV-MOFs

	molar ratio	theoretical value	as-prepared	water	pH = 1	pH = 11
NPF-320-1	L: sL_1_	2: 1	2.00: 1.31	2.00: 1.30	2.00: 1.06	2.00: 0.99
NPF-320-2	L: sL_2_	2: 1.5	2.00: 1.51	2.00: 1.50	2.00: 0.25	2.00: 0.88
NPF-320-3	L: sL_3_	2: 1	2.00: 1.10	2.00: 1.00	2.00: 0.92	2.00: 0.98
NPF-320-4	L: sL_2_: sL_1_	2: 1.5: 0.5	2.00: 1.53: 0.55	2.00: 1.38: 0.47	2.00: 0.98: 0.32	2.00: 1.14: 0.36
NPF-320-5	L: sL_3_: sL_1_	2: 1: 1	2.00: 1.01: 1.02	2.00: 1.00: 1.00	2.00: 0.53: 0.94	2.00: 0.57: 1.00
NPF-320-6	L: sL_3_: sL_1_	2: 1: 0.5	2.00: 1.04: 1.12	2.00: 1.00: 0.88	2.00: 0.82: 0.51	2.00: 1.02: 0.69
NPF-320-7	L: sL_3_: sL_2_	2: 1: 0.5	2.00: 0.98: 0.71	2.00: 0.94: 0.66	2.00: 0.70: 0.32	2.00: 0.96: 0.49
NPF-320-8	L: sL_3_: sL_2_: sL_1_	2: 1: 0.5: 0.5	2.00: 0.92: 0.65: 0.65	2.00: 0.88: 0.61: 0.54	2.00: 0.80: 0.30: 0.28	2.00: 0.86: 0.52: 0.37

First, among the MTV-MOFs installed with one secondary linker, NPF-320-1 and NPF-320-3 exhibit excellent stability as indicated by the consistent **L**:**sL** molar ratio of ∼2:1 in water, basic, and acidic conditions (Table 2). This can be attributed to the good size matching of **sL**_**1**_ and **sL**_**3**_ with the distance of two open pockets along the *c* and *a* axes, respectively. However, NPF-320-2 shows poor stability and loses part of **sL**_**2**_, and the linker ratio of **L**:**sL**_**2**_ dropped from 2:1.51 to 2:0.25 after acid treatment and to 2:0.88 after base treatment. Second, among the MTV-MOFs installed with two secondary linkers, NPF-320-5 shows the highest stability, as demonstrated by the consistent **L**:**sL** molar ratio after water, acid, and base treatment. Like the dissociation of **sL**_**2**_ in NPF-320-2, **sL**_**2**_ was partially lost from NPF-320-4 and NPF-320-7. Furthermore, NPF-320-6 after base and acid treatment shows a close **L:sL** molar ratio to NPF-320-5, indicating that NPF-320-6 and NPF-320-5 can potentially convert to each other. In summary, the overall stability of MTV-MOFs follows the same trend as the NPF-300 series: water > pH = 11 > pH = 1. Except for NPF-320-6, all MTV-MOFs exhibit good stability in water and basic conditions. According to the results from all MTV-MOFs, the dissociation tendency follows the trend of **sL**_**2**_ > s**L**_**1**_ > s**L**_**3**_, which is related to the size matching with the open pockets.

We next determined the porosity parameters of NPF-320 series. Like NPF-300, NPF-320 does not retain the crystal structure and loses its crystallinity and permanent porosity (Figure S35). Indeed, after the activation by supercritical CO_2_ exchange,^57^ it exhibits only a low BET surface area (SA_BET_) of 280 m^2^/g (Figure S36), significantly lower than the calculated value of 3024 m^2^/g.^58^ However, the difference in NPF-320 series is that the installation of the secondary linker does not always facilitate retaining the permanent porosity. Specifically, only NPF-320-2 and NPF-320-6 exhibit an appreciable SA_BET_ of 2753 and 2632 m^2^/g (Figures S38 and S42), respectively, an increase of more than 9.8 and 9.4 times compared to NPF-320. The generally inferior retention of the permanent porosity in NPF-320 series compared to NPF-300 might be due to the more rigid primary ligand. The smaller temperature-induced unit cell volume change provides a reasonable support for this hypothesis. Indeed, the dialkyne linkage in the primary ligand in NPF-300 enables both in-plane and out-of-plane flexibilities (i.e., exemplified by a dihedral angle of 11.1° between the two central phenyl rings^52^) which help for the adaptation of the strain during the guest removal process. Such flexibility, however, is absent in NPF-320 series where a rigid phenyl group inhibits such intrinsic flexibility. It seems a certain combination of linker lengths along the *a* and *c* axes (i.e., either one short sL_1_ and one long sL_3_ as in NPF-320-6 or the medium-sized sL_2_ along both axes as in NPF-320-2) is required to achieve a balanced strain that results in the good stability and permanent porosity.

**Figure 4:**
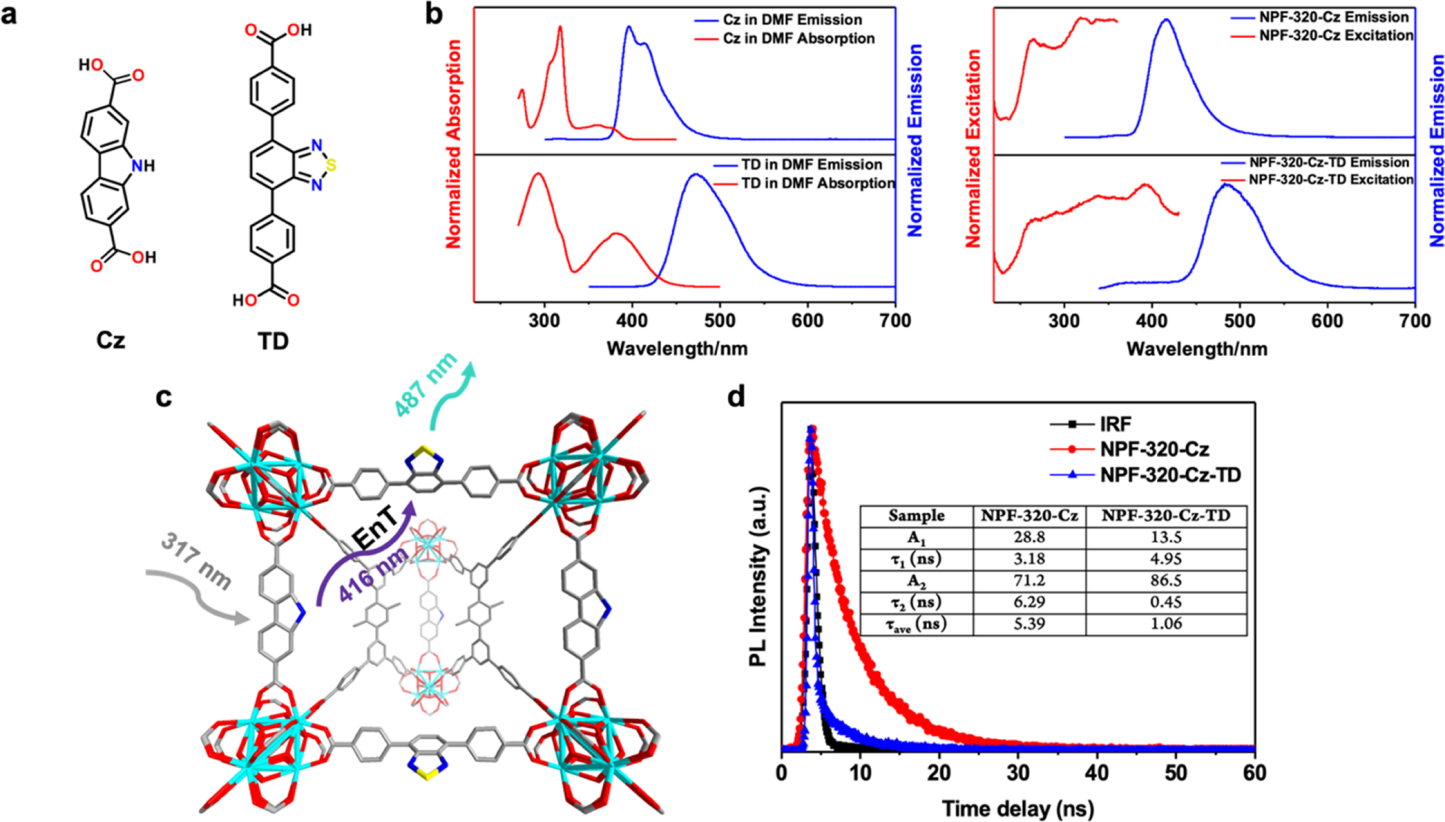
(a) Structures of Cz and TD installed in NPF-320-Cz-TD. (b) Left: absorption and emission spectra of Cz and TD in DMF; right: excitation and emission spectra of NPF-320-Cz and NPF-320-Cz-TD. (c) Structural model of NPF-320-Cz-TD showing the energy transfer from Cz to TD. (d) Emission lifetime decay at 416 nm of NPF-320-Cz-TD and NPF-320-Cz following 317 nm excitation.

Finally, we demonstrated the utility of the stepwise linker installation in NPF-320 in the construction of a donor–acceptor (D-A) energy transfer (EnT) system.^59^ Compared to the mixed linker approach,^60^ linker installation is a rational design strategy to access D-A EnT systems with well-defined molecular geometry. Recently, the Li group has used this strategy to construct D-A EnT systems, of which the main ligand and the installed secondary linker are the two EnT partners.^54^ Although a more recent work by the same group sequentially inserted two different linkers into PCN-700 to achieve the white light emission,^55^ to the best of our knowledge, such a strategy has not been used to build light-harvesting systems based on D-A EnT. Here, we chose a carbazole-based ditopic linker (Cz, matching the length of linker **sL**_**1**_) and a thiadiazole-based ditopic linker (TD, matching the length of linker **sL**_**3**_) as the energy donor and acceptor (Figure 4a), respectively. The ideal spectral overlap of the emission of Cz and the absorption of TD suggests that a favorable energy transfer is likely to occur (Figure 4b).^61^

Using the established sequential linker installation protocol, we were able to obtain NPF-320-Cz as the reaction intermediate and NPF-320-Cz-TD as the final quaternary MTV-MOF (see Supporting Information for a detailed synthetic procedure). PXRD patterns of NPF-320-Cz-TD and NPF-320-5 are comparable and also match well with those of NPF-320 (Figure S45) and the simulated PXRD patterns of its structural model, in which Cz and TD are installed in the pocket along the *c* and *a* axes, respectively (Figure 4c). Moreover, the ^1^H NMR spectrum of an acid-digested sample of NPF-320-Cz-TD (Figure S47) also indicates the success of the linker installation with the expected molar ratio.

Upon the excitation at 317 nm, the emission spectrum of NPF-320-Cz exhibits a broad band centered at ∼416 nm, which can be attributed to the emission of Cz (Figure 4b). To our delight, such emission is almost completely quenched upon the installation of the energy acceptor in NPF-320-Cz-TD, which only exhibits a broad emission band centered at ∼487 nm that is coming from the emission of TD (Figure 4b). Besides the significant D-A energy alignment described above, the close distance between Cz and TD in the framework (∼15 Å) is likely another important factor that contributes to the efficient EnT process. Fluorescence lifetime were measured to further confirm the presence of EnT. As shown in Figure 4d, compared to the emission lifetime of Cz in NPF-320-Cz measured at ∼416 nm, the emission lifetime of Cz in NPF-320-Cz-TD significantly decreases following the excitation at 317 nm, consistent with an efficient EnT process. The fluorescence decay kinetics of energy donor Cz in both NPF-320-Cz and NPF-320-Cz-TD gives the average emission lifetime of 5.39 and 1.06 ns (Figure 4d), respectively. We calculated the EnT time and efficiency using the following equations^62^
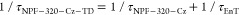
1
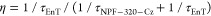
2where τ_NPF-320-Cz_ and τ_NPF-320-Cz-TD_ are the emission decay times for NPF-320-Cz and NPF-320-Cz-TD, respectively, and τ_EnT_ is the EnT time. The obtained EnT rate and efficiency are 1.32 ns^–1^ and 80%, respectively. Interestingly, this EnT efficiency is higher than that observed in NPF-500-H_2_TCPP (*η* = 69%),^63^ despite a similar D-A distance (∼14.8 Å in the latter), underlining the importance of orientation of the dipole moment of the donor and acceptor.^64^ Moreover, this EnT efficiency is comparable to previously reported efficient MOF-based EnT systems,^65^ suggesting the great potential of our mixed-ligand MOFs as efficient light-harvesting materials for photocatalytic applications.

## Conclusions

In conclusion, we have synthesized a new series of MTV-MOFs based on the (4,8)-connected NPF-320 using the post-synthetic sequential linker installation. The unique positions of eight-connected Zr_6_ and the flexibility of the primary ligand enable the precise insertion of up to three different secondary linkers along the *a* and *c* axes via stepwise, single-crystal-to-single-crystal transformation. We have revealed that the size matching of the installed linker and the order of installation are two important factors that govern the structures of MTV-MOFs. To the best of our knowledge, NPF-320 series display the most versatile insertion routes to introduce three distinct secondary linkers into a single MOF and produce up to eight MTV-MOFs with unprecedented complexity and encoded synthetic sequence information using post-synthetic stepwise linker installation. Our work also suggests that a certain degree of parent framework flexibility might be an important criterion to retain the permanent porosity. As a proof-of-concept study, we construct an efficient energy transfer system by sequential installation of two secondary linkers into NPF-320 that act as energy donors and acceptors, respectively. Overall, it is our expectation that NPF-320 can be used as a superior platform to build multifunctional MOF materials for a wide range of applications including synergistic/cooperative catalysis and gas storage/separation.

## Supporting Information Available

Synthesis, experimental data and procedures, X-ray diffraction, NMR spectra of digested MOFs, stability, and N_2_ adsorption isotherms (PDF)
