# Supplementary material for: Stepwise Assembly of Quinary Multivariate Metal–Organic Frameworks via Diversified Linker Exchange and Installation
Source: J Am Chem Soc. 2023 Jun 15;145(25):13929–37. doi: 10.1021/jacs.3c03421 (PMC10311524; doi:10.1021/jacs.3c03421)
Supplement: Supplementary file 1 — ja3c03421_si_001.pdf [file ja3c03421_si_001.pdf]

Supporting Information

Stepwise Assembly of Quinary Multivariate Metal−Organic Frameworks via Diversified Linker Exchange and Installation

Yuchen Hu,[1] Xin Zhang,[1,2] Rebecca Shu Hui Khoo,[3] Christian Fiankor,[1] Xu Zhang,[4*] and Jian Zhang[1,3*]

[1]Department of Chemistry, University of Nebraska-Lincoln, Lincoln, Nebraska 68588 United States

[2]Beijing Key Laboratory for Green Catalysis and Separation and Department of Chemical Engineering, Faculty of Environment and Life, Beijing University of Technology, Beijing 100124, China

[3]The Molecular Foundry, Lawrence Berkeley National Laboratory, Berkeley, California 94720 United States

[4]School of Chemistry and Chemical Engineering, Huaiyin Normal University, Jiangsu Engineering Laboratory for Environment Functional Materials, Jiangsu Collaborative Innovation Center of Regional Modern Agriculture & Environmental Protection, Huaian, Jiangsu 223300 China

S-1 Materials and General Procedures

All solvents and reagents were purchased from commercial suppliers and, unless otherwise noted, used without further purification. Solution ^{1}H and ^{13}C nuclear magnetic resonance NMR measurements were performed on a Bruker FT-NMR spectrometer (400 MHz) or a Bruker FT-NMR spectrometer (300 MHz). Mass spectra (MS) were performed on a Waters Q-TOF I mass spectrometer. Powder X-ray diffraction (PXRD) patterns were taken with a PANalytical Empyrean diffractometer with a PIXcel 3D detector. The copper target X-ray tube was set to 45 kV and 40 mA. Photoluminescence and excitation spectra were measured on a PerkinElmer LS55 spectrometer. UV-vis spectra were measured using an Agilent Cary 300 UV-vis spectrometer. Fluorescence lifetimes were measured on an Edinburgh FLS1000 spectrometer with an EPLED-365 light source. Gas adsorption isotherms were collected using the surface area analyzer ASAP-2020. N_2 gas adsorption isotherms were measured at 77 K using a liquid N_2 bath. The obtained adsorption-desorption isotherms were evaluated to give the pore parameters, including Brunauer-Emmett-Teller (BET) and Langmuir specific surface area, pore size, and pore volume.

Activation method of the MOF samples and for N_2 gas adsorption measurement: as-synthesized MOF samples were exchanged with fresh DMF at least three times. Then the MOFs samples with 1 mL DMF were added to 20 μL 8M HNO_3 and heated in an oven at 80 °C for 12 h to remove the unreacted ligand and cluster and modulators. The activated samples were exchanged with fresh DMF three times again and subsequently exchanged with anhydrous ethanol 3 times in 36 h to remove DMF completely. The ethanol exchanged samples were activated with a Samdri®-PVT-3D supercritical CO_2 dryer and immediately used for gas adsorption measurement.

Base digestion: MOF samples were dissolved in 500 μL DMSO-d_6 and 500 μL saturated K_3PO_4/D_2O solution by sonication for 5 mins and standing for 5 h. The DMSO-d_6 layer was used

for ^{1}H NMR measurement. Acid digestion was performed in 500 μL DMSO-d_6 with 25 μL D_2SO_4 at 60 °C until the solution get clear, then the solution was used for ^{1}H NMR measurement.

S-2 Synthesis of Primary Ligand, Secondary Linker and General Characterization

4,4'-(benzo[c][1,2,5]thiadiazole-4,7-diyl) dibenzoic acid (TD) was synthersized according to the procedure in the literature.[1]

Tetratopic ligand **H_4L** was synthesized via the typical Suzuki couplings followed by saponification in a basic aqueous solution. Below is the detailed synthesis of H_4L:

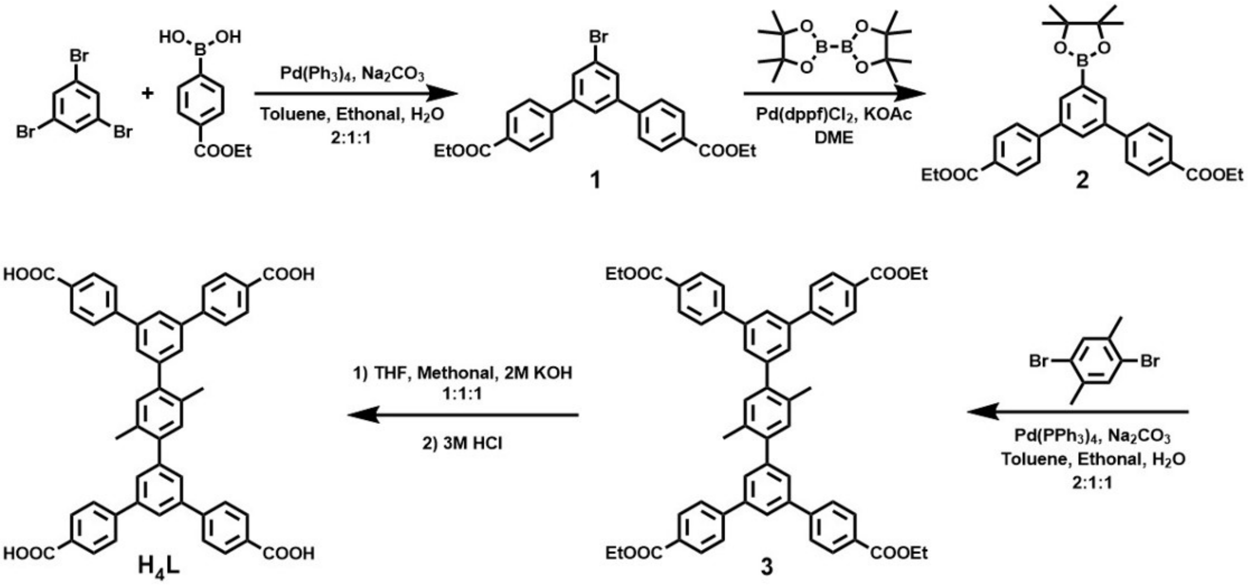

Scheme S1. Synthesis of Primary Ligand H_4L.

1,3-Bis(4-ethoxycarbonylphenyl)-5-bromobenzene (1): 1,3,5-Tribrombenzene (3.2 g, 10 mmol), *p*-ethoxyl-carbonphenylboronic acid (4.5 g, 25 mmol), Na_2CO_3 (8.5 g, 80 mmol) were dissolved in mixed solvent of toluene-ethanol-water (40 mL: 20 mL: 20 mL). After degassing by argon for 1 h, $Pd(PPh_3)_4$ (0.8 g, 0.68 mmol) was added to the solution. The solution was stirred under argon atmosphere for 3 d under reflux at 100 °C. After filtration, the solvent was removed under reduced pressure, the resulting residue was purified using column chromatography of silica gel to obtain compound 1 of 1.60 g (34 %). ^{1}H NMR (300 MHz, Chloroform-*d*) δ 8.21 – 8.13 (m, 4H), 7.80 (d, *J* = 1.6 Hz, 2H), 7.77 (t, *J* = 1.6 Hz, 1H), 7.74 – 7.67 (m, 4H), 4.44 (q, *J* = 7.1 Hz, 4H), 1.45 (t, *J* = 7.1 Hz, 6H).

3,5-bis(4-ethoxycarbonylphenyl)benzene boronic acid pinacol ester (2): Compound 1 (4.72 g, 9.44 mmol), bis(pinacolato)diboron (2.88 g, 11.3 mmol), and anhydrous potassium acetate (2.78 g, 28.3 mmol) were dissolved in dry dimethyl ether (45 mL). After degassing by argon for 1 hour, [1,1′ bis (diphenylphosphino) ferrocene] dichloro palladium (II) (0.345 g, 0.47 mmol)was added to the solution and heated at 90 °C for 30 h with vigorous stirring. It was cooled to room temperature and the mixture was diluted with 200 mL ethyl acetate and poured onto 100 mL DI water. The phases were separated, organics were washed with brine (3 x 100 mL) and dried with $MgSO_4$. After filtration, the residue was purified by using column chromatography of silica gel to obtain compound 2 of 4.49 g (95%). ^{1}H NMR (300 MHz, Chloroform-*d*) δ 8.19 – 8.12 (m, 4H), 8.11 (d, J = 1.9 Hz, 2H), 7.96 (t, J = 1.9 Hz, 1H), 7.81 – 7.74 (m, 4H), 4.44 (q, J = 7.1 Hz, 4H), 1.49 – 1.39 (m, 18H).

Diethyl 5',5'''-bis(4-(ethoxycarbonyl)phenyl)-2'',5''-dimethyl [1,1':3',1'':4''1''':3''', 1''''-quinquephenyl]-4,4''''-dicarboxylate (3): Compound 2 (1.62 g, 3.25 mmol), 1,4-dibromo-2,5-dimethylbenzene (0.39 g, 1.48 mmol), Na_2CO_3 (2.66 g, 25 mmol) were dissolved in mixed solvent of toluene-ethanol-water (20 mL: 10 mL: 10 mL). After degassing by argon for 1 hour, Pd $(PPh_3)_4$ (0.27 g, 0.23 mmol) was added to the solution. The solution was stirred under argon atmosphere for 3 d with reflux at 100 °C. After filtration, solvent was removed under reduced pressure, the residue was purified using column chromatography of silica gel to obtain compound 3 of 1.14 g (83%). ^{1}H NMR (400 MHz, Chloroform-*d*) δ 8.19 (d, J = 8.2 Hz, 8H), 7.88 (s, 2H), 7.79 (d, J = 8.2 Hz, 8H), 7.70 (s, 4H), 7.34 (s, 2H), 4.45 (q, J = 7.1 Hz, 8H), 2.42 (s, 6H), 1.46 (t, J = 7.1 Hz, 12H).

5',5'''-bis(4-carboxyphenyl)-2'',5''-dimethyl-[1,1':3',1'':4'',1''':3''',1'''' quinquephenyl] -4,4''''-dicarboxylic acid (H_4L): Compound 3 (1.14 g, 1.34 mmol) was

dissolved in a mixture of methanol (60 mL), THF (60 mL) and 2 M KOH (60 mL). The solution was degassed for 1 hour, and then refluxed under argon for 5 d and the solution became clear. Solvent was removed under reduced pressure, and the remaining solid was dissolved in water. 3 M HCl was added to the solution until pH = 2.0. After filtration, precipitate was recrystallized from DMF/water to obtain light yellow solid of 0.89 g (90 %). ^{1}H NMR (400 MHz, DMSO-d_6) δ 12.71 (s, 4H), 8.09 – 8.06 (m, 10H), 8.01 (d, J = 8.5 Hz, 8H), 7.78 (d, J = 1.7 Hz, 4H), 7.42 (s, 2H), 2.39 (s, 6H). ^{13}C NMR (101 MHz, DMSO-d_6) δ 167.62, 144.43, 143.03, 140.49, 133.00, 132.40, 130.44, 130.33, 128.01, 127.91, 127.81.

ESI MS found [M]+ m/z 738.22444, calcd [M]+ m/z: 738.23.

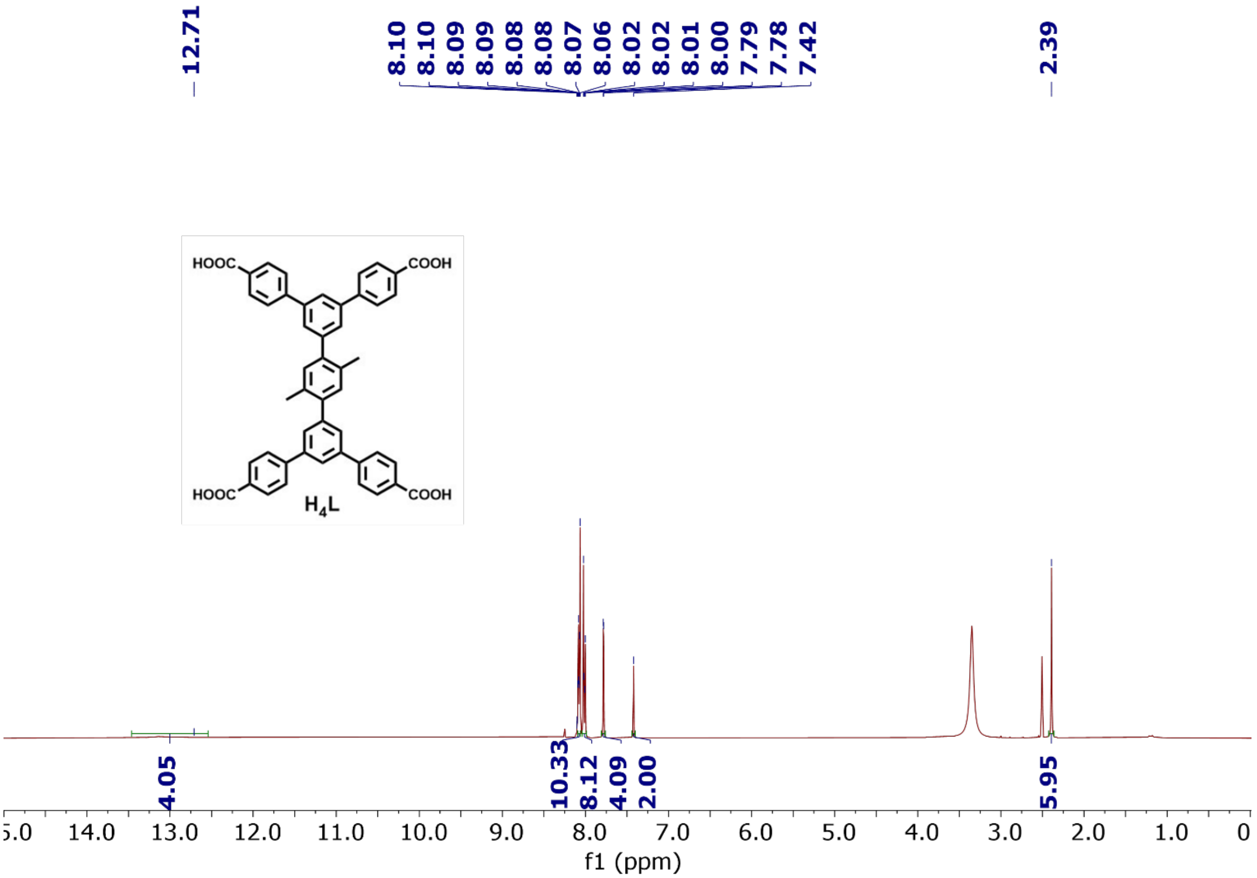

Figure S1. ^{1}H NMR spectrum of H_4L.

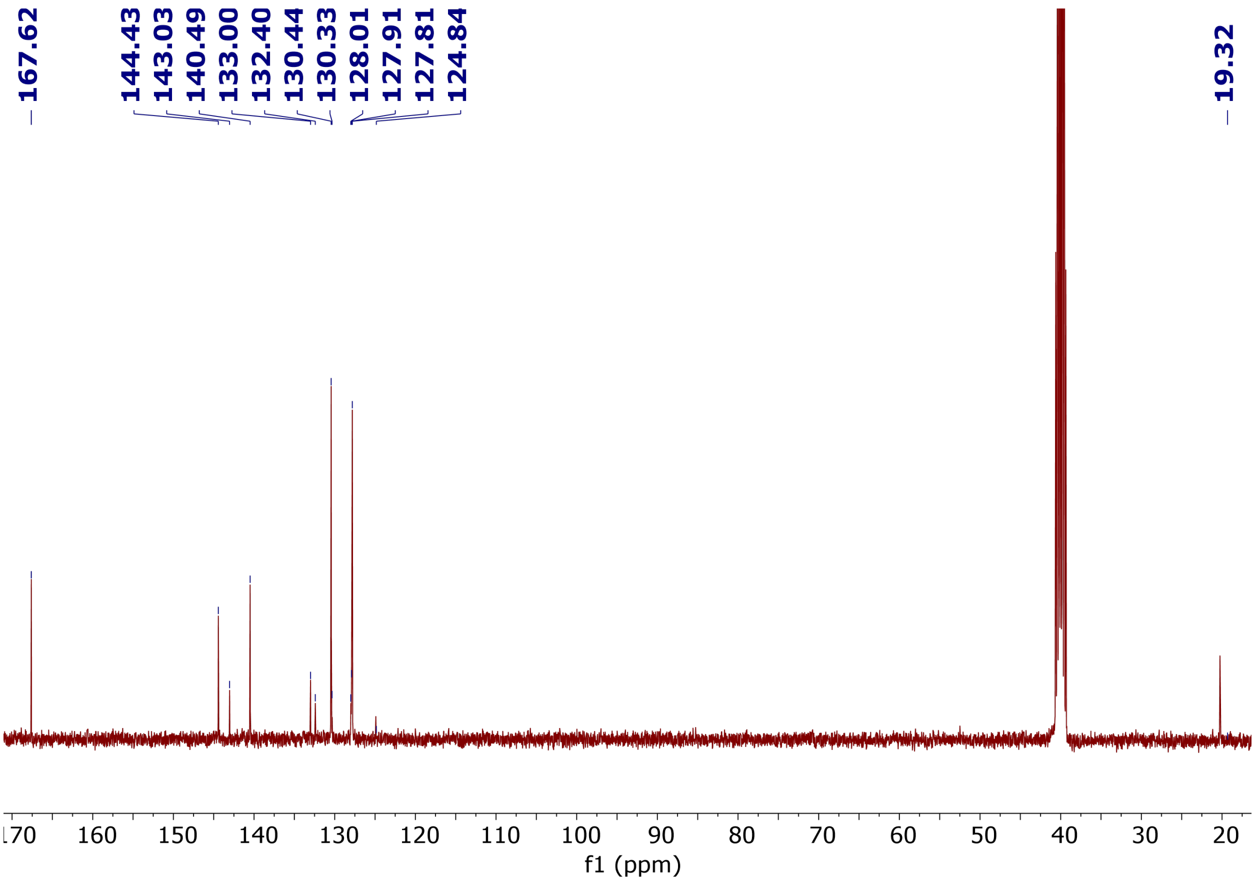

Figure S2. ^{13}C NMR spectrum of H_4L.

S-3 Synthesis of NPF-320 Series

Synthesis of NPF-320: 11 mg of $ZrOCl_2$ and 150 mg of benzoic acid were mixed in 1 mL DMF in a glass vial and ultrasonically dissolved. The clear solution was heated in an oven at 80 °C for 1 h. After cooling down to room temperature, 3 mg of ligand H_4L and 40 μL trifluoroacetic acid were added to this solution and the mixture was sonicated for 5 min to dissolve all ligands. Then the yellow solution was put into an oven and the temperature was increased from 30 °C to 120 °C in 2 h and then kept at 120 °C for 48 h. After cooling down to room temperature for 2 h, light yellow block-shaped single crystals were obtained. (Figure S3) As synthesized NPF-320 was activated by nitric acid to remove the unreacted ligand and cluster and modulators.

Synthesis of NPF-320-1:

Insertion Route 1: ~4 mg activated NPF-320 was soaked in 1 mL DMF solution of sL_1 (0.8 mg, 3.3 μmol). The sample was then put in an oven and the temperature was increased from 30 °C to 60 °C in 2 h and then kept at 60 °C for 12 h. After cooled down to room temperature, the solvent was exchanged with fresh DMF at least three times within 12 h.

Synthesis of NPF-320-2:

Insertion Route 2: ~4 mg activated NPF-320 was soaked in 1 mL DMF solution of sL_2 (0.9 mg, 3.4 μmol). The sample was then put in an oven and the temperature was increased from 30 °C to 60 °C in 2 h and then kept at 60 °C for 12 h. After cooled down to room temperature, the solvent was exchanged with fresh DMF at least three times within 12 h.

Synthesis of NPF-320-3:

Insertion Route 3: ~4 mg activated NPF-320 was soaked in 1 mL DMF solution of sL_3 (1 mg, 2.9 μmol). The sample was then put in an oven and the temperature was increased from 30 °C

to 60 °C in 2 h and then kept at 60 °C for 12 h. After cooled down to room temperature, the solvent was exchanged with fresh DMF at least three times within 12 h.

Synthesis of NPF-320-4:

Insertion Route 4: ~4 mg NPF-320-2 sample was soaked in 1 mL DMF solution of sL_1 (0.8 mg, 3.3 μmol). The sample was then put in an oven and the temperature was increased from 30 °C to 60 °C in 2 h and then kept at 60 °C for 12 h. After cooled down to room temperature, the solvent was exchanged with fresh DMF at least three times within 12 h.

Insertion Route 5: ~4 mg NPF-320-1 sample was soaked in 1 mL DMF solution of sL_2 (0.9 mg, 3.4 μmol). The sample was then put in an oven and the temperature was increased from 30 °C to 60 °C in 2 h and then kept at 60 °C for 12 h. After cooled down to room temperature, the solvent was exchanged with fresh DMF at least three times within 12 h.

Synthesis of NPF-320-5:

Insertion Route 6: ~4 mg NPF-320-1 sample was soaked in 1 mL DMF solution of sL_3 (1 mg, 2.9 μmol). The sample was then put in an oven and the temperature was increased from 30 °C to 60 °C in 2 h and then kept at 60 °C for 12 h. After cooled down to room temperature, the solvent was exchanged with fresh DMF at least three times within 12 h.

Synthesis of NPF-320-6:

Insertion Route 7: ~4 mg NPF-320-3 sample was soaked in 1 mL DMF solution of sL_1 (0.8 mg, 3.3 μmol). The sample was then put in an oven and the temperature was increased from 30 °C to 60 °C in 2 h and then kept at 60 °C for 12 h. After cooled down to room temperature, the solvent was exchanged with fresh DMF at least three times within 12 h.

Synthesis of NPF-320-7:

Insertion Route 8: ~4 mg NPF-320-3 sample was soaked in 1 mL DMF solution of sL_2 (0.9 mg, 3.4 µmol). The sample was then put in an oven and the temperature was increased from 30 °C to 60 °C in 2 h and then kept at 60 °C for 12 h. After cooled down to room temperature, the solvent was exchanged with fresh DMF at least three times within 12 h.

Insertion Route 9: ~4 mg NPF-320-2 sample was soaked in 1 mL DMF solution of sL_3 (1.0 mg, 2.9 µmol). The sample was then put in an oven and the temperature was increased from 30 °C to 80 °C in 2 h and then kept at 80 °C for 12 h. After cooled down to room temperature, the solvent was exchanged with fresh DMF at least three times within 12 h.

Synthesis of NPF-320-8:

Insertion Route 10: ~4 mg sample from Insertion Route 8 was soaked in 1 mL DMF solution of sL_1 (0.8 mg, 3.3 µmol). The sample was then put in an oven and the temperature was increased from 30 °C to 60 °C in 2 h and then kept at 60 °C for 12 h. After cooled down to room temperature, the solvent was exchanged with fresh DMF at least three times within 12 h.

Insertion Route 11: ~4 mg sample from Insertion Route 9 was soaked in 1 mL DMF solution of sL_1 (0.8 mg, 3.3 µmol). The sample was then put in an oven and the temperature was increased from 30 °C to 60 °C in 2 h and then kept at 60 °C for 12 h. After cooled down to room temperature, the solvent was exchanged with fresh DMF at least three times within 12 h.

Insertion Route 12: ~4 mg sample from Insertion Route 7 was soaked in 1 mL DMF solution of sL_2 (0.9 mg, 3.4 µmol). The sample was then put in an oven and the temperature was increased from 30 °C to 60 °C in 2 h and then kept at 60 °C for 12 h. After cooled down to room temperature, the solvent was exchanged with fresh DMF at least three times within 12 h.

Insertion Route 13: ~4 mg sample from Insertion Route 6 was soaked in 1 mL DMF solution of sL_2 (0.9 mg, 3.4 µmol). The sample was then put in an oven and the temperature was

increased from 30 °C to 60 °C in 2 h and then kept at 60 °C for 12 h. After cooled down to room temperature, the solvent was exchanged with fresh DMF at least three times within 12 h.

Insertion Route 14: ~4 mg sample from Insertion Route 4 was soaked in 1 mL DMF solution of sL_3 (1.0 mg, 2.9 μmol). The sample was then put in an oven and the temperature was increased from 30 °C to 80 °C in 2 h and then kept at 80 °C for 12 h. After cooled down to room temperature, the solvent was exchanged with fresh DMF at least three times within 12 h.

Insertion Route 15: ~4 mg sample from Insertion Route 5 was soaked in 1 mL DMF solution of sL_3 (1.0 mg, 2.9 μmol). The sample was then put in an oven and the temperature was increased from 30 °C to 80 °C in 2 h and then kept at 80 °C for 12 h. After cooled down to room temperature, the solvent was exchanged with fresh DMF at least three times within 12 h.

Synthesis of NPF-320-Cz (Insertion of Cz):

~8 mg (combined two vials) activated NPF-320 was soaked in 2 mL DMF solution of Cz (1.6 mg, 6.4 μmol). The sample was then put in an oven and the temperature was increased from 30 °C to 60 °C in 1 h and then kept at 60 °C for 12 h. After cooled down to room temperature, the solvent was exchanged with fresh DMF at least three times within 12 h.

Synthesis of NPF-320-Cz-TD (Insertion of Cz and TD):

~8 mg (combined two vials) of NPF-320-Cz was soaked in 4 mL DMF solution of TD (2.4 mg, 6.4 μmol). The sample was then put in an oven and the temperature was increased from 30 °C to 60 °C in 1 h and then kept at 60 °C for 12 h. After cooled down to room temperature, the solvent was exchanged with fresh DMF at least three times within 12 h.

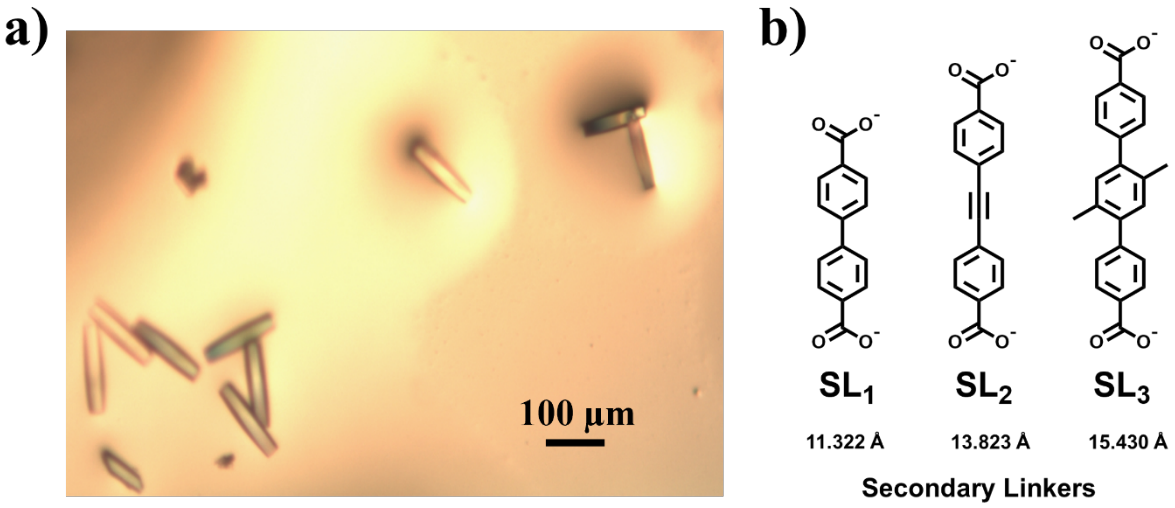

Figure S3. Crystals images of NPF-320 (a) and structures of secondary linkers (b).

S-4 Crystallographic Data and Structural Representation of NPF-320 Series

All samples were collected from the mother liquid, transferred to oil, then mounted onto glass fiber tips for low temperature (100 K) measurement. For room temperature data collection, crystals were sealed in a glass capillary with mother liquid. Single crystal X-ray diffraction data was collected using synchrotron radiation, either at the Advanced Photon Source, Argonne National Laboratory, Chicago, IL, or at the Advanced Light Source, Lawrence Berkeley National Laboratory. Indexing was performed using APEX2 (Difference Vectors method). Space groups were determined using XPREP implemented in APEX2. The structure was solved using SHELXS97 (direct methods) and refined using SHELXL-97 within Olex 2 (full-matrix least-squares on F2). Zr, C, O atoms were refined with anisotropic displacement parameters and H atoms were placed in geometrically calculated positions and included in the refinement process using the riding model with isotropic thermal parameters: Uiso(H) = 1.2Ueq(-CH). The contributions from disordered solvent molecules were treated as diffusion using the SQUEEZE method implemented in PLATON. Crystal data and refinement conditions are shown in the below tables.

Table S1. Crystal data and structure refinement

Compound name	NPF-320	NPF-320-LT	NPF-320-1
Empirical formula	$C_{96}H_{60}O_{32}Zr_6$	$C_{96}H_{60}O_{32}Zr_6$	$C_{110}H_{68}O_{32}Zr_6$
Formula weight	2272.76	2272.76	2448.96
Temperature/K	293(2)	100(2)	100(2)
Crystal system	orthorhombic	orthorhombic	orthorhombic
Space group	*Cmmm*	*Cmmm*	*Cmmm*
a/Å	22.585(3)	20.8437(9)	23.0073(12)
b/Å	33.746(5)	34.4518(14)	33.9923(17)
c/Å	19.935(3)	20.1009(8)	19.2577(9)
α/°	90	90	90
β/°	90	90	90
γ/°	90	90	90
Volume/Å^3	15194(4)	14434.5(10)	15060.9(13)
Z	2	2	2
ρ_{calc}g/cm^3	0.497	0.523	0.540
μ/mm^{-1}	0.224	0.236	0.228
F (000)	2264.0	2264.0	2447.9
Crystal size/mm^3	0.5×0.3×0.2 mm^3	0.5 × 0.3 × 0.2 mm^3	0.5 × 0.3 × 0.2 mm^3
Radiation	synchrotron (λ = 0.41328)	synchrotron (λ = 0.41328)	synchrotron (λ = 0.41328)
2Θ range for data collection/°	1.732 to 31.134	1.328 to 29.59	1.74 to 26.4
Reflections collected	202431	160635	137604
Independent reflections	9341[R_{int}=0.0938, R_{sigma}=0.0404]	7518[Rint=0.0837, Rsigma=0.0286]	5740[Rint=0.1366, Rsigma=0.0393]
Data/restraints/parameters	9341/0/168	7518/14/168	5740/70/210
Goodness-of-fit on F^2	1.094	1.055	1.091
Final R indexes [I>=2σ (I)]	R_1 = 0.0485 wR_2 = 0.1509	R1 = 0.0541 wR2 = 0.1765	R1 = 0.0395 wR2 = 0.1346
Final R indexes [all data]	R_1 = 0.0588 wR_2 = 0.1626	R1 = 0.0679 wR2 = 0.1988	R1 = 0.0514 wR2 = 0.1399
Largest diff. peak/hole / e Å^{-3}	1.45/-1.32	1.20/-1.50	1.25/-0.79

Compound name	NPF-320-2	NPF-320-3	NPF-320-4
Empirical formula	$C_{120}H_{72}O_{32}Zr_6$	$C_{116}H_{76}O_{32}Zr_6$	$C_{127}H_{76}O_{32}Zr_6$
Formula weight	2573.09	2529.08	2661.19
Temperature/K	100(2)	100(2)	100(2)
Crystal system	orthorhombic	orthorhombic	orthorhombic
Space group	*Immm*	*Cmmm*	*Immm*
a/Å	21.3117(9)	23.1553(14)	21.3949(11)
b/Å	33.7439(14)	33.130(2)	33.5956(16)
c/Å	40.3017(18)	19.9077(11)	40.5730(17)
α/°	90	90	90
β/°	90	90	90
γ/°	90	90	90
Volume/$Å^3$	28983(2)	15272.0(16)	29163(2)
Z	4	2	4
ρ_{calc}g/cm^3	0.590	0.550	0.606
μ/mm^{-1}	0.255	0.226	0.238
F (000)	5152.0	2536.0	5336.0
Crystal size/mm^3	0.5×0.3×0.2 mm^3	0.5 × 0.3 × 0.2 mm^3	0.5 × 0.3 × 0.2 mm^3
Radiation	synchrotron (λ = 0.7288)	synchrotron (λ = 0.41328)	synchrotron (λ = 0.41328)
2Θ range for data collection/°	3.346 to 54.192	1.724 to 30.116	2.194 to 29.96
Reflections collected	216348	171522	313067
Independent reflections	15727[R_{int}=0.0691,R_{sigma}=0.0311]	8093 [Rint = 0.1017, Rsigma = 0.0394]	15438[Rint=0.2466, Rsigma = 0.0763]
Data/restraints/parameters	15727/184/439	8093/92/221	15438/173/463
Goodness-of-fit on F^2	1.045	1.084	1.040
Final R indexes [I>=2σ (I)]	R1 = 0.0520 wR2 = 0.1710	R1 = 0.0505 wR2 = 0.1501	R1 = 0.0443 wR2 = 0.1179
Final R indexes [all data]	R1 = 0.0677 wR2 = 0.1840	R1 = 0.0640 wR2 = 0.1627	R1 = 0.0845 wR2 = 0.1376
Largest diff. peak/hole / e $Å^{-3}$	2.09/-1.57	2.27/-1.26	0.85/-0.94

Compound name	NPF-320-5	NPF-320-6	NPF-320-7
Empirical formula	$C_{132}H_{84}O_{32}Zr_6$	$C_{125}H_{80}O_{32}Zr_6$	$C_{126}H_{80}O_{32}Zr_6$
Formula weight	2729.31	2641.21	2653.22
Temperature/K	100(2)	100(2)	293(2)
Crystal system	orthorhombic	orthorhombic	orthorhombic
Space group	*Cmmm*	*Immm*	*Immm*
a/Å	23.5389(19)	23.1588(15)	23.140(2)
b/Å	33.406(3)	32.969(2)	32.609(3)
c/Å	19.6582(15)	39.854(2)	40.415(3)
α/°	90	90	90
β/°	90	90	90
γ/°	90	90	90
Volume/Å^3	15458(2)	30429(3)	30496(4)
Z	2	4	4
ρ_{calc}g/cm^3	0.586	0.577	0.578
μ/mm^{-1}	0.241	0.228	0.228
F (000)	2744.0	5304.0	5328.0
Crystal size/mm^3	0.5 × 0.3 × 0.2 mm^3	0.5 × 0.3 × 0.2 mm^3	0.5 × 0.3 × 0.2 mm^3
Radiation	synchrotron (λ = 0. 0.7288)	synchrotron (λ = 0.41328)	synchrotron (λ = 0.41328)
2Θ range for data collection/°	2.17 to 49.52	1.724 to 30.18	2.25 to 22.222
Reflections collected	117357	376147	160821
Independent reflections	6608 [Rint = 0.1423, Rsigma = 0.0582]	16686[R_{int}=0.1295,R_{sigma}=0.0470]	6943 [Rint = 0.2063, Rsigma = 0.0634]
Data/restraints/parameters	6608/186/262	16686/123/472	6943/217/420
Goodness-of-fit on F^2	1.109	1.025	1.063
Final R indexes [I>=2σ (I)]	R1 = 0.1044 wR2 = 0.2902	R1 = 0.0497 wR2 = 0.1426	R1 = 0.0583 wR2 = 0.1656
Final R indexes [all data]	R1 = 0.1240 wR2 = 0.3143	R1 = 0.0690 wR2 = 0.1597	R1 = 0.0838 wR2 = 0.1872
Largest diff. peak/hole / e Å^{-3}	3.37/-2.00	2.95/-0.99	1.36/-0.93

Compound name	NPF-320-8
Empirical formula	$C_{133}H_{84}O_{32}Zr_6$
Formula weight	2741.32
Temperature/K	100(2)
Crystal system	orthorhombic
Space group	*Immm*
a/Å	23.146(2)
b/Å	32.587(3)
c/Å	40.505(4)
α/°	90
β/°	90
γ/°	90
Volume/$Å^3$	30551(5)
Z	4
$\rho_{calc}g/cm^3$	0.596
μ/mm^{-1}	0.228
F (000)	5512.0
Crystal size/mm^3	0.5 × 0.3 × 0.2 mm^3
Radiation	synchrotron (λ = 0.41328)
2Θ range for data collection/°	1.866 to 22.62
Reflections collected	173249
Independent reflections	7230 [Rint = 0.2282, Rsigma = 0.0680]
Data/restraints/parameters	7230/187/499
Goodness-of-fit on F^2	1.094
Final R indexes [I>=2σ (I)]	R1 = 0.0447<br>wR2 = 0.1220
Final R indexes [all data]	R1 = 0.0774<br>wR2 = 0.1456
Largest diff. peak/hole / e $Å^{-3}$	1.41/-0.52

Table S2. The space groups and cell parameters of NPF-320-Cz-TD

Compound name	NPF-320-Cz-TD
Empirical formula	$C_{130}H_{77}N_3O_{40}SZr_6$
Formula weight	2900.43
Crystal system	orthorhombic
Space group	*Cmmm*
a/Å	23.155
b/Å	33.130
c/Å	19.908
α/°	90
β/°	90
γ/°	90
Volume/Å^3	15272.0
Z	2

Table S3. Atomic coordinates of NPF-320-Cz-TD

Zr	13.330506	16.565	11.703737
Zr	11.57765	14.106754	9.95385
O	11.57765	15.349129	11.717672
O	12.696051	16.565	13.88363
C	11.57765	16.565	14.664012
C	11.57765	16.565	16.176997
C	12.75857	16.565	16.993213
H	13.707938	16.565	16.49552
C	12.75857	16.565	18.446475
H	13.707938	16.565	18.844629
C	11.57765	16.565	19.163152
N	13.707938	16.565	19.9077
H	14.842547	16.565	19.9077
O	13.28188	17.764306	9.95385
O	9.069931	14.597078	7.477332
O	10.220749	13.013464	8.600126
O	15.451532	16.565	11.084607
C	16.014205	16.565	9.95385

C	17.496145	16.565	9.95385
C	18.174595	16.565	11.088589
H	17.730013	16.565	11.904805
C	19.573175	16.565	11.03882
H	20.029335	16.565	11.851054
C	20.286358	16.565	9.95385
C	21.761351	16.565	9.95385
C	22.449063	17.635099	9.95385
N	22.002166	18.95036	9.95385
H	22.002166	18.45341	9.95385
S	23.1553	20.10991	9.95385
C	9.338532	13.411024	7.771966
C	8.585985	12.344238	7.055289
C	8.653136	11.028977	7.495249
H	9.167183	10.807006	8.237806
C	7.949214	10.054955	6.818387
H	7.981632	9.180323	7.128947
C	7.201298	10.33656	5.705547
C	7.131832	11.678325	5.267577
H	6.627047	11.896983	4.517057
C	7.814914	12.662286	5.952402
H	7.757026	13.546857	5.669713
C	6.462644	9.286339	4.953036
C	5.788825	8.2825	5.66175
H	5.788825	8.2825	6.591439
C	6.448751	9.273087	3.599312
H	6.895648	9.945626	3.137454
C	5.788825	8.2825	2.8687
C	5.788825	8.2825	1.397521
C	4.624113	8.252683	0.668899
C	3.204694	8.236118	1.246222
H	3.202378	8.236118	2.205773
H	2.764743	9.024612	0.925708
H	2.739272	7.457563	0.925708
C	6.198674	7.199149	0.662926
C	6.633993	5.844132	1.230296
H	6.613154	5.900453	2.187856
H	6.07132	5.121898	0.939643
H	7.534735	5.681795	0.939643

H	3.762736	8.173171	1.122794
H	6.414018	6.354334	1.094923
O	10.459249	16.565	13.88363
C	10.39673	16.565	16.993213
H	9.447362	16.565	16.49552
C	10.39673	16.565	18.446475
H	9.447362	16.565	18.844629
N	9.447362	16.565	19.9077
H	8.312753	16.565	19.9077
O	12.696051	16.565	25.93177
C	11.57765	16.565	25.151388
C	11.57765	16.565	23.638403
C	12.75857	16.565	22.822187
H	13.707938	16.565	23.31988
C	12.75857	16.565	21.368925
H	13.707938	16.565	20.970771
C	11.57765	16.565	20.652248
O	10.459249	16.565	25.93177
C	10.39673	16.565	22.822187
H	9.447362	16.565	23.31988
C	10.39673	16.565	21.368925
H	9.447362	16.565	20.970771
O	9.069931	14.597078	-7.477332
O	10.220749	13.013464	-8.600126
C	9.338532	13.411024	-7.771966
C	8.585985	12.344238	-7.055289
C	8.653136	11.028977	-7.495249
H	9.167183	10.807006	-8.237806
C	7.949214	10.054955	-6.818387
H	7.981632	9.180323	-7.128947
C	7.201298	10.33656	-5.705547
C	7.131832	11.678325	-5.267577
H	6.627047	11.896983	-4.517057
C	7.814914	12.662286	-5.952402
H	7.757026	13.546857	-5.669713
C	6.462644	9.286339	-4.953036
C	5.788825	8.2825	-5.66175
H	5.788825	8.2825	-6.591439
C	6.448751	9.273087	-3.599312

H	6.895648	9.945626	-3.137454
C	5.788825	8.2825	-2.8687
C	5.788825	8.2825	-1.397521
C	4.624113	8.252683	-0.668899
C	3.204694	8.236118	-1.246222
H	3.202378	8.236118	-2.205773
H	2.764743	9.024612	-0.925708
H	2.739272	7.457563	-0.925708
C	6.198674	7.199149	-0.662926
C	6.633993	5.844132	-1.230296
H	6.613154	5.900453	-2.187856
H	6.07132	5.121898	-0.939643
H	7.534735	5.681795	-0.939643
H	3.762736	8.173171	-1.122794
H	6.414018	6.354334	-1.094923
O	2.507719	1.967922	7.477332
O	1.356901	3.551536	8.600126
C	2.239118	3.153976	7.771966
C	2.991665	4.220762	7.055289
C	2.924514	5.536023	7.495249
H	2.410467	5.757994	8.237806
C	3.628436	6.510045	6.818387
H	3.596018	7.384677	7.128947
C	4.376352	6.22844	5.705547
C	4.445818	4.886675	5.267577
H	4.950603	4.668017	4.517057
C	3.762736	3.902714	5.952402
H	3.820625	3.018143	5.669713
C	5.115006	7.278661	4.953036
C	5.128899	7.291913	3.599312
H	4.682002	6.619374	3.137454
C	6.953537	8.312317	0.668899
C	8.372956	8.328882	1.246222
H	8.375272	8.328882	2.205773
H	8.812907	7.540388	0.925708
H	8.838378	9.107437	0.925708
C	5.378976	9.365851	0.662926
C	4.943657	10.720868	1.230296
H	4.964496	10.664547	2.187856

H	5.50633	11.443102	0.939643
H	4.042915	10.883205	0.939643
H	7.814914	8.391829	1.122794
H	5.163632	10.210666	1.094923
O	2.507719	1.967922	-7.477332
O	1.356901	3.551536	-8.600126
C	2.239118	3.153976	-7.771966
C	2.991665	4.220762	-7.055289
C	2.924514	5.536023	-7.495249
H	2.410467	5.757994	-8.237806
C	3.628436	6.510045	-6.818387
H	3.596018	7.384677	-7.128947
C	4.376352	6.22844	-5.705547
C	4.445818	4.886675	-5.267577
H	4.950603	4.668017	-4.517057
C	3.762736	3.902714	-5.952402
H	3.820624	3.018143	-5.669713
C	5.115006	7.278661	-4.953036
C	5.128899	7.291913	-3.599312
H	4.682002	6.619374	-3.137454
C	6.953537	8.312317	-0.668899
C	8.372956	8.328882	-1.246222
H	8.375272	8.328882	-2.205773
H	8.812907	7.540388	-0.925708
H	8.838378	9.107437	-0.925708
C	5.378976	9.365851	-0.662926
C	4.943657	10.720868	-1.230296
H	4.964496	10.664547	-2.187856
H	5.50633	11.443102	-0.939643
H	4.042915	10.883205	-0.939643
H	7.814914	8.391829	-1.122794
H	5.163632	10.210666	-1.094923
O	15.451532	16.565	8.823093
C	18.174595	16.565	8.819111
H	17.730013	16.565	8.002895
C	19.573175	16.565	8.86888
H	20.029335	16.565	8.056646
C	22.449063	15.494901	9.95385
N	22.002166	14.17964	9.95385

H	22.002166	14.67659	9.95385
S	23.1553	13.02009	9.95385
O	30.859068	16.565	8.823093
C	30.296395	16.565	9.95385
C	28.814455	16.565	9.95385
C	28.136005	16.565	8.819111
H	28.580587	16.565	8.002895
C	26.737425	16.565	8.86888
H	26.281265	16.565	8.056646
C	26.024242	16.565	9.95385
C	24.549249	16.565	9.95385
C	23.861537	17.635099	9.95385
N	24.308434	18.95036	9.95385
H	24.308434	18.45341	9.95385
C	23.861537	15.494901	9.95385
N	24.308434	14.17964	9.95385
H	24.308434	14.67659	9.95385
O	30.859068	16.565	11.084607
C	28.136005	16.565	11.088589
H	28.580587	16.565	11.904805
C	26.737425	16.565	11.03882
H	26.281265	16.565	11.851054
Zr	9.824794	16.565	11.703737
Zr	11.57765	19.023246	9.95385
O	11.57765	17.780871	11.717672
O	9.87342	15.365694	9.95385
O	14.085369	18.532922	7.477332
O	12.934551	20.116536	8.600126
C	13.816768	19.718976	7.771966
C	14.569315	20.785762	7.055289
C	14.502164	22.101023	7.495249
H	13.988117	22.322994	8.237806
C	15.206086	23.075045	6.818387
H	15.173668	23.949677	7.128947
C	15.954002	22.79344	5.705547
C	16.023468	21.451675	5.267577
H	16.528253	21.233017	4.517057
C	15.340386	20.467714	5.952402
H	15.398275	19.583143	5.669713

C	16.692656	23.843661	4.953036
C	17.366475	24.8475	5.66175
H	17.366475	24.8475	6.591439
C	16.706549	23.856913	3.599312
H	16.259652	23.184374	3.137454
C	17.366475	24.8475	2.8687
C	17.366475	24.8475	1.397521
C	18.531187	24.877317	0.668899
C	19.950606	24.893882	1.246222
H	19.952922	24.893882	2.205773
H	20.390557	24.105388	0.925708
H	20.416028	25.672437	0.925708
C	16.956626	25.930851	0.662926
C	16.521307	27.285868	1.230296
H	16.542146	27.229547	2.187856
H	17.08398	28.008102	0.939643
H	15.620565	27.448205	0.939643
H	19.392564	24.956829	1.122794
H	16.741282	26.775666	1.094923
O	14.085369	18.532922	-7.477332
O	12.934551	20.116536	-8.600126
C	13.816768	19.718976	-7.771966
C	14.569315	20.785762	-7.055289
C	14.502164	22.101023	-7.495249
H	13.988117	22.322994	-8.237806
C	15.206086	23.075045	-6.818387
H	15.173668	23.949677	-7.128947
C	15.954002	22.79344	-5.705547
C	16.023468	21.451675	-5.267577
H	16.528253	21.233017	-4.517057
C	15.340386	20.467714	-5.952402
H	15.398275	19.583143	-5.669713
C	16.692656	23.843661	-4.953036
C	17.366475	24.8475	-5.66175
H	17.366475	24.8475	-6.591439
C	16.706549	23.856913	-3.599312
H	16.259652	23.184374	-3.137454
C	17.366475	24.8475	-2.8687
C	17.366475	24.8475	-1.397521

C	18.531187	24.877317	-0.668899
C	19.950606	24.893882	-1.246222
H	19.952922	24.893882	-2.205773
H	20.390557	24.105388	-0.925708
H	20.416028	25.672437	-0.925708
C	16.956626	25.930851	-0.662926
C	16.521307	27.285868	-1.230296
H	16.542146	27.229547	-2.187856
H	17.08398	28.008102	-0.939643
H	15.620565	27.448205	-0.939643
H	19.392564	24.956829	-1.122794
H	16.741282	26.775666	-1.094923
O	20.647581	31.162078	7.477332
O	21.798399	29.578464	8.600126
C	20.916182	29.976024	7.771966
C	20.163635	28.909238	7.055289
C	20.230786	27.593977	7.495249
H	20.744833	27.372006	8.237806
C	19.526864	26.619955	6.818387
H	19.559282	25.745323	7.128947
C	18.778948	26.90156	5.705547
C	18.709482	28.243325	5.267577
H	18.204697	28.461983	4.517057
C	19.392564	29.227286	5.952402
H	19.334676	30.111857	5.669713
C	18.040294	25.851339	4.953036
C	18.026401	25.838087	3.599312
H	18.473298	26.510626	3.137454
C	16.201763	24.817683	0.668899
C	14.782344	24.801118	1.246222
H	14.780028	24.801118	2.205773
H	14.342393	25.589612	0.925708
H	14.316922	24.022563	0.925708
C	17.776324	23.764149	0.662926
C	18.211643	22.409132	1.230296
H	18.190804	22.465453	2.187856
H	17.64897	21.686898	0.939643
H	19.112385	22.246795	0.939643
H	15.340386	24.738171	1.122794

H	17.991668	22.919334	1.094923
O	20.647581	31.162078	-7.477332
O	21.798399	29.578464	-8.600126
C	20.916182	29.976024	-7.771966
C	20.163635	28.909238	-7.055289
C	20.230786	27.593977	-7.495249
H	20.744833	27.372006	-8.237806
C	19.526864	26.619955	-6.818387
H	19.559282	25.745323	-7.128947
C	18.778948	26.90156	-5.705547
C	18.709482	28.243325	-5.267577
H	18.204697	28.461983	-4.517057
C	19.392564	29.227286	-5.952402
H	19.334676	30.111857	-5.669713
C	18.040294	25.851339	-4.953036
C	18.026401	25.838087	-3.599312
H	18.473298	26.510626	-3.137454
C	16.201763	24.817683	-0.668899
C	14.782344	24.801118	-1.246222
H	14.780028	24.801118	-2.205773
H	14.342393	25.589612	-0.925708
H	14.316922	24.022563	-0.925708
C	17.776324	23.764149	-0.662926
C	18.211643	22.409132	-1.230296
H	18.190804	22.465453	-2.187856
H	17.64897	21.686898	-0.939643
H	19.112385	22.246795	-0.939643
H	15.340386	24.738171	-1.122794
H	17.991668	22.919334	-1.094923
O	9.87342	17.764306	9.95385
O	13.28188	15.365694	9.95385
Zr	13.330506	16.565	8.203963
O	11.57765	17.780871	8.190028
Zr	9.824794	16.565	8.203963
O	11.57765	15.349129	8.190028
Zr	13.330506	16.565	-8.203963
Zr	9.824794	16.565	-8.203963
Zr	32.980094	16.565	11.703737
Zr	13.330506	16.565	28.111663

Zr	9.824794	16.565	28.111663
Zr	32.980094	16.565	8.203963
Zr	1.752856	0	-8.203963
Zr	21.402444	33.13	-8.203963
Zr	1.752856	0	8.203963
Zr	21.402444	33.13	8.203963
Zr	11.57765	14.106754	-9.95385
Zr	11.57765	19.023246	-9.95385
Zr	23.1553	30.671754	-9.95385
Zr	23.1553	30.671754	9.95385
Zr	0	2.458246	-9.95385
Zr	0	2.458246	9.95385
O	12.696051	16.565	6.02407
O	10.459249	16.565	6.02407
O	9.069931	18.532922	12.430368
O	14.085369	14.597078	12.430368
O	14.085369	18.532922	12.430368
O	9.069931	14.597078	12.430368
O	14.085369	14.597078	7.477332
O	9.069931	18.532922	7.477332
O	10.220749	20.116536	11.307574
O	12.934551	13.013464	11.307574
O	12.934551	20.116536	11.307574
O	10.220749	13.013464	11.307574
O	12.934551	13.013464	8.600126
O	10.220749	20.116536	8.600126
O	7.703768	16.565	11.084607
O	7.703768	16.565	8.823093

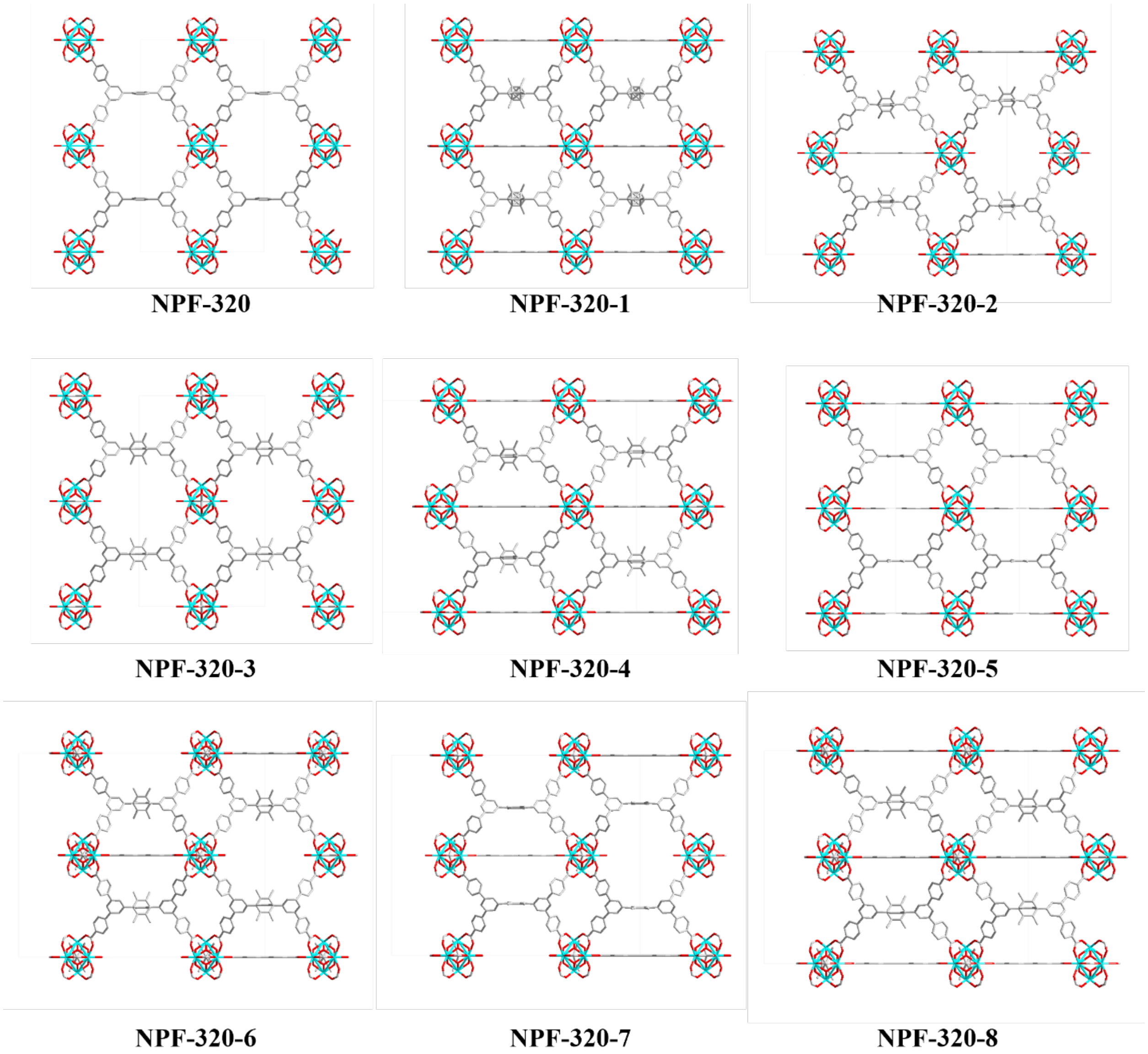

Figure S4. Crystal structures of NPF-320 Series viewed along the *a* axis.

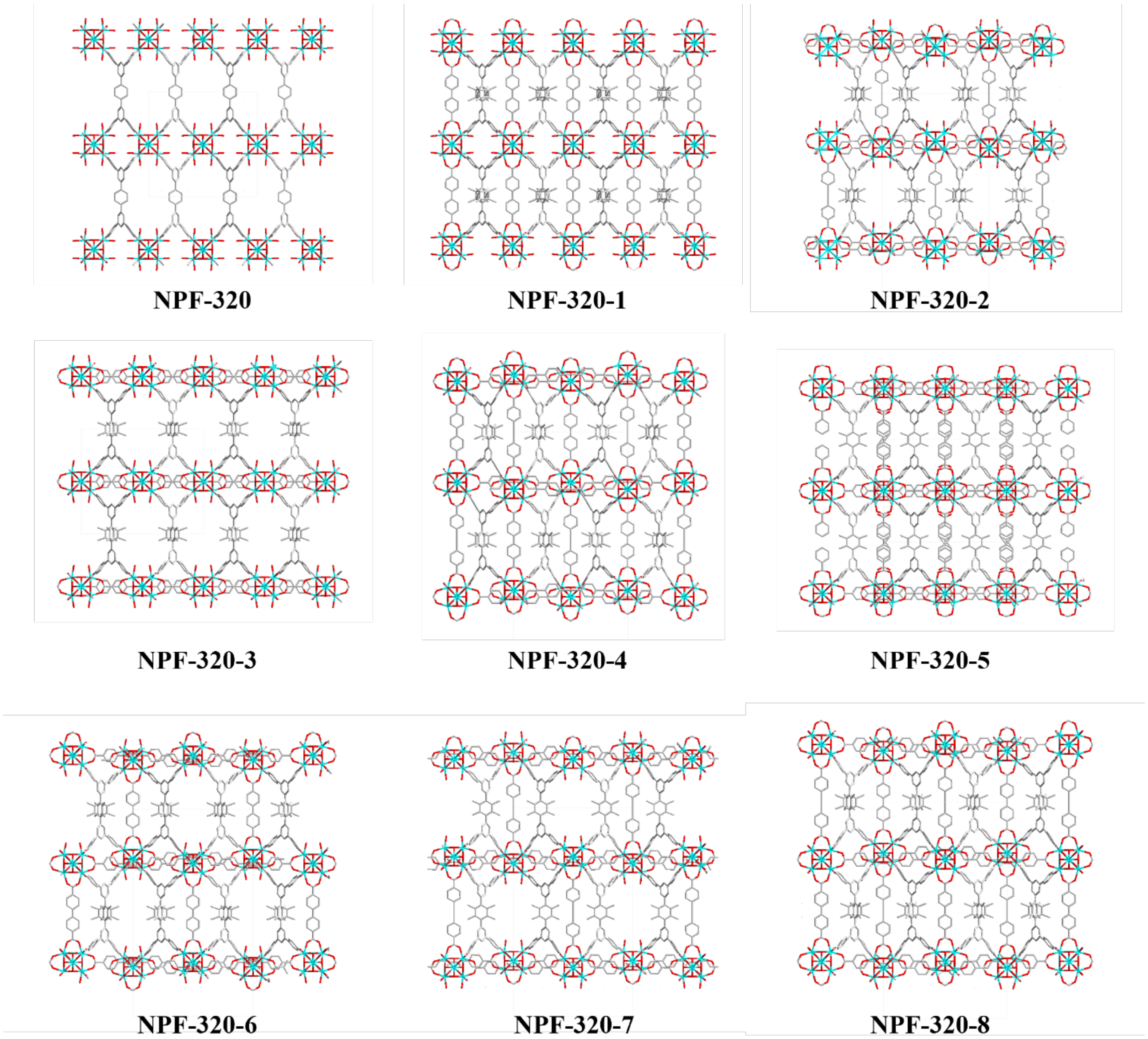

Figure S5. Crystal structures of NPF-320 Series viewed along the *b* axis.

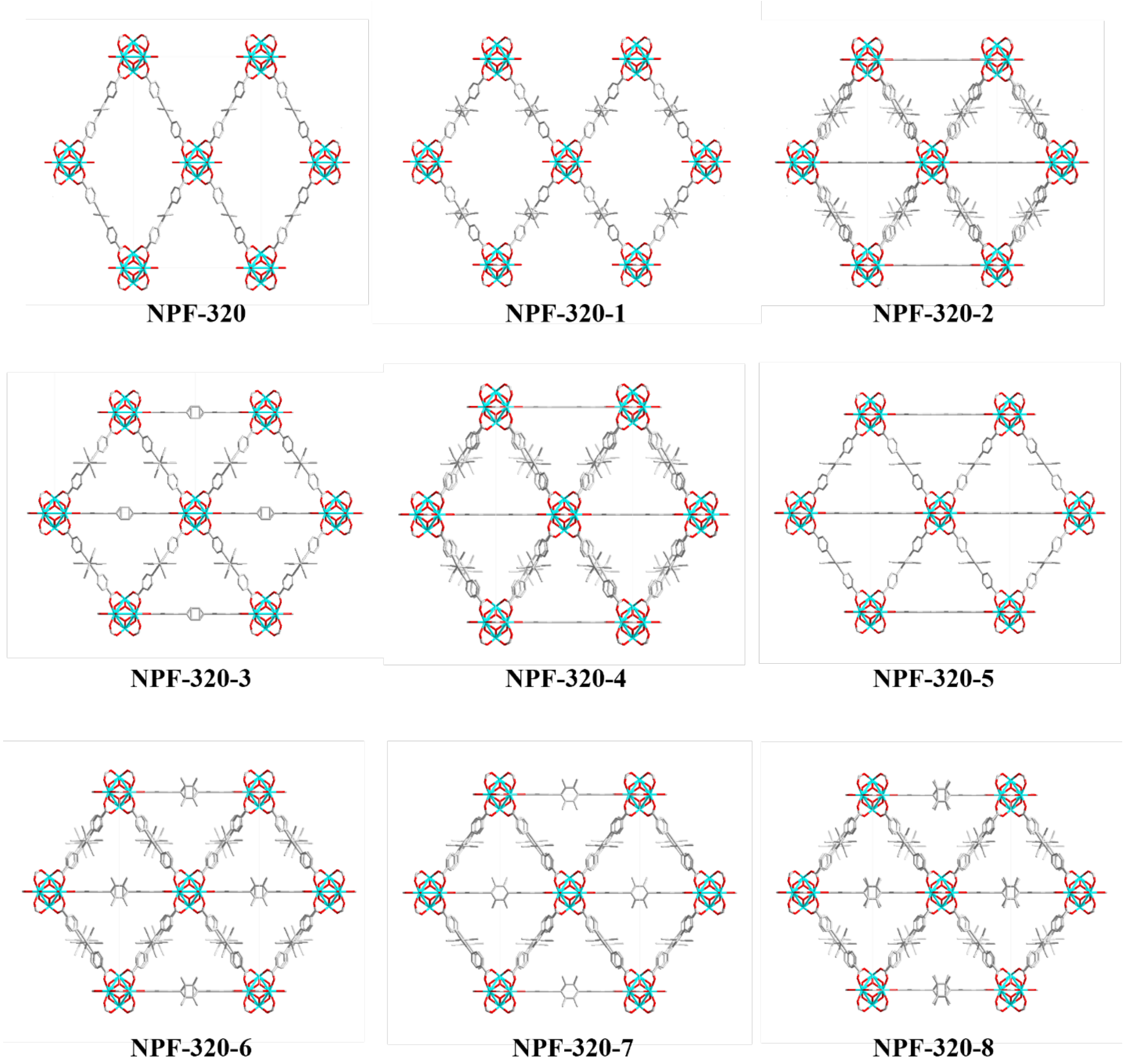

Figure S6. Crystal structures of NPF-320 Series viewed along the *c* axis.

S-5 NMR Digestion of Insertion Routes

To further support the linker insertion and exchange within MTV-NPF-320, the molar ratio of primary linkers and secondary linkers within MOFs were determined by base and acid digestion (detailed procedures are in S1), followed by ^{1}HNMR measurement. Each insertion route and all the molar ratios are listed in Table 1. Figures S7-21 show the ^{1}H NMR spectra obtained from the digestion of each insertion route.

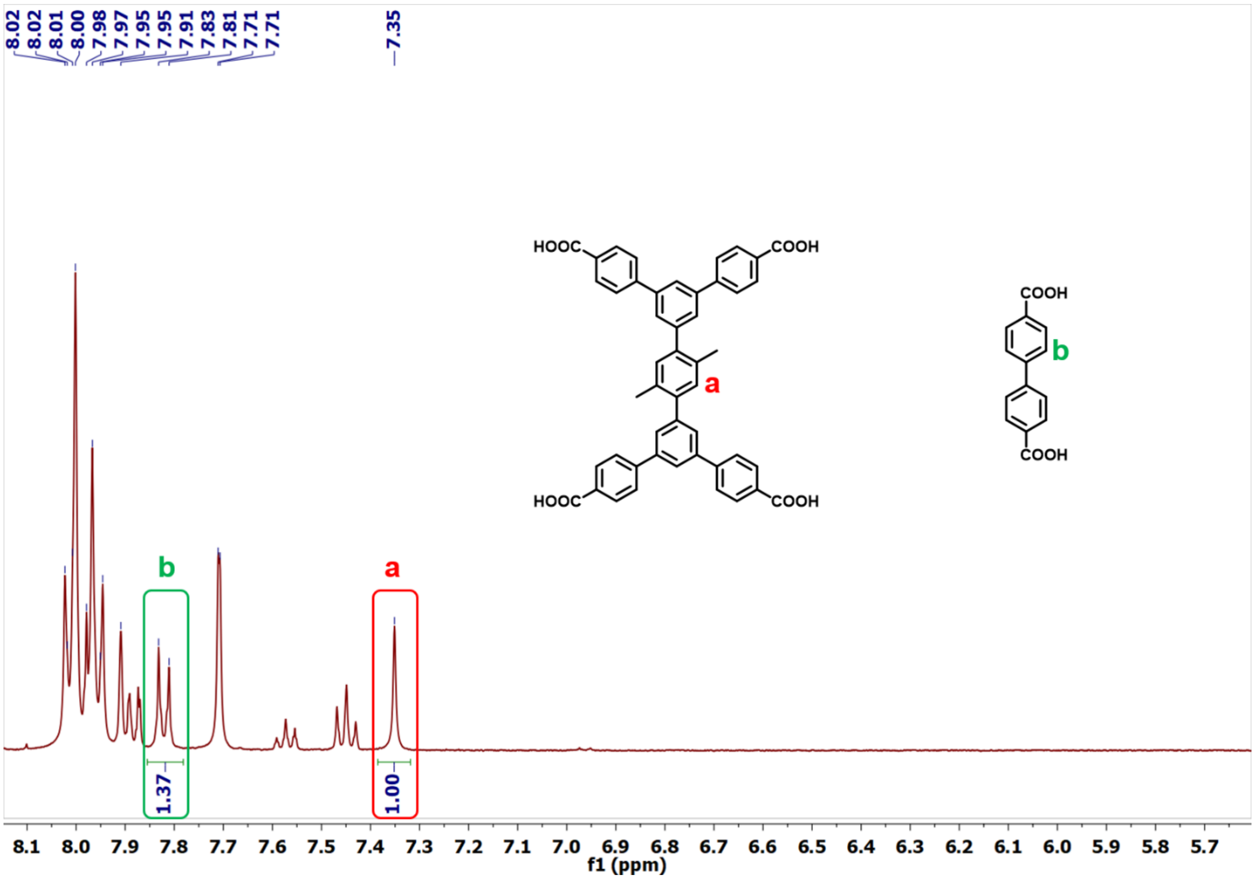

Figure S7. ^{1}H NMR spectrum of digested NPF-320-1 from insertion route 1. **L: sL$_1$** = 2.00: 1.37 (theoretical ratio= 2: 1).

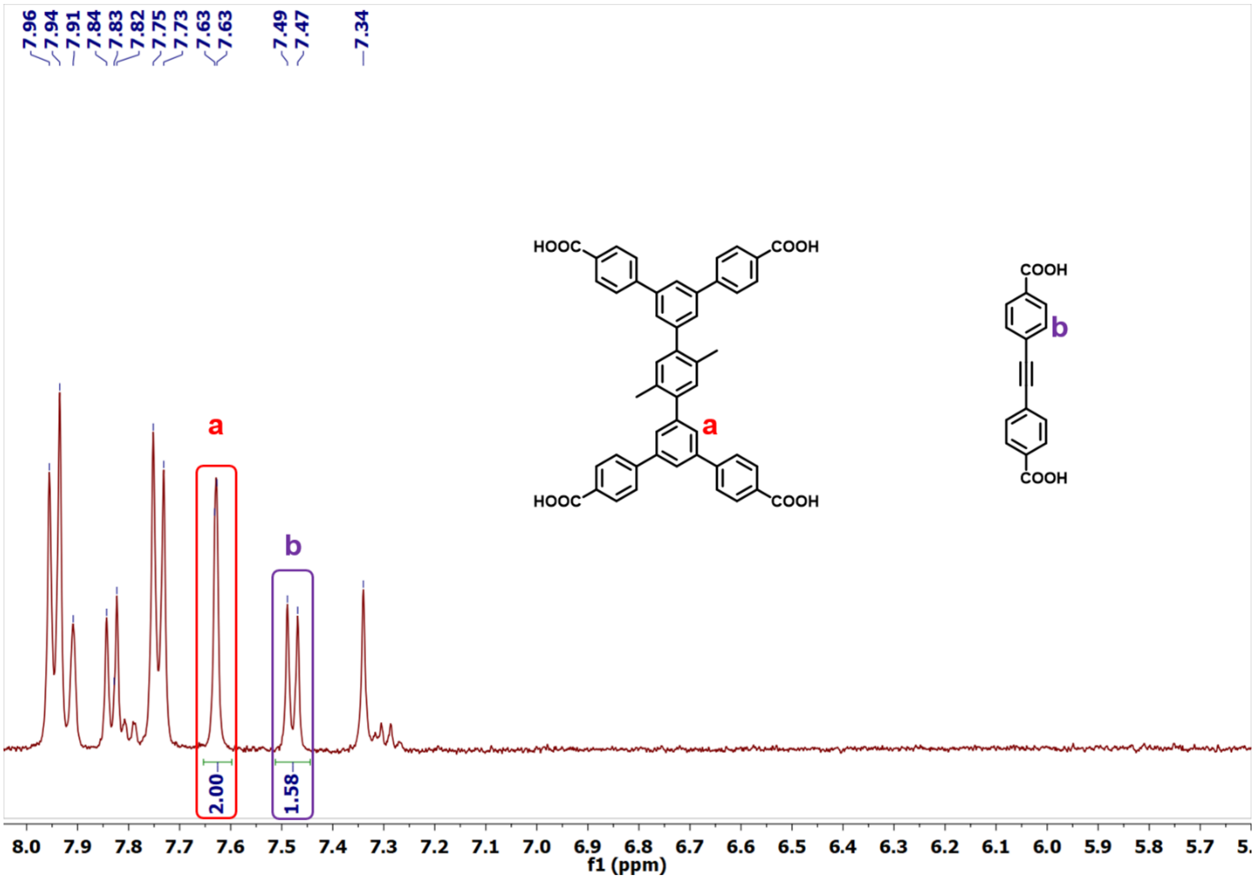

Figure S8. ^{1}H NMR spectrum of digested NPF-320-2 from insertion route 2. **L: sL$_2$** = 2.00: 1.58 (theoretical ratio = 2: 1.5).

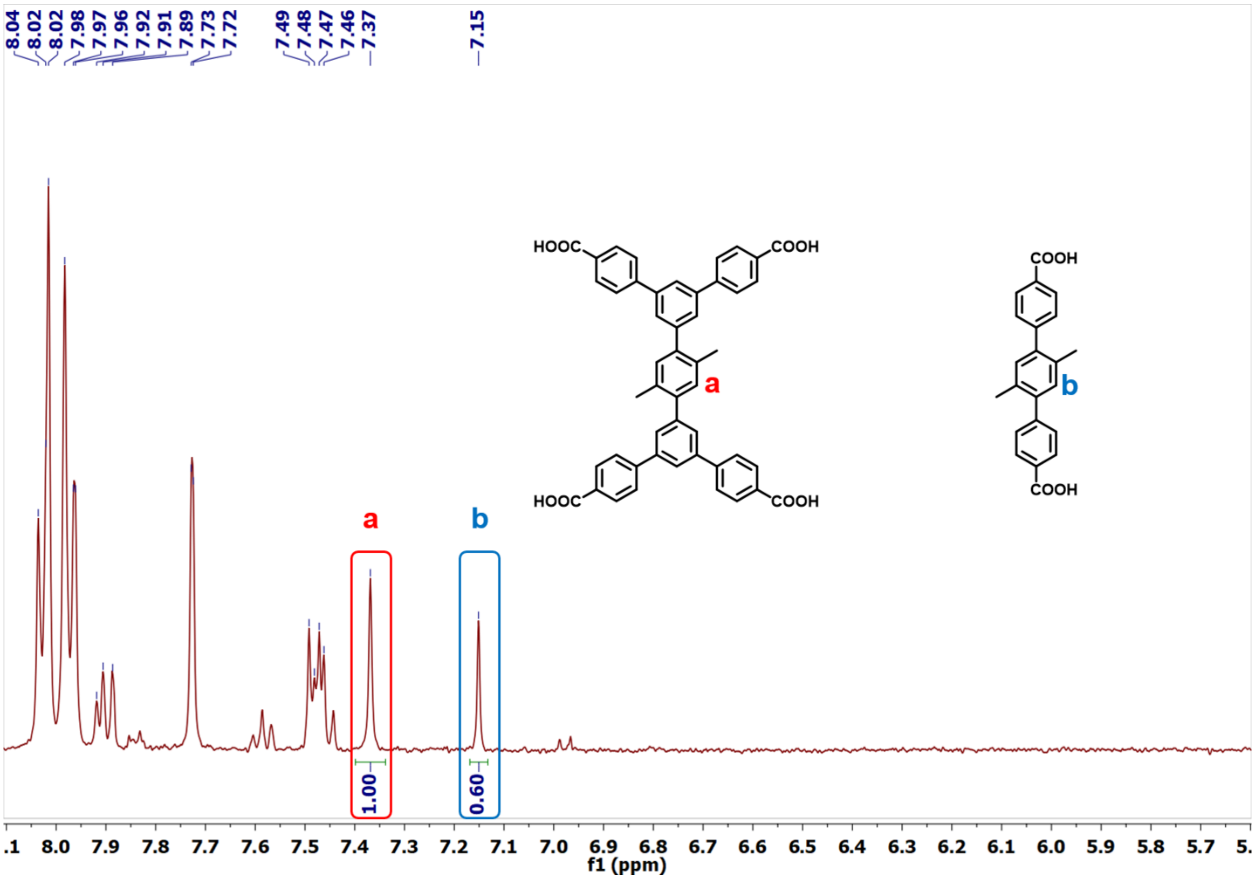

Figure S9. ^{1}H NMR spectrum of digested NPF-320-3 from insertion route 3. **L: sL$_3$** = 2.00: 1.20 (theoretical ratio = 2: 1).

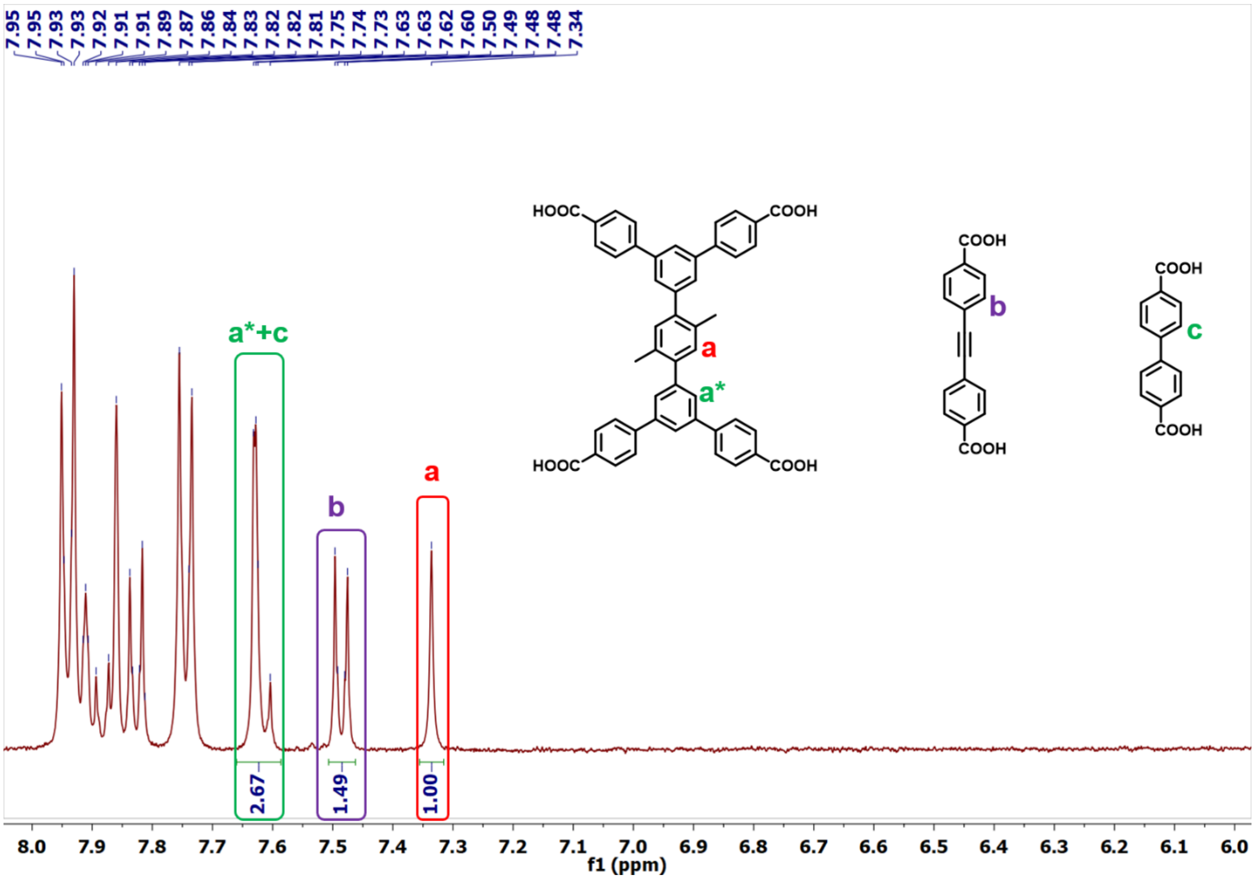

Figure S10. ^{1}H NMR spectrum of digested NPF-320-4 from insertion route 4. **L: sL_2: sL_1** = 2: 0.67: 1.49 (theoretical ratio = 2: 0.5: 1.5).

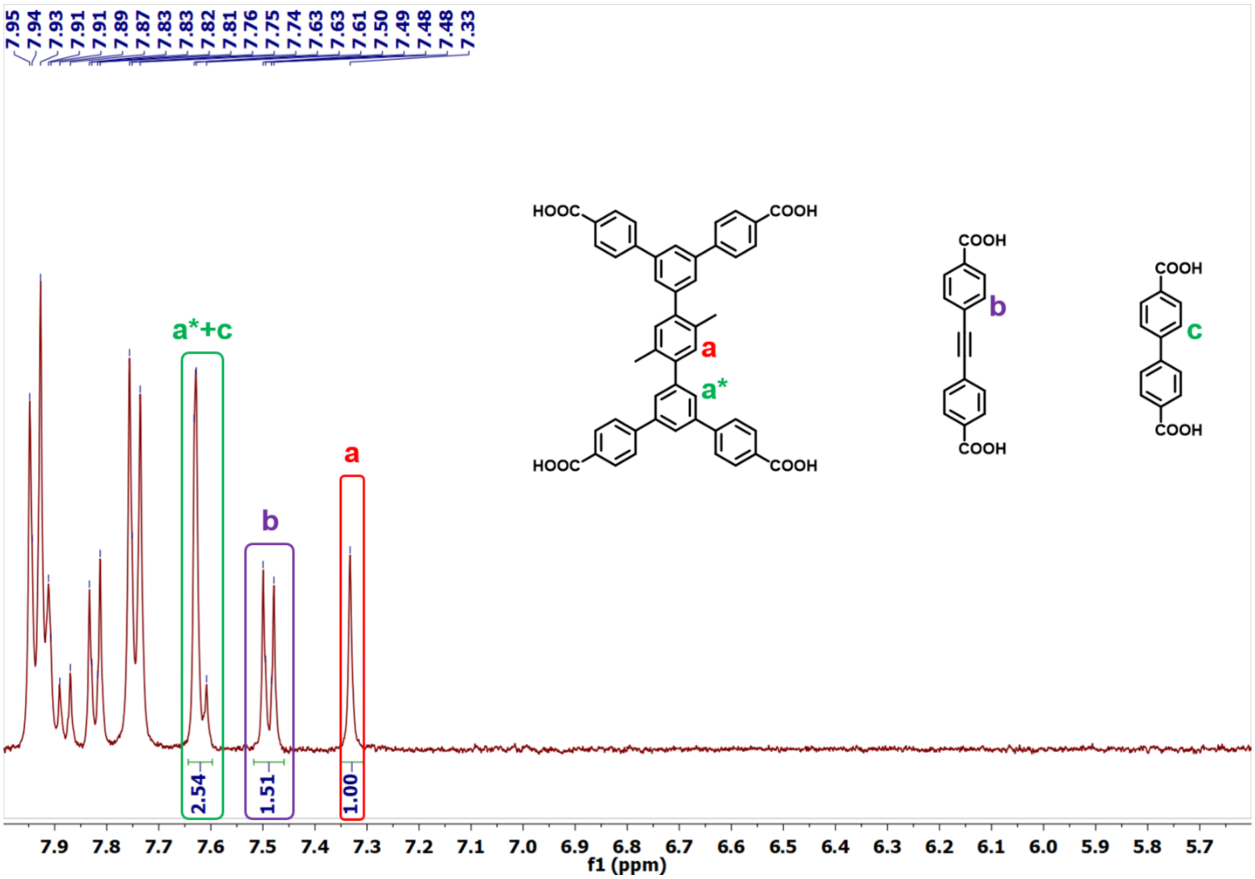

Figure S11. ^{1}H NMR spectrum of digested NPF-320-4 from insertion route 5. **L: sL_1: sL_2** = 2: 1.51: 0.54 (theoretical ratio = 2: 1.5: 0.5).

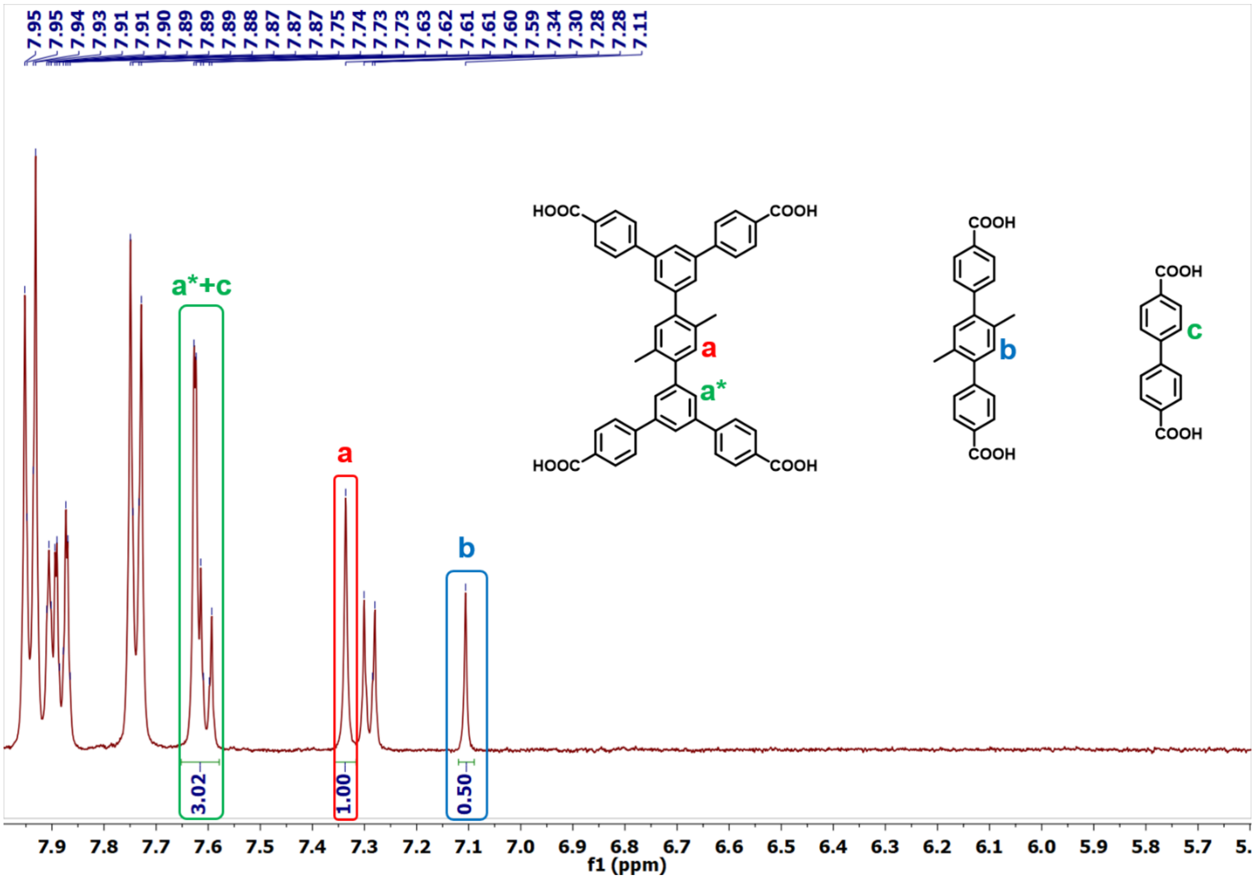

Figure S12. ^{1}H NMR spectrum of digested NPF-320-5 from insertion route 6. **L: sL$_1$: sL$_3$** = 2: 1.02: 1.00 (theoretical ratio = 2: 1: 1).

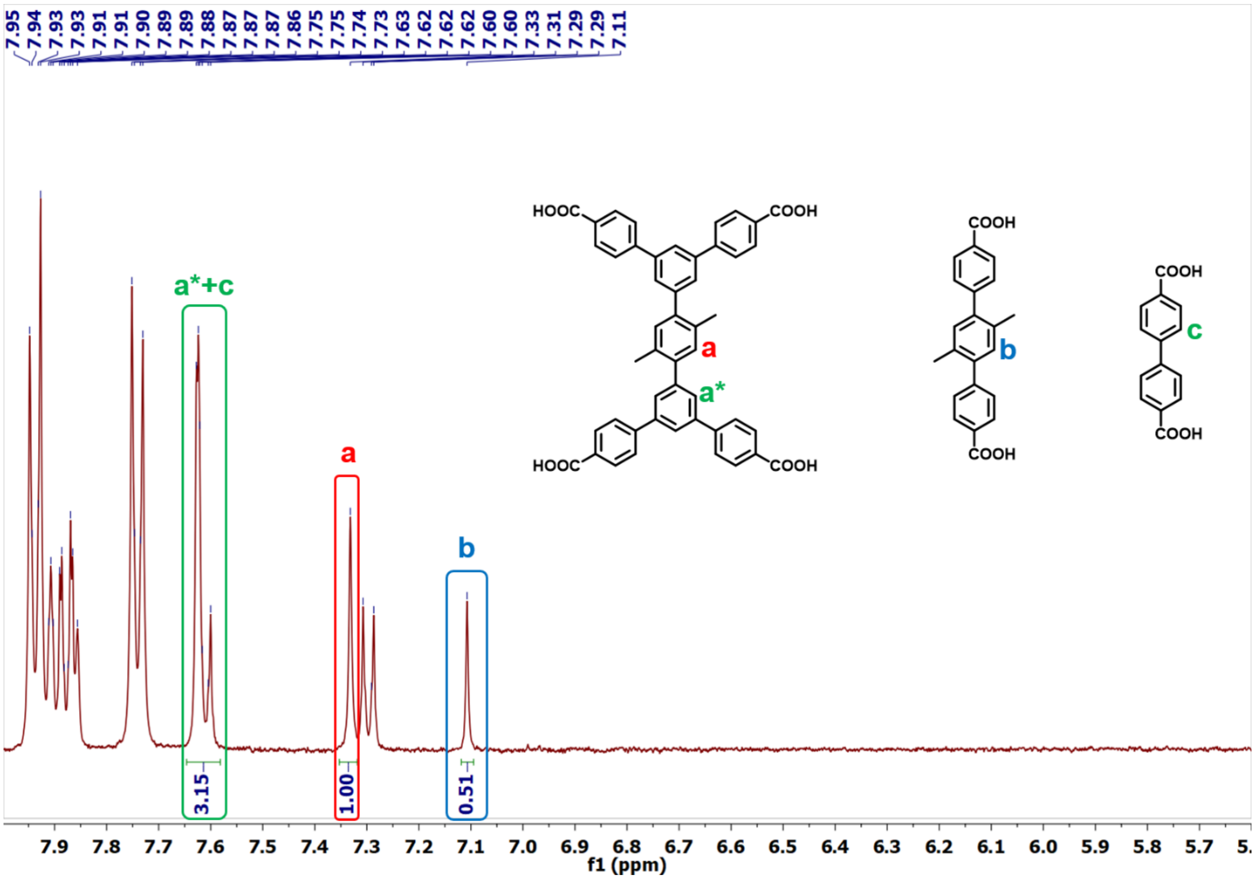

Figure S13. ^{1}H NMR spectrum of digested NPF-320-6 from insertion route 7. **L: sL$_3$: sL$_1$**= 2: 1.02: 1.15 (theoretical ratio = 2: 1: 0.5).

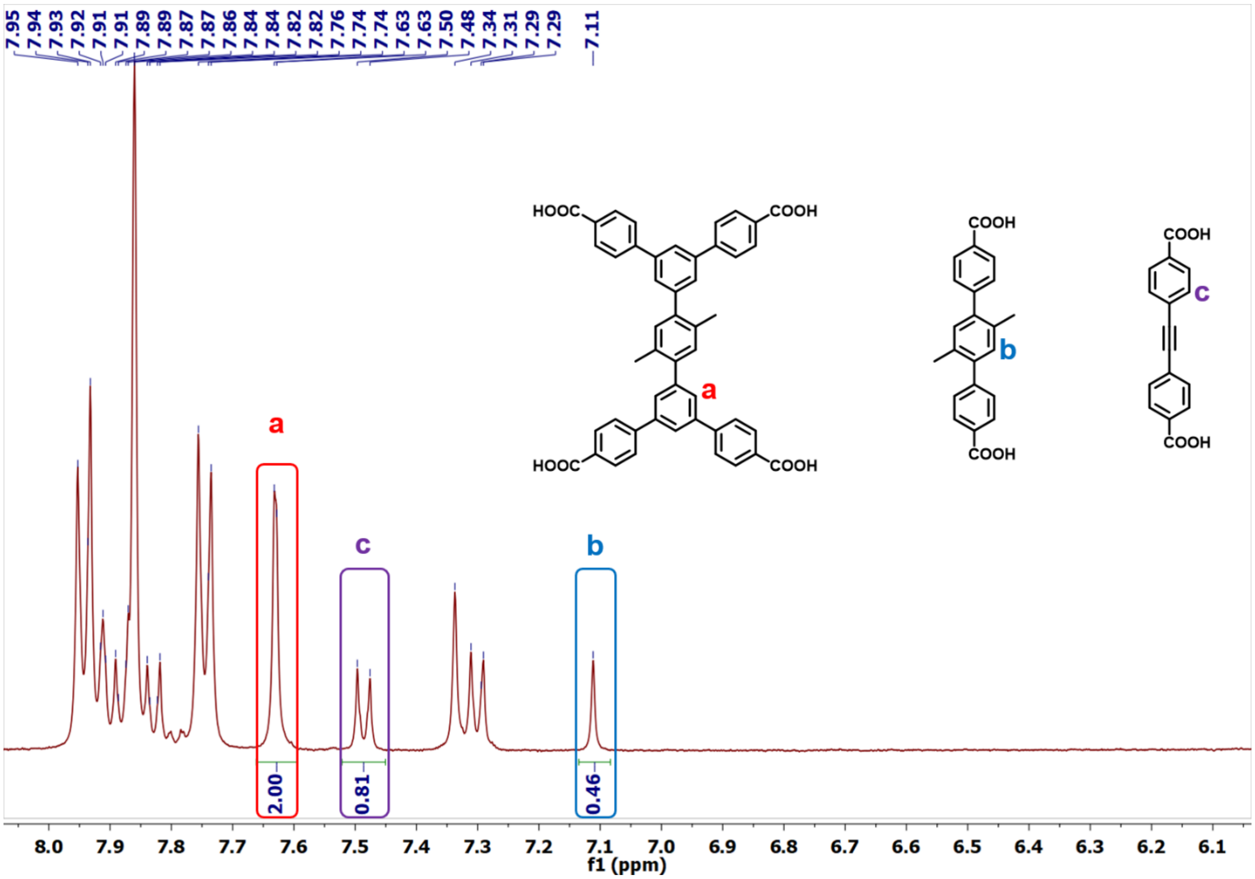

Figure S14. ^{1}H NMR spectrum of digested NPF-320-7 from insertion route 8. **L: sL$_3$: sL$_2$** = 2: 0.92: 0.81 (theoretical ratio = 2: 1: 0.5).

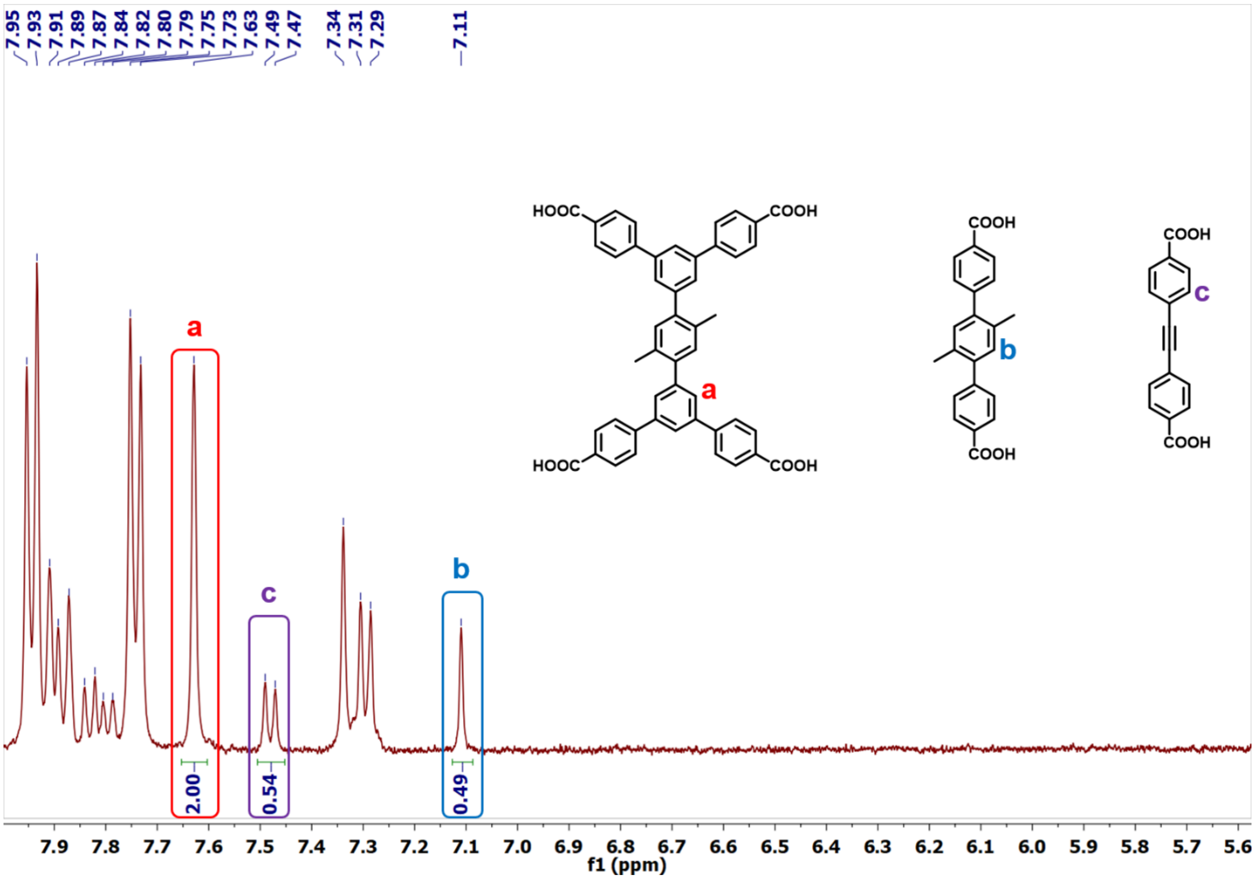

Figure S15. ^{1}H NMR spectrum of digested NPF-320-7 from insertion route 9. **L: sL$_2$: sL$_3$** = 2: 0.54: 0.98 (theoretical ratio = 2: 0.5: 1).

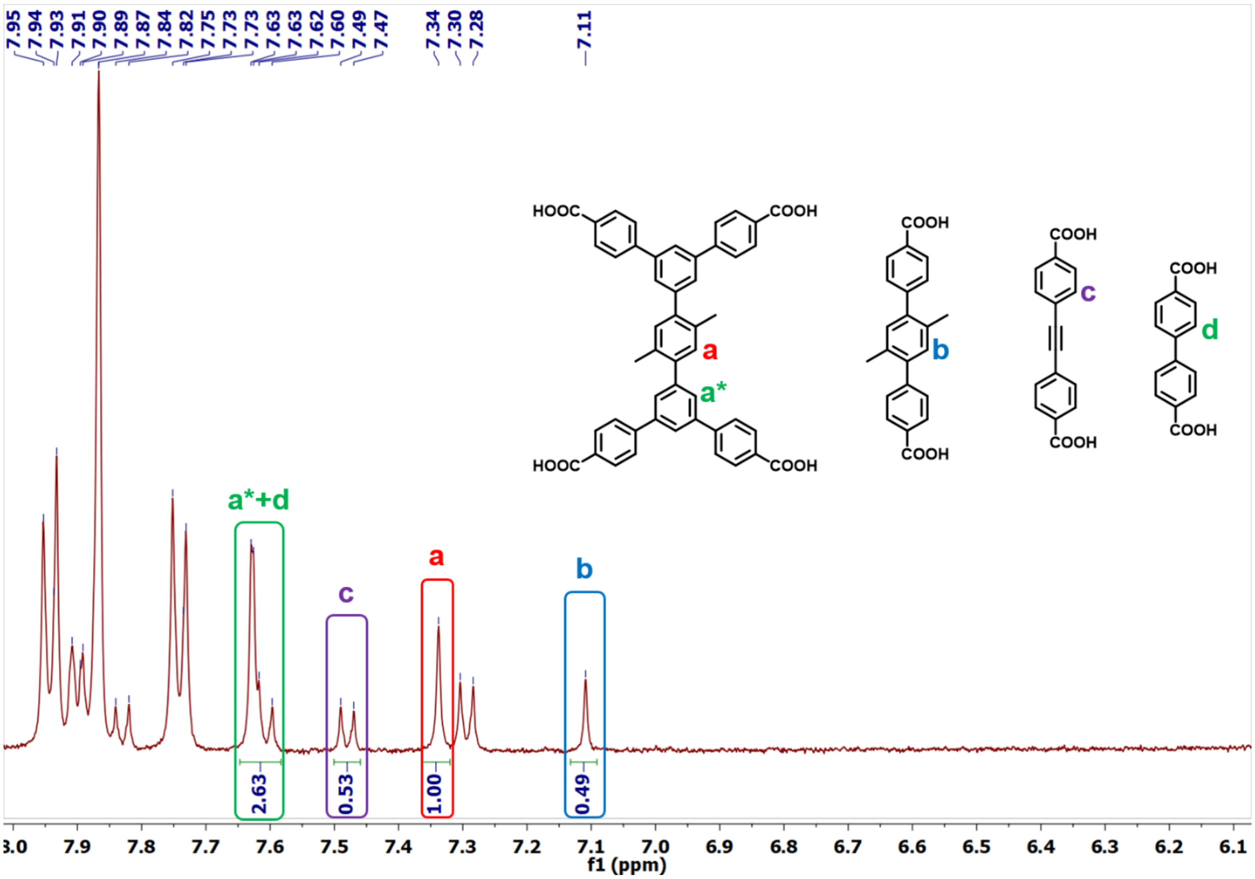

Figure S16. ^{1}H NMR spectrum of digested NPF-320-8 from insertion route 10. **L: sL$_3$: sL$_2$: sL$_1$** = 2: 0.98: 0.53: 0.63 (theoretical ratio = 2: 1: 0.5: 0.5).

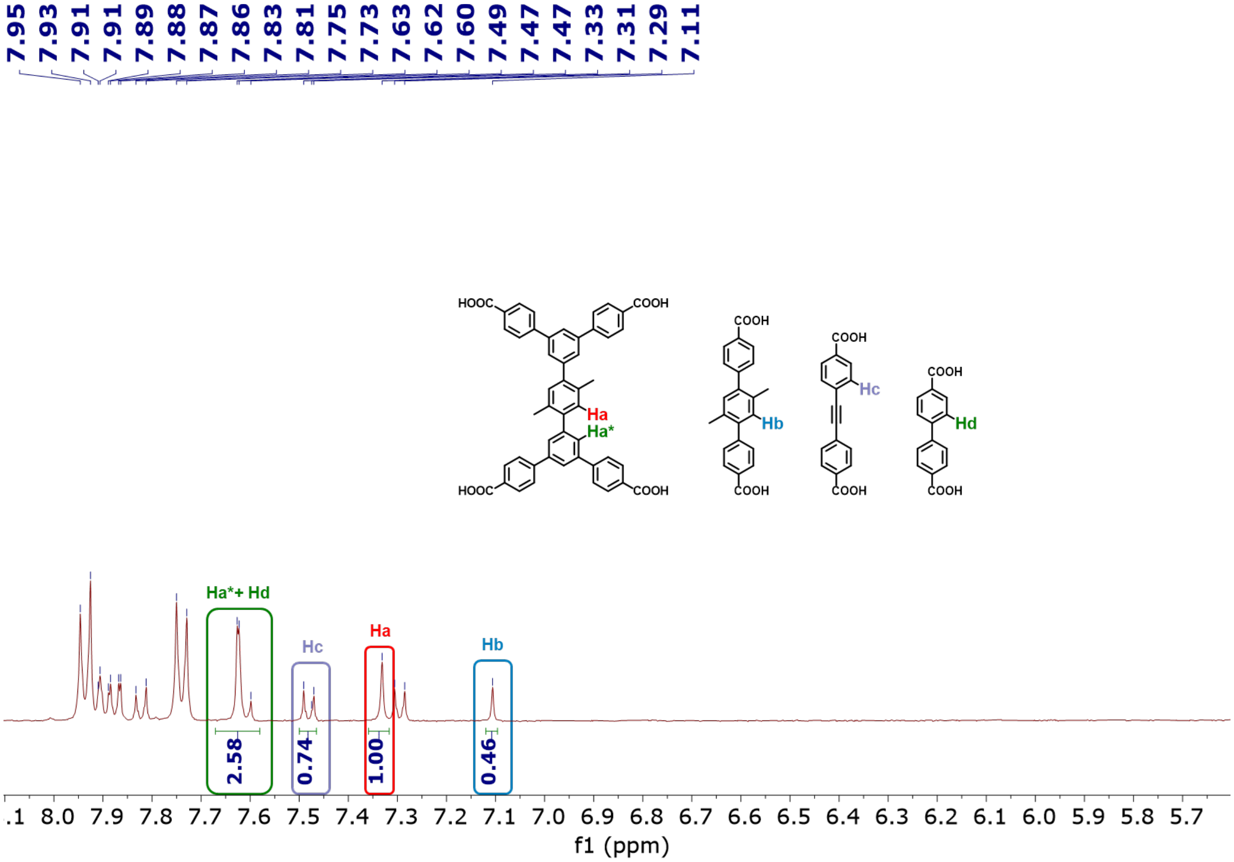

Figure S17. ^{1}H NMR spectrum of digested NPF-320-8 from insertion route 11. **L: sL$_2$: sL$_3$: sL$_1$** = 2.00:0.74 :0.92 :0.58 (theoretical ratio = 2: 0.5: 1: 0.5).

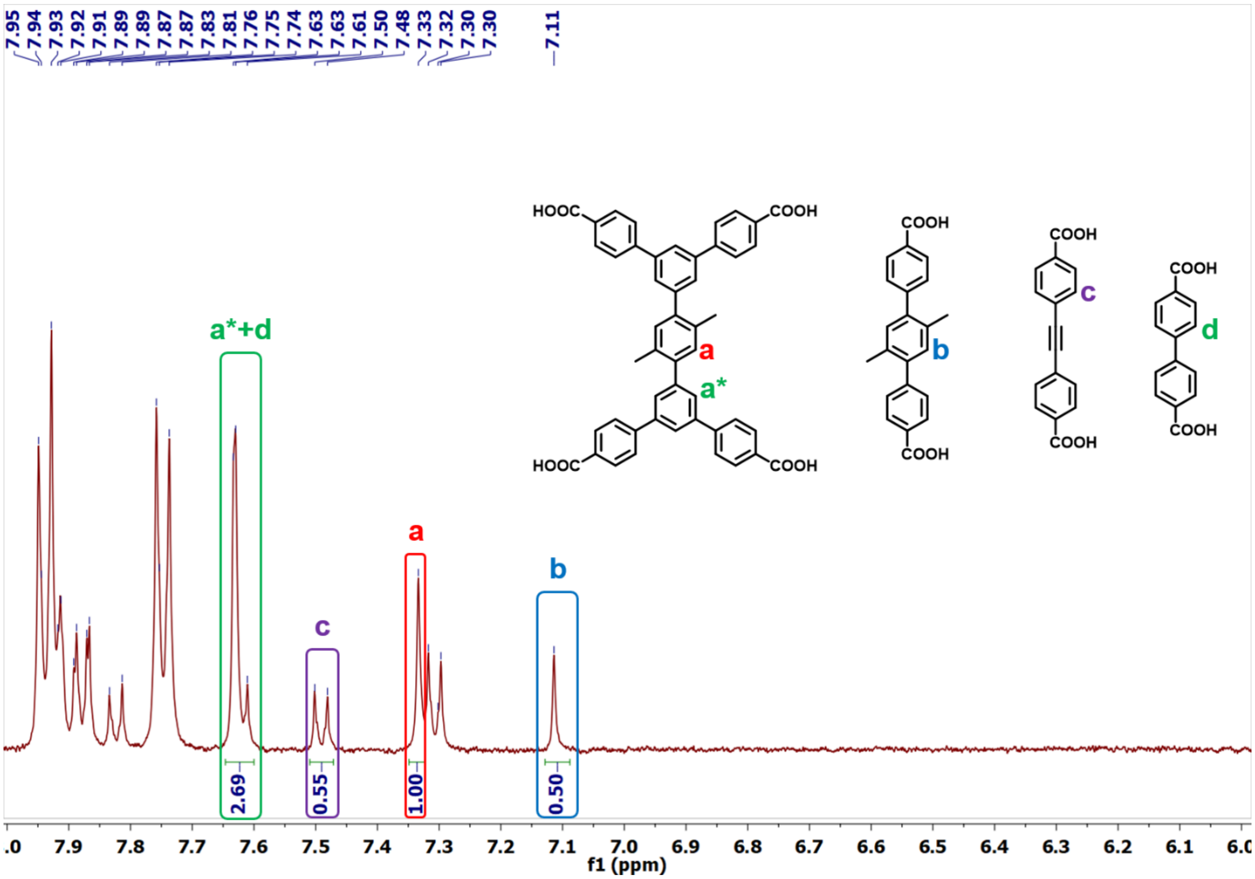

Figure S18. ^{1}H NMR spectrum of digested NPF-320-8 from insertion route 12. **L: sL$_3$: sL$_1$: sL$_2$** = 2: 1.00: 0.69: 0.55 (theoretical ratio = 2: 1: 0.5: 0.5).

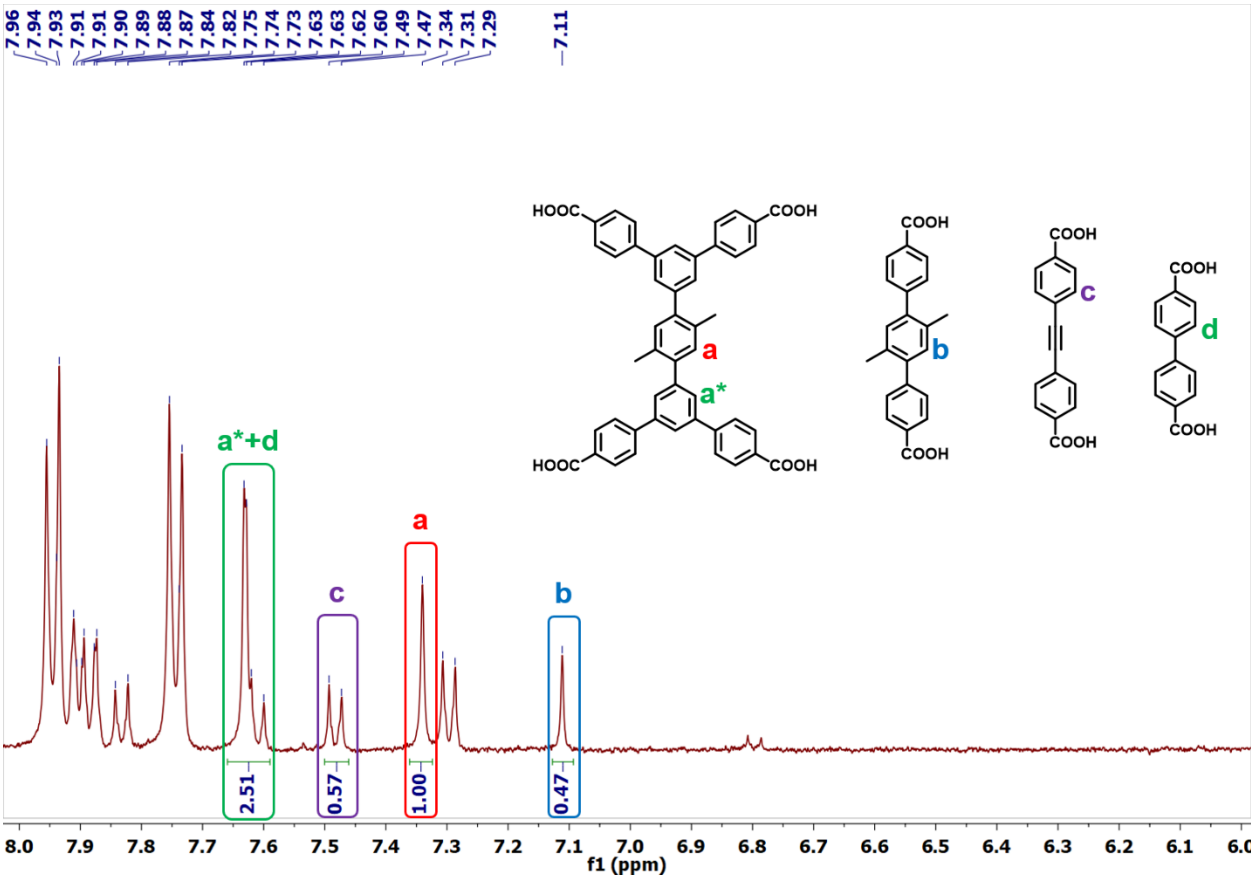

Figure S19. ^{1}H NMR spectrum of digested NPF-320-8 from insertion route 13. **L: sL$_1$: sL$_3$: sL$_2$** = 2: 0.51: 0.94: 0.57 (theoretical ratio = 2: 0.5: 1: 0.5).

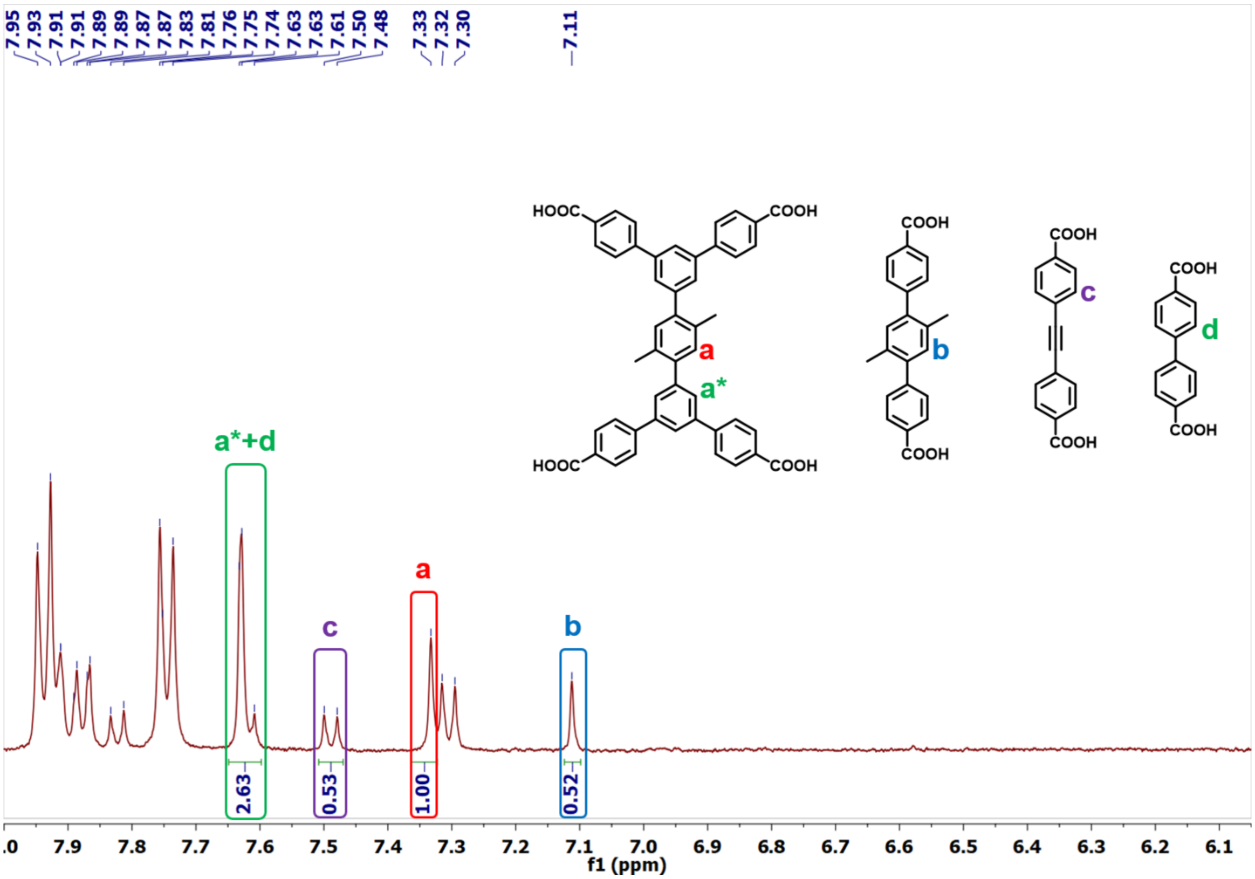

Figure S20. ^{1}H NMR spectrum of digested NPF-320-8 from insertion route 14. **L: sL_2: sL_1: sL_3** = 2: 0.53: 0.63: 1.04 (theoretical ratio = 2: 0.5: 0.5: 1).

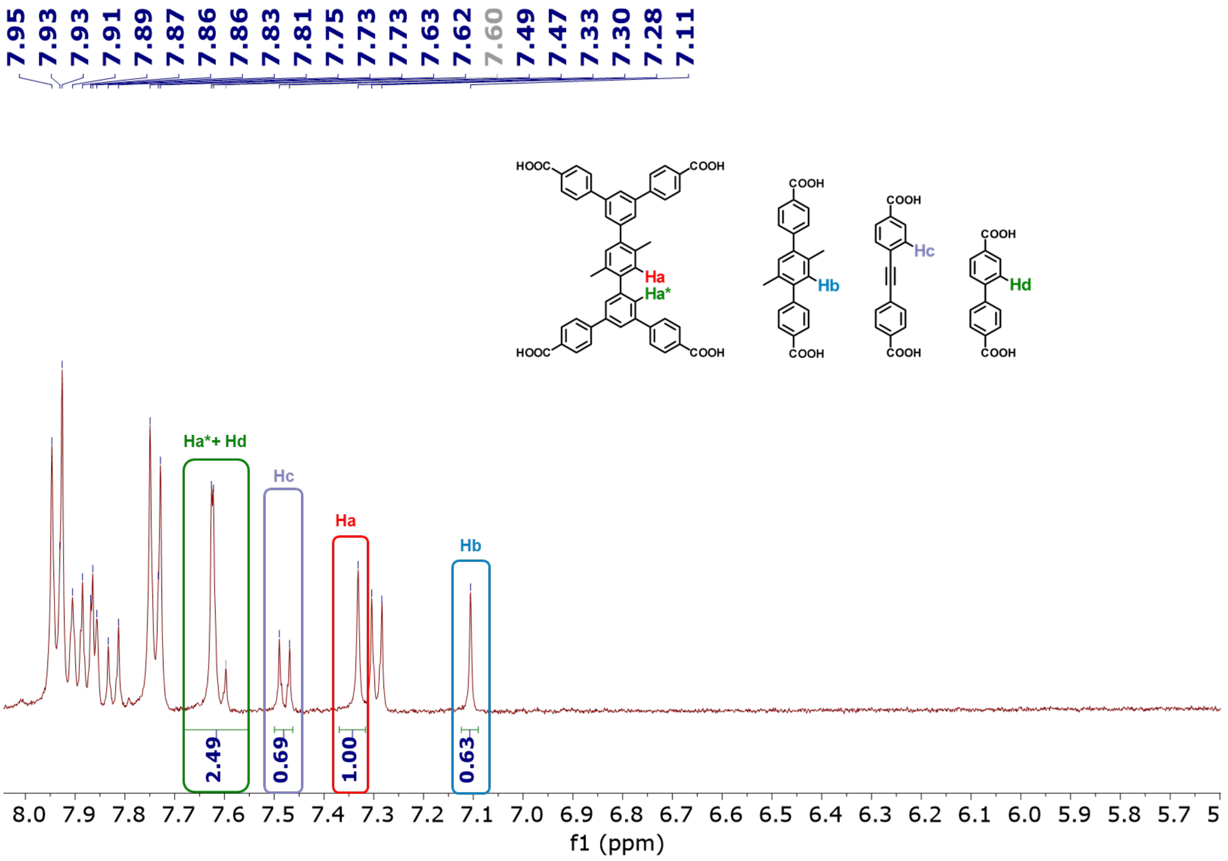

Figure S21. ^{1}H NMR spectrum of digested NPF-320-8 from insertion route 15. **L: sL$_1$: sL$_2$: sL$_3$** = 2: 0.49: 0.69: 1.26 (theoretical ratio = 2: 0.5: 0.5: 1).

S-6 Powder X-Ray Diffraction

Figures S22-23 show the experimental powder X-ray diffraction (PXRD) patterns of the solvated NPF-320 series, which exhibit an excellent agreement with the simulation from the refined single crystals structures and confirm the bulk purity of the NPF-320 and MOFs after **sLs** insertion and exchange.

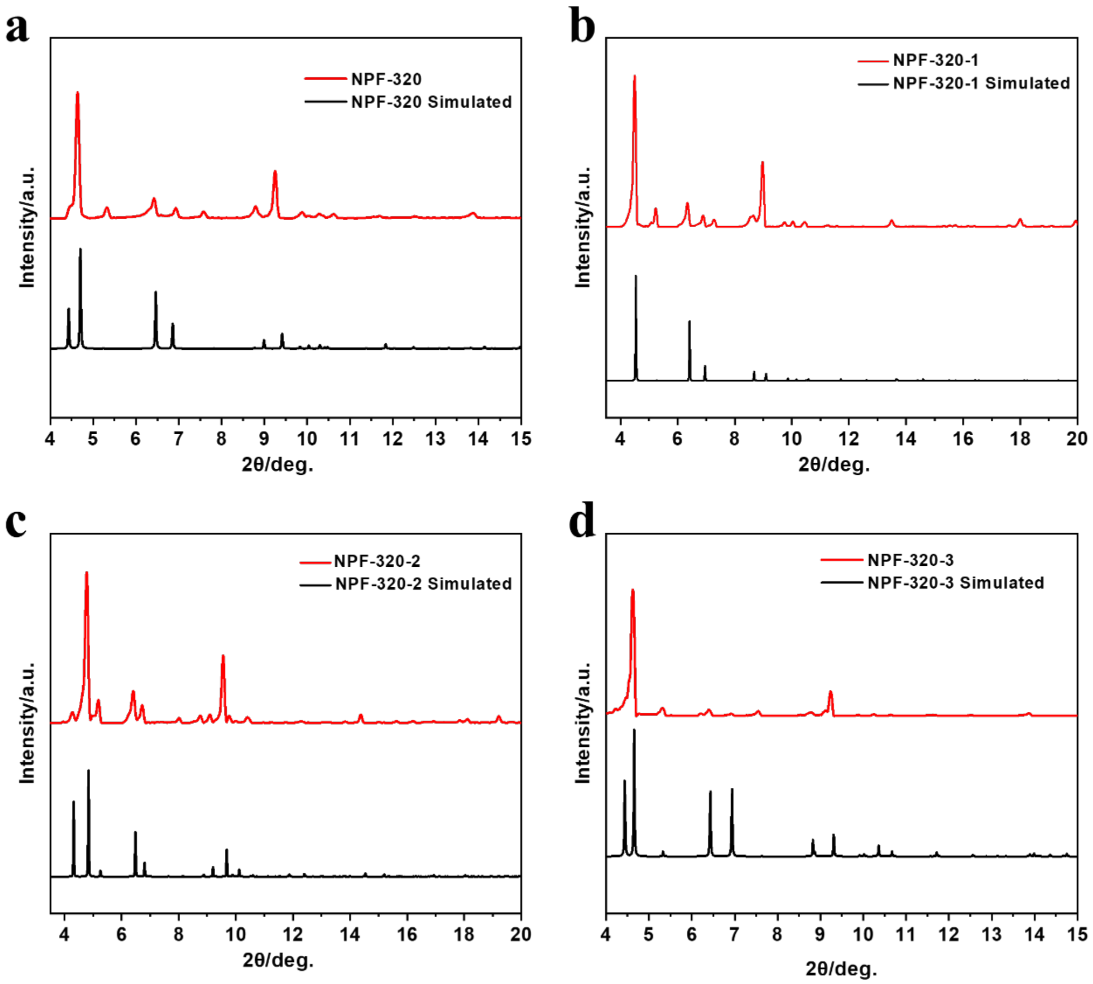

Figure S22. (a) PXRD of NPF-320. (b) PXRD of NPF-320-1. (c) PXRD of NPF-320-2. (d) PXRD of NPF-320-3. Note the simulated patterns have all the peaks show in the experimental patterns but some with very weak intensity.

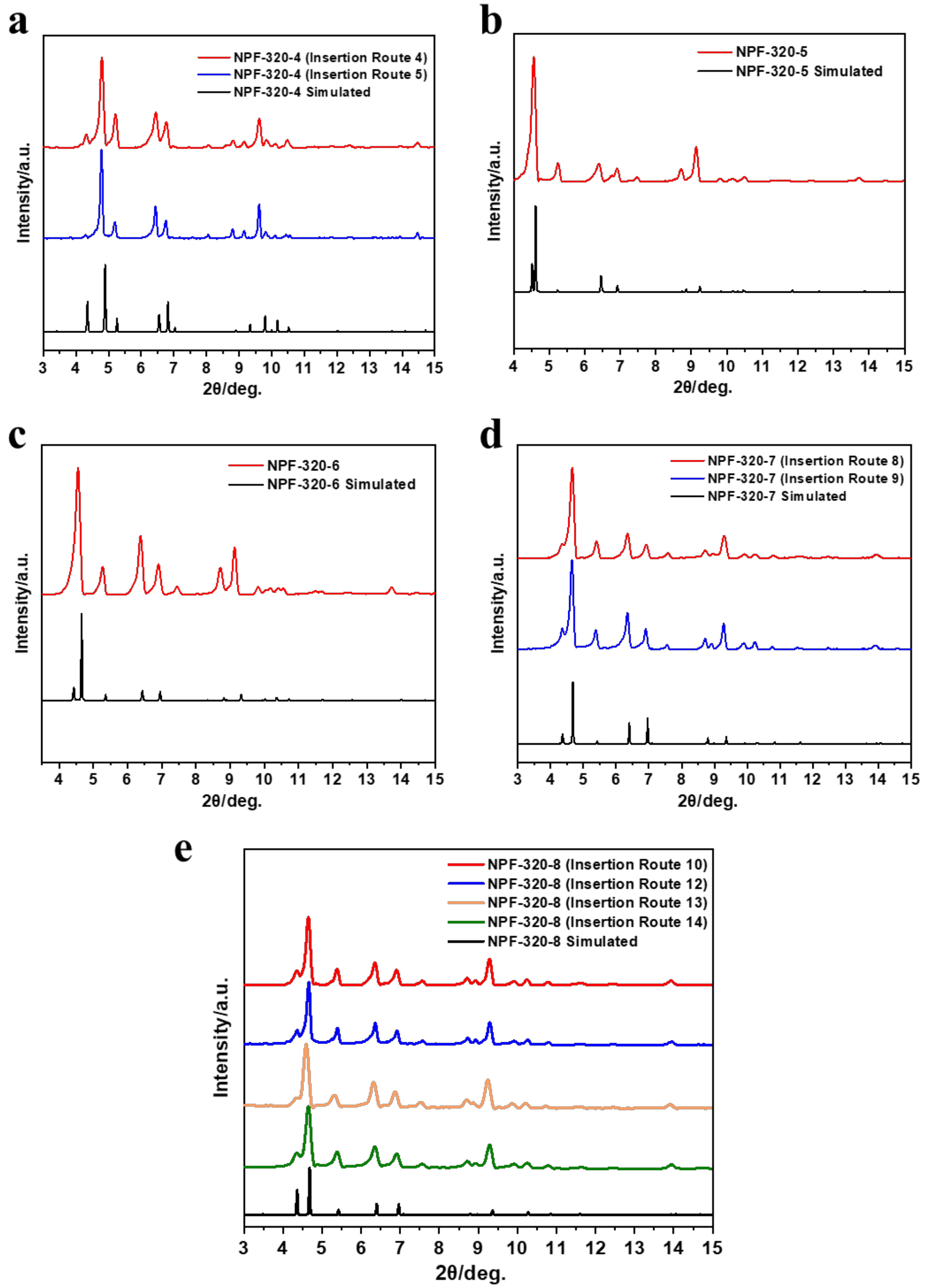

Figure S23. (a) PXRD of NPF-320-4. (b) PXRD of NPF-320-5. (c) PXRD of NPF-320-6. (d) PXRD of NPF-320-7. (e) PXRD of NPF-320-8. Note the simulated patterns have all the peaks show in the experimental patterns but some with very weak intensity.

S-7 Thermogravimetric Analysis

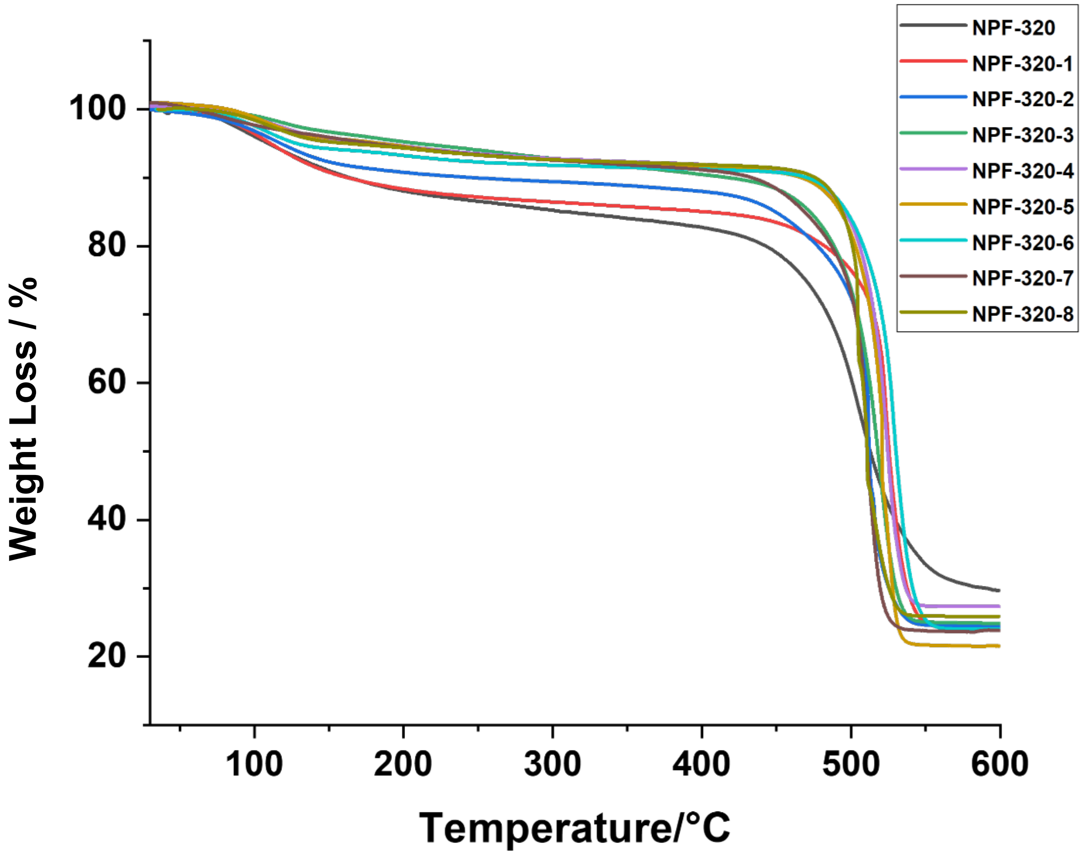

Figure S24. The TGA thermograms of NPF-320 series MOFs.

S-8 Stability of NPF-320 Series

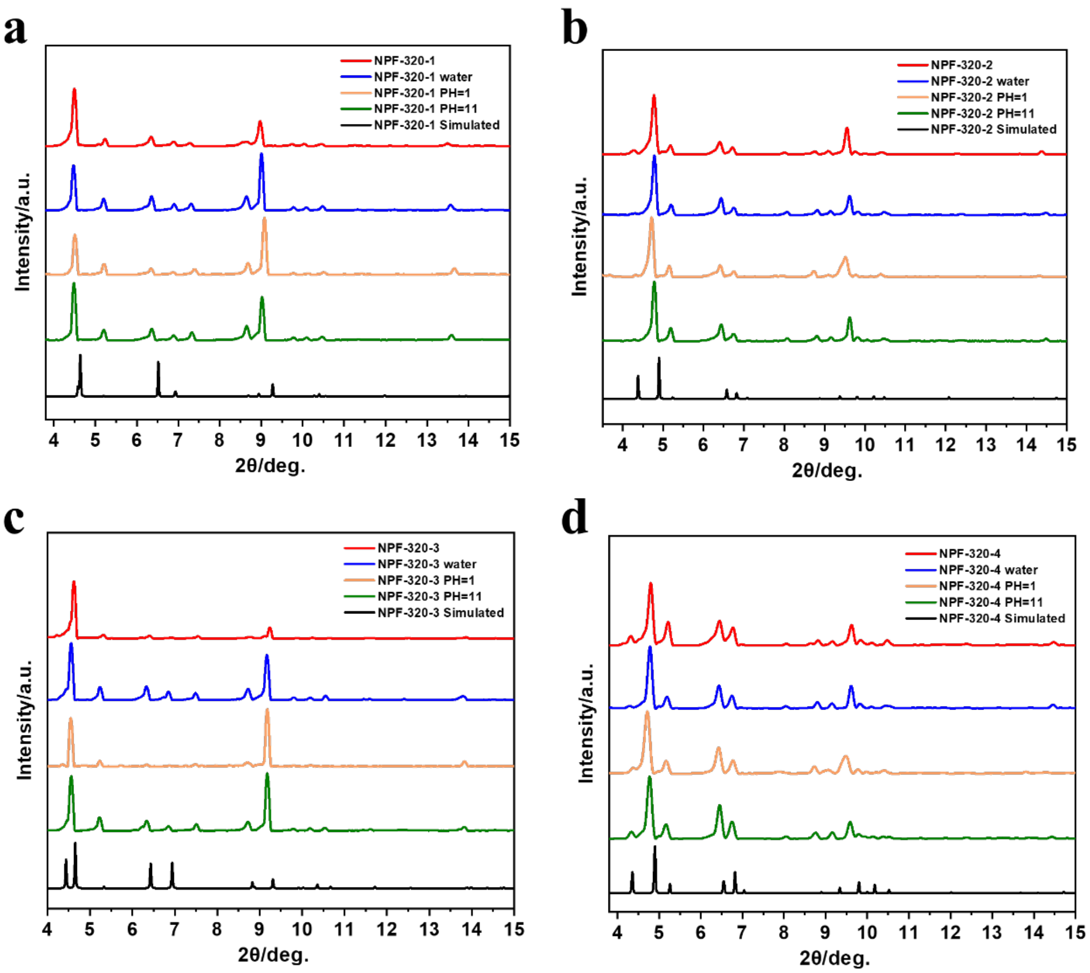

Figure S25. PXRD of (a) NPF-320-1, (b) NPF-320-2, (c) NPF-320-3, (d) NPF-320-4 after acid, base, and water treatment for 24 h.

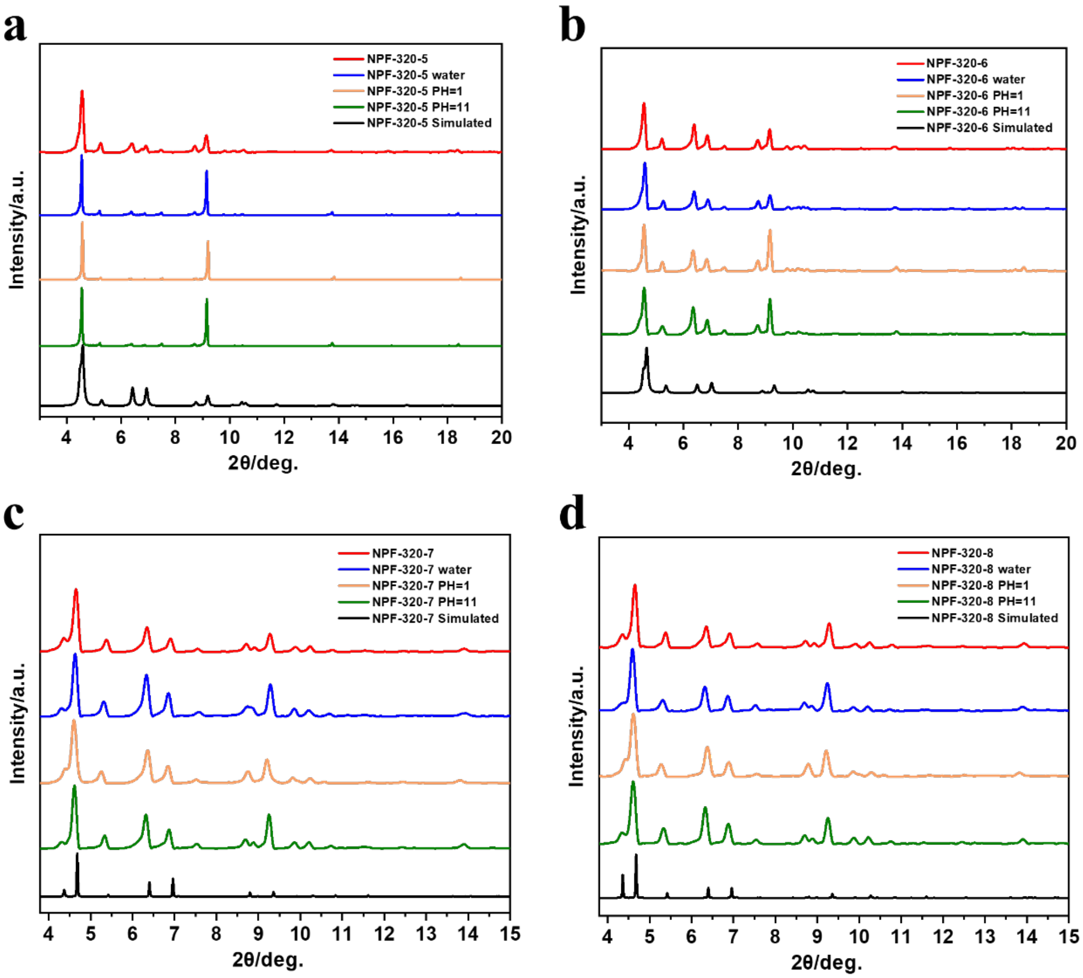

Figure S26. PXRD of (a) NPF-320-5, (b) NPF-320-6, (c) NPF-320-7, (d) NPF-320-8 after acid, base, and water treatment for 24 h.

The molar ratio of primary ligand and secondary linkers within MOFs after water, base, and acid treatments were determined by base digestion (detailed procedures are in S1), followed by ^{1}HNMR measurement. All the ratios are listed in Table 2, the NMR spectra are all shown below (Figures S27-34):

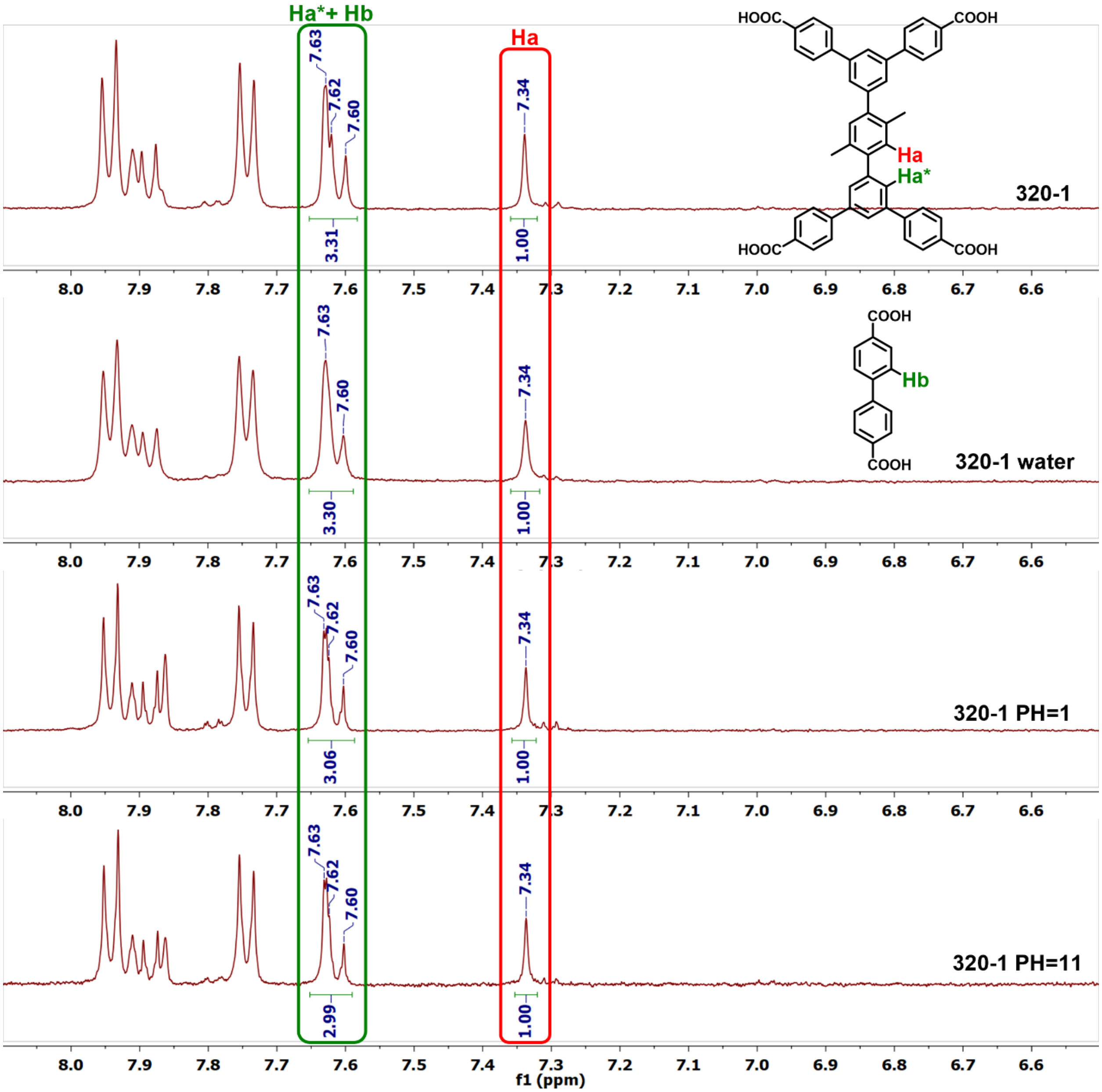

Figure S27. ^{1}H NMR spectrum of digested NPF-320-1 after acid, base, and water treatment for 24 h. (doublet peaks at 7.80 and 7.30 ppm are from benzoic anion)

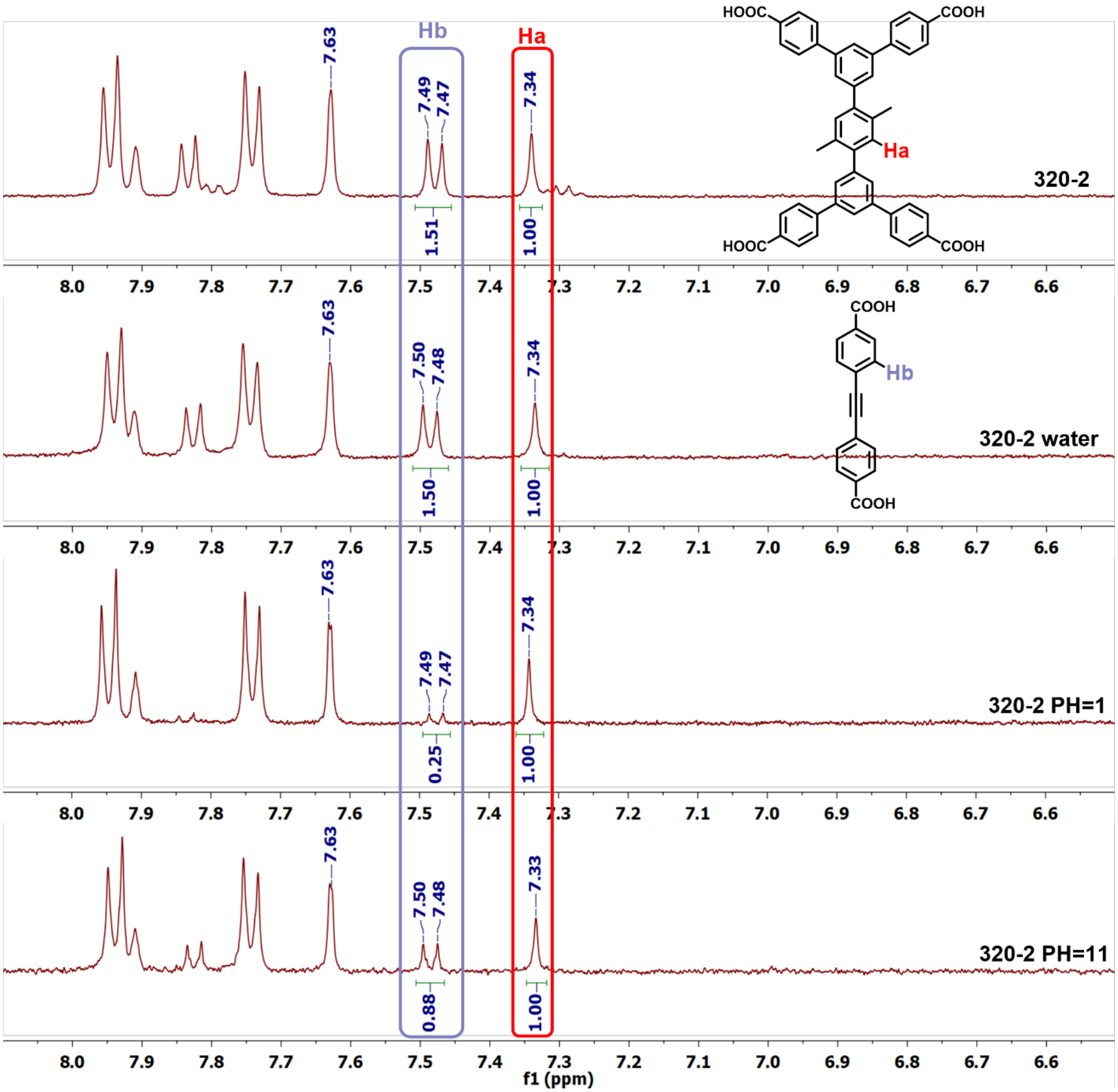

Figure S28. ^{1}H NMR spectrum of digested NPF-320-2 after acid, base, and water treatment for 24 h. (doublet peaks at 7.80 ppm are from benzoic anion)

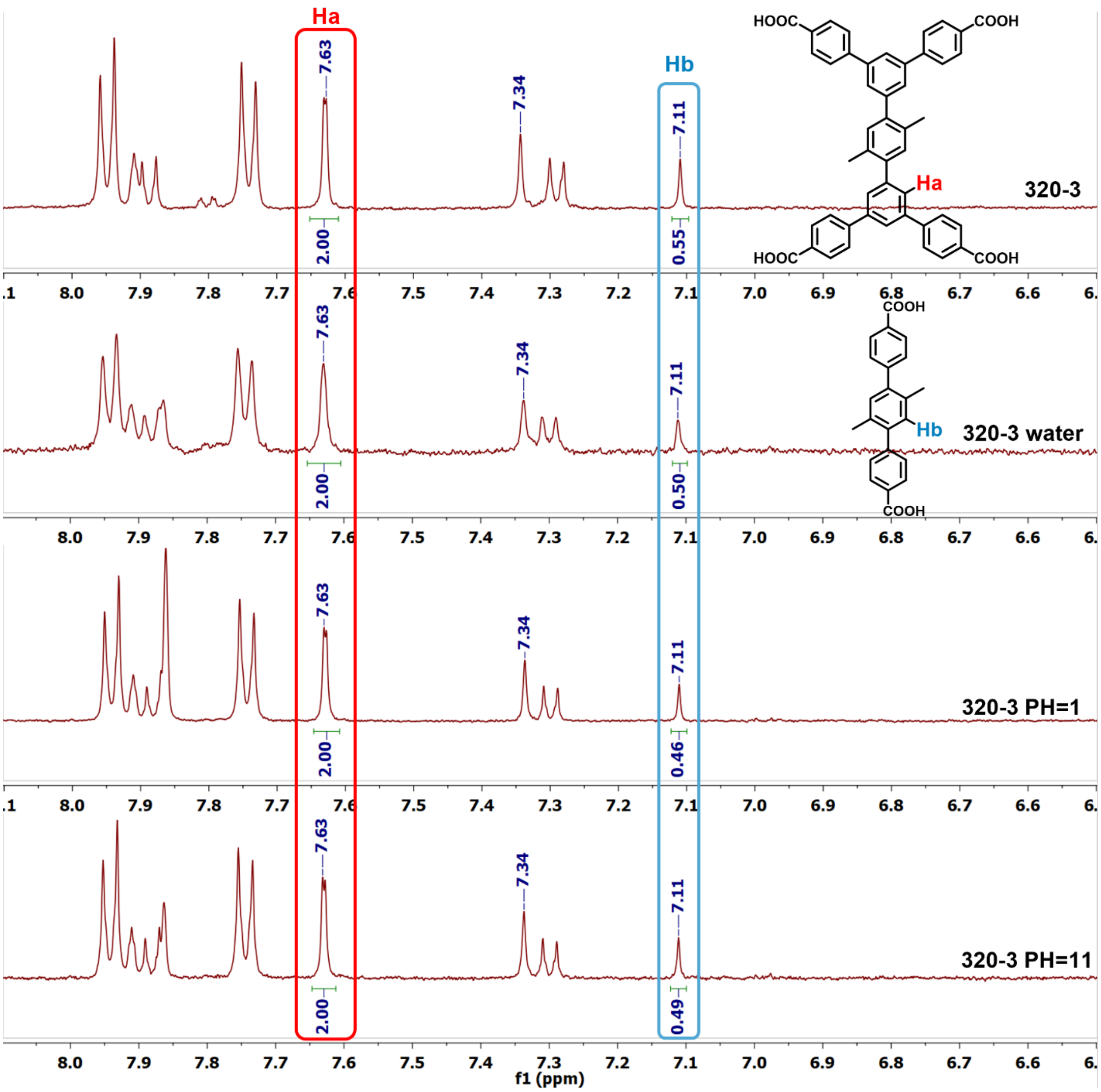

Figure S29. ^{1}H NMR spectrum of digested NPF-320-3 after acid, base, and water treatment for 24 h. (doublet peaks at 7.80 and 7.30 ppm are from benzoic anion)

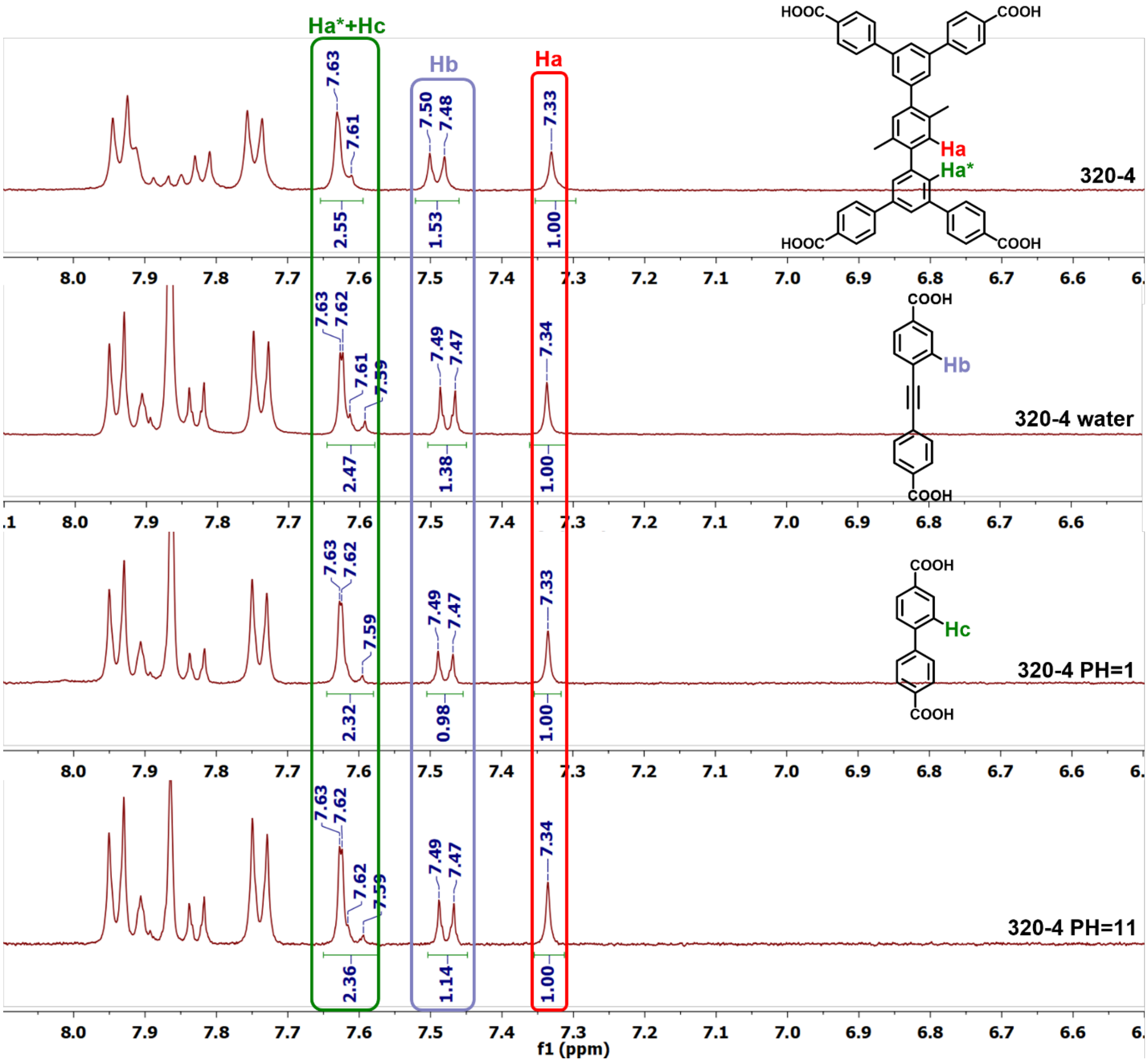

Figure S30. ^{1}H NMR spectrum of digested NPF-320-4 after acid, base, and water treatment for 24 h. (doublet peaks at 7.80 and 7.30 ppm are from benzoic anion)

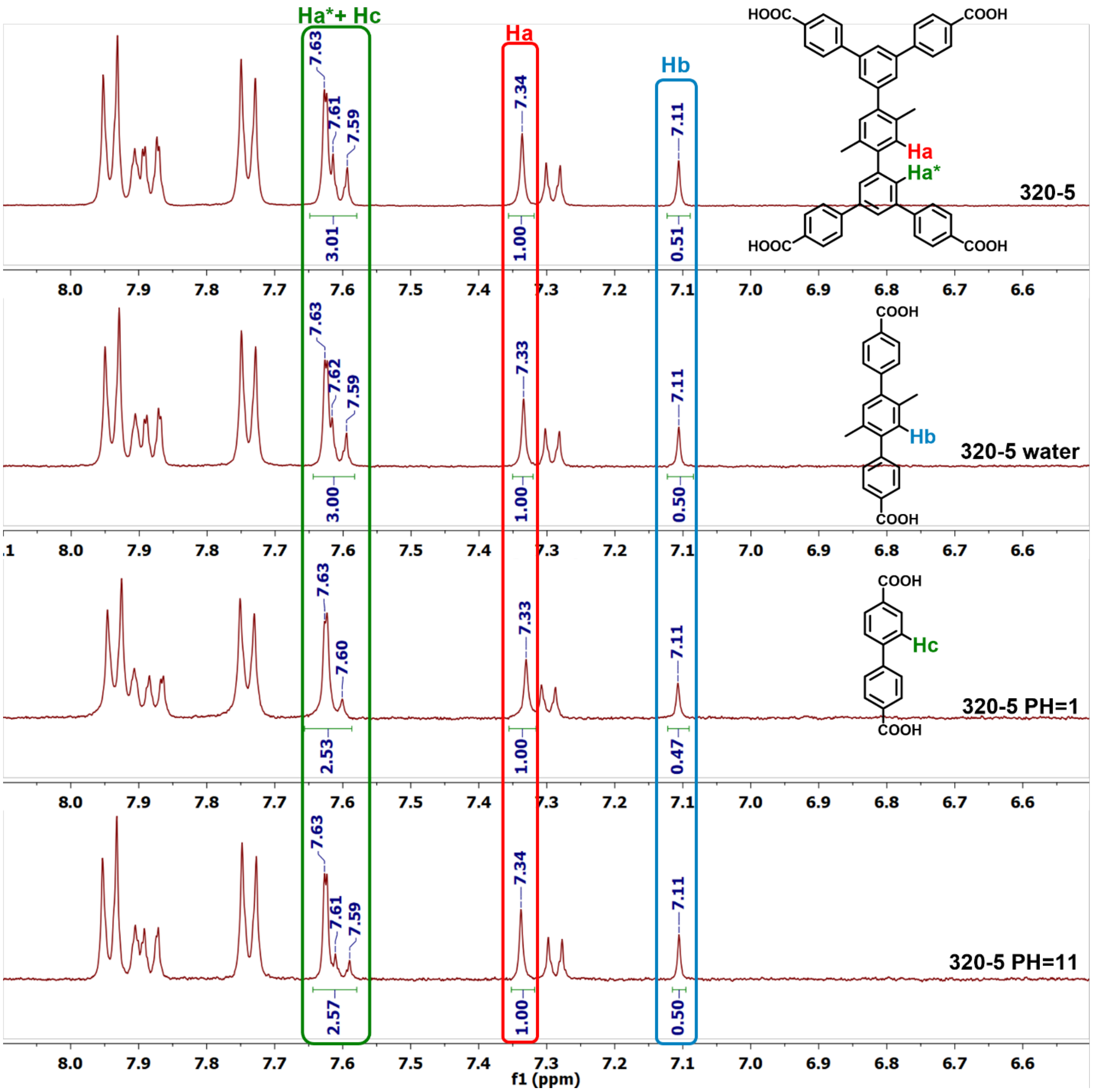

Figure S31. ^{1}H NMR spectrum of digested NPF-320-5 after acid, base, and water treatment for 24 h. (doublet peaks at 7.80 and 7.30 ppm are from benzoic anion)

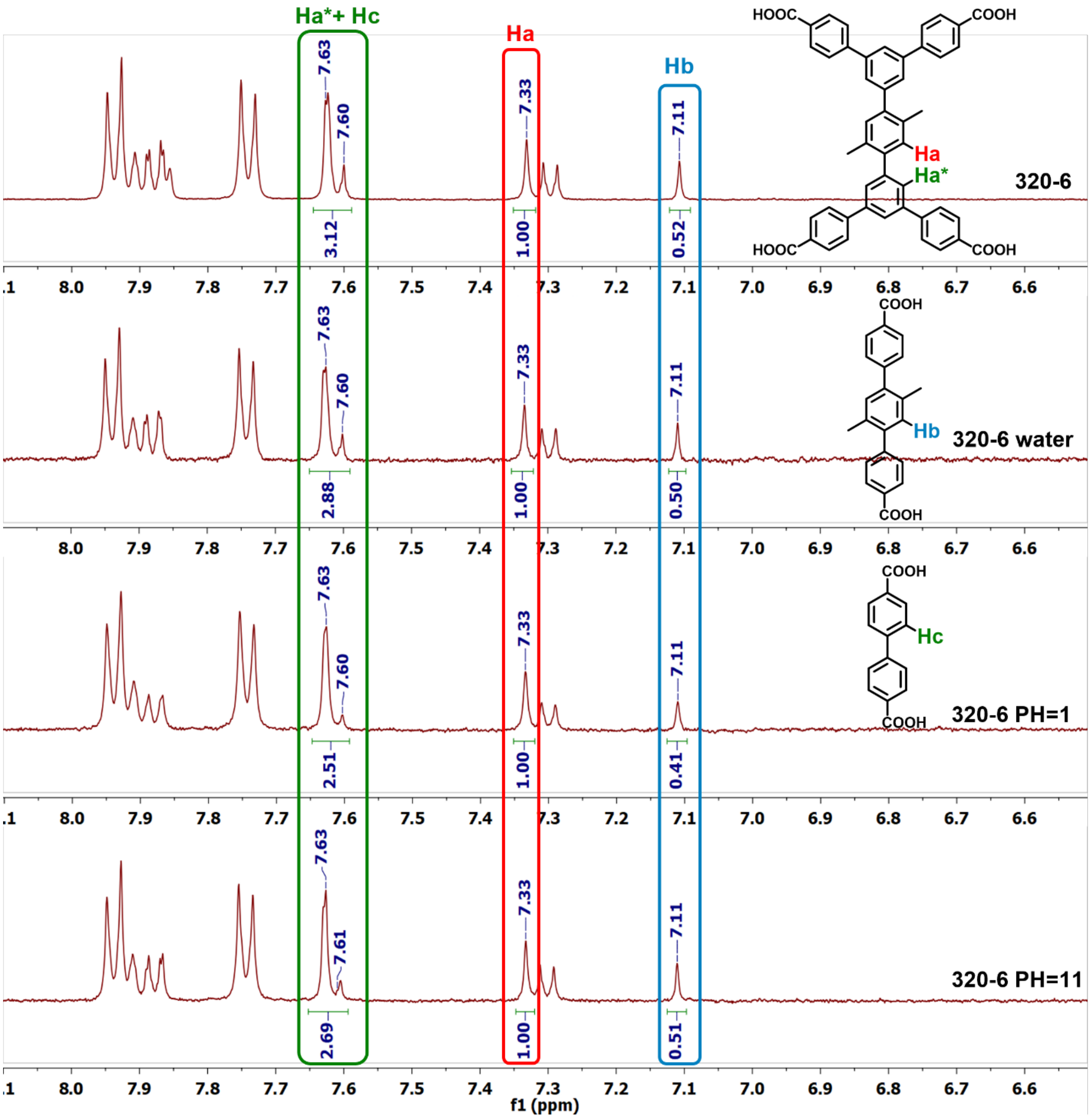

Figure S32. ^1H NMR spectrum of digested NPF-320-6 after acid, base, and water treatment for 24 h. (doublet peaks at 7.30 ppm are from benzoic anion)

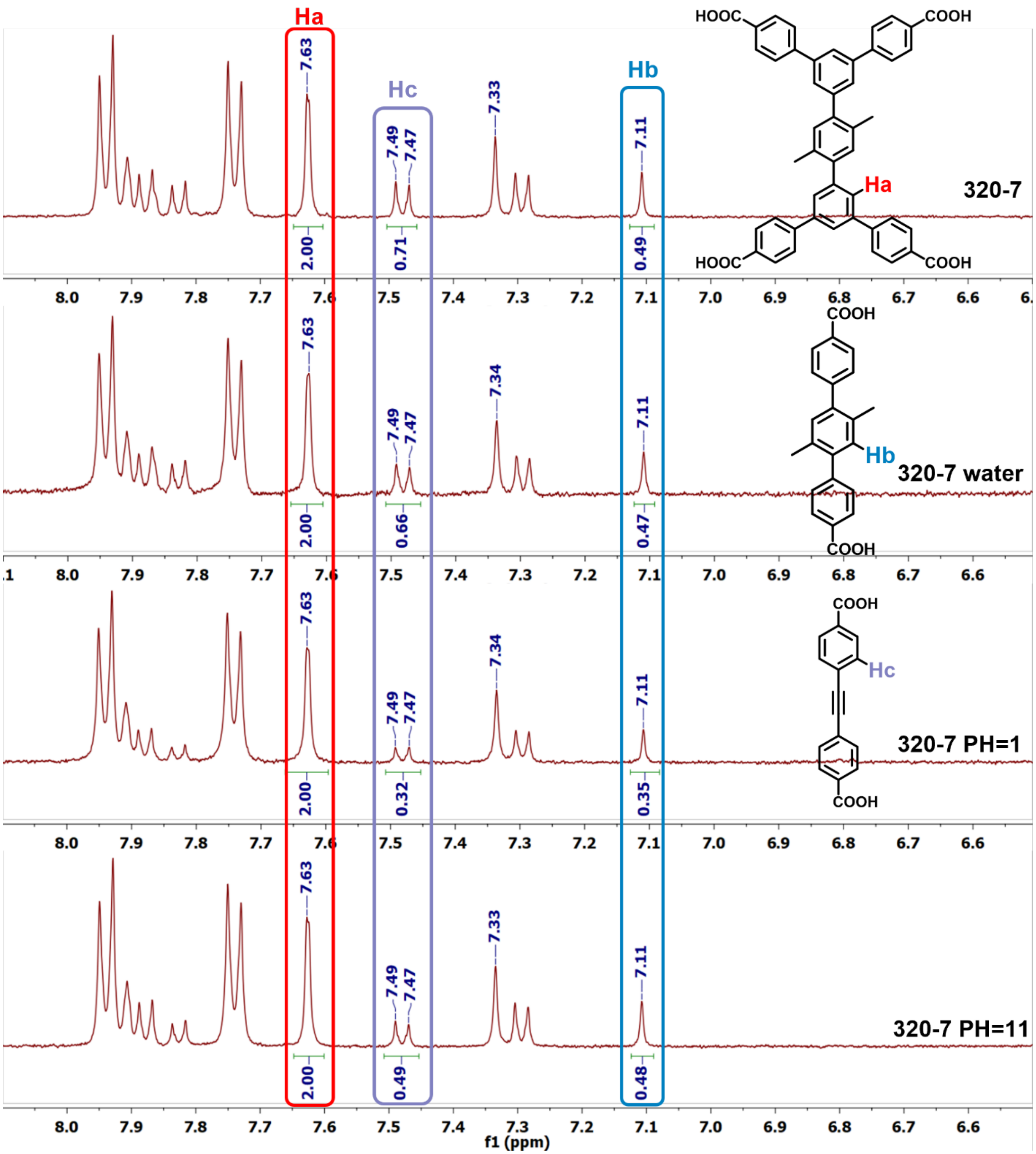

Figure S33. ^{1}H NMR spectrum of digested NPF-320-7 after acid, base, and water treatment for 24 h.

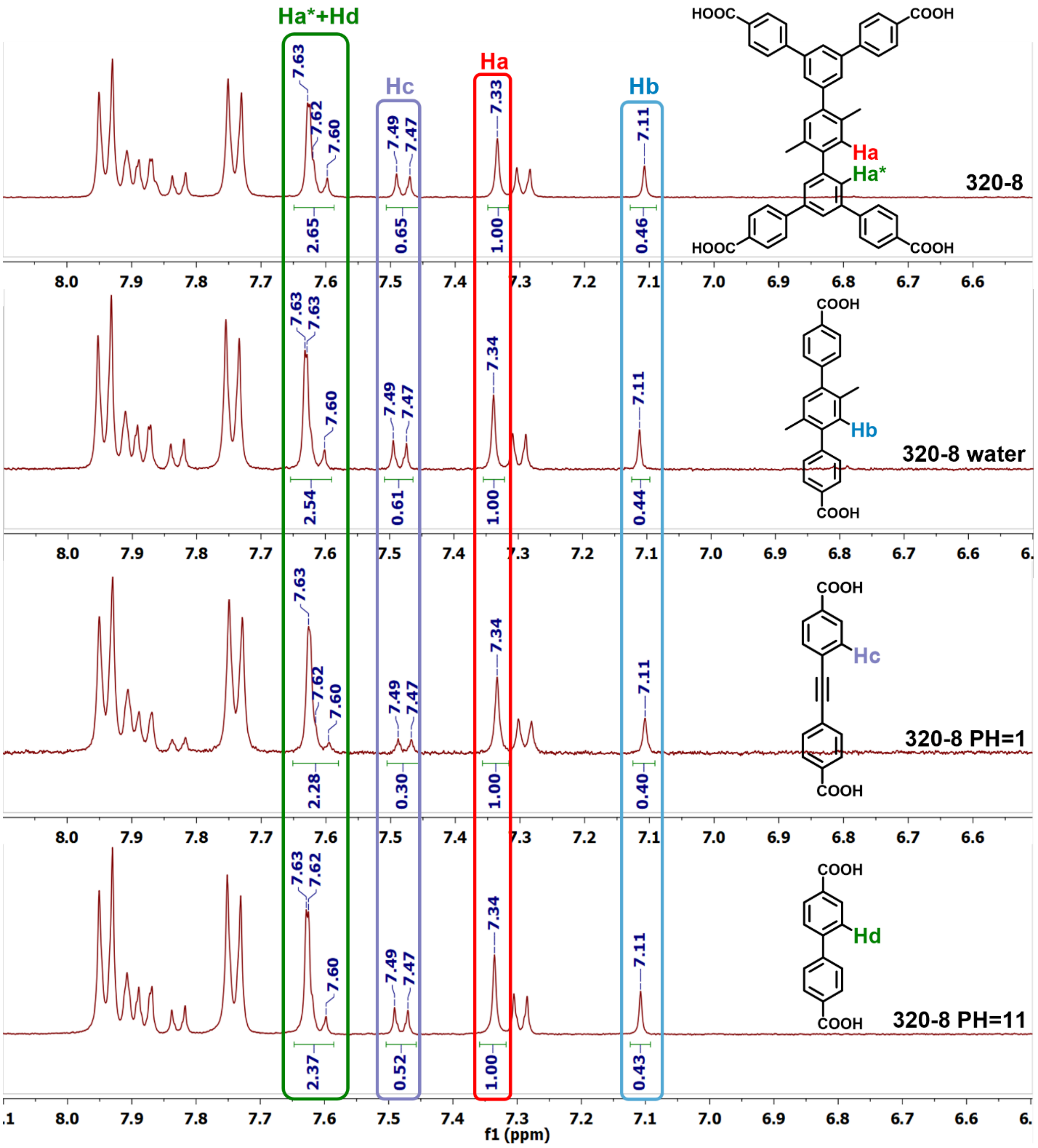

Figure S34. ^{1}H NMR spectrum of digested NPF-320-8 after acid, base, and water treatment for 24 h. (doublet peaks at 7.80 and 7.30 ppm are from benzoic anion)

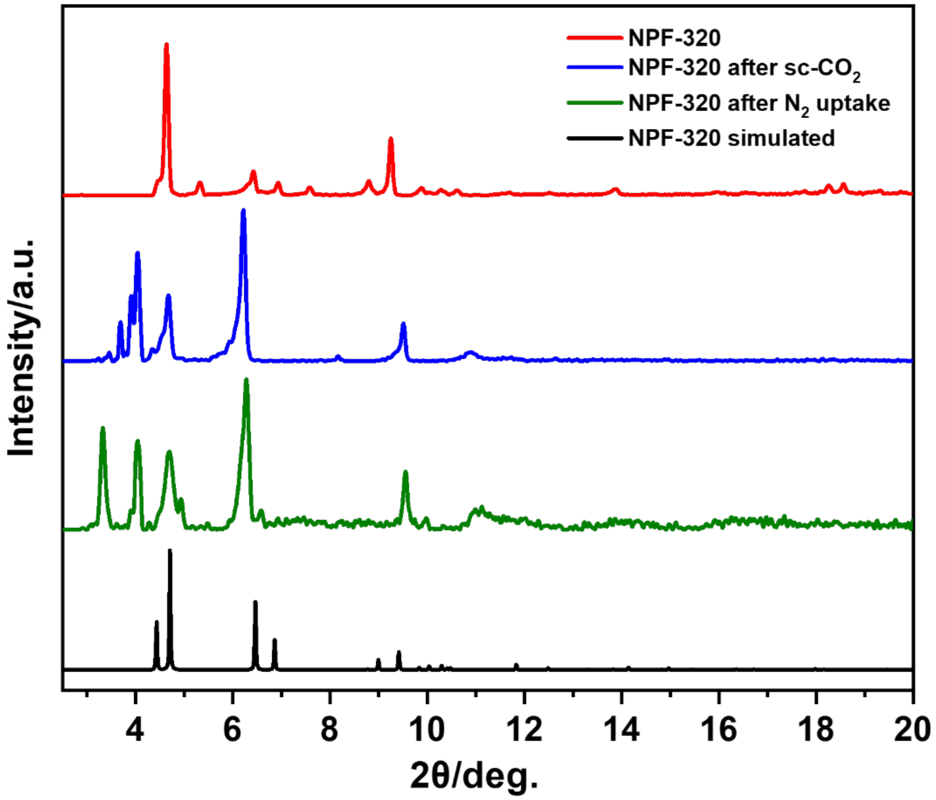

Figure S35. PXRD of NPF-320 after supercritical CO_2 exchange, and N_2 uptake measurement.

S-9 N_2 Uptake and Surface Area

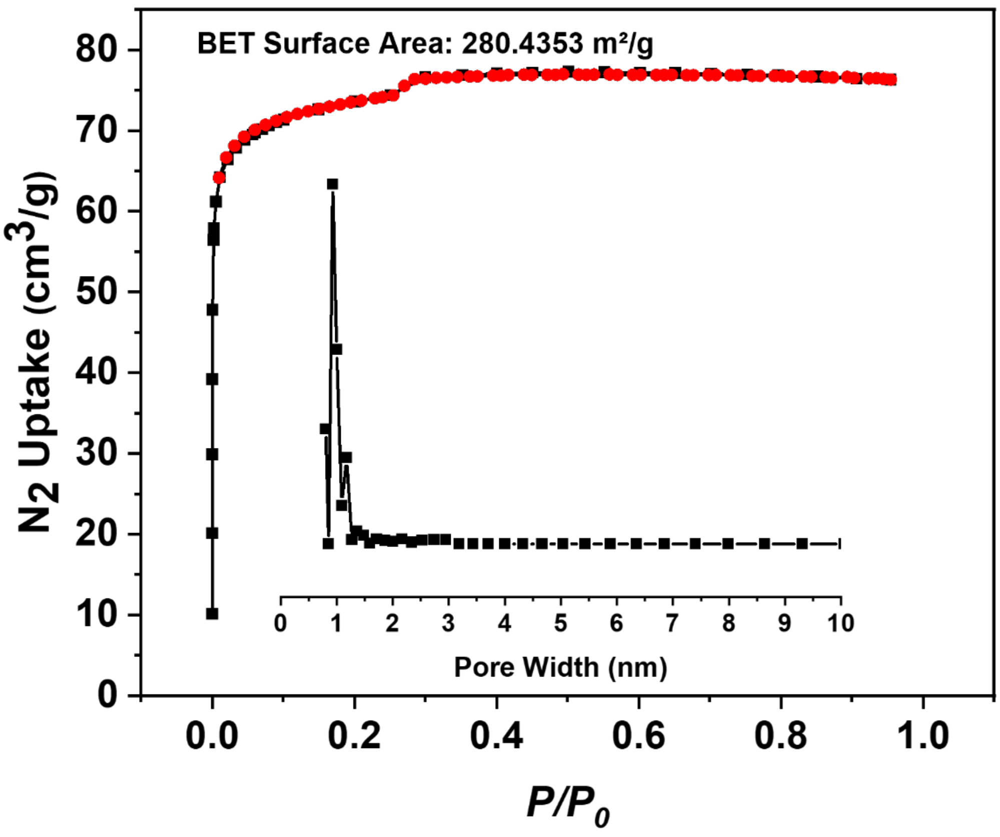

Figure S36. N_2 Adsorption isotherm and DFT pore size distribution of NPF-320.

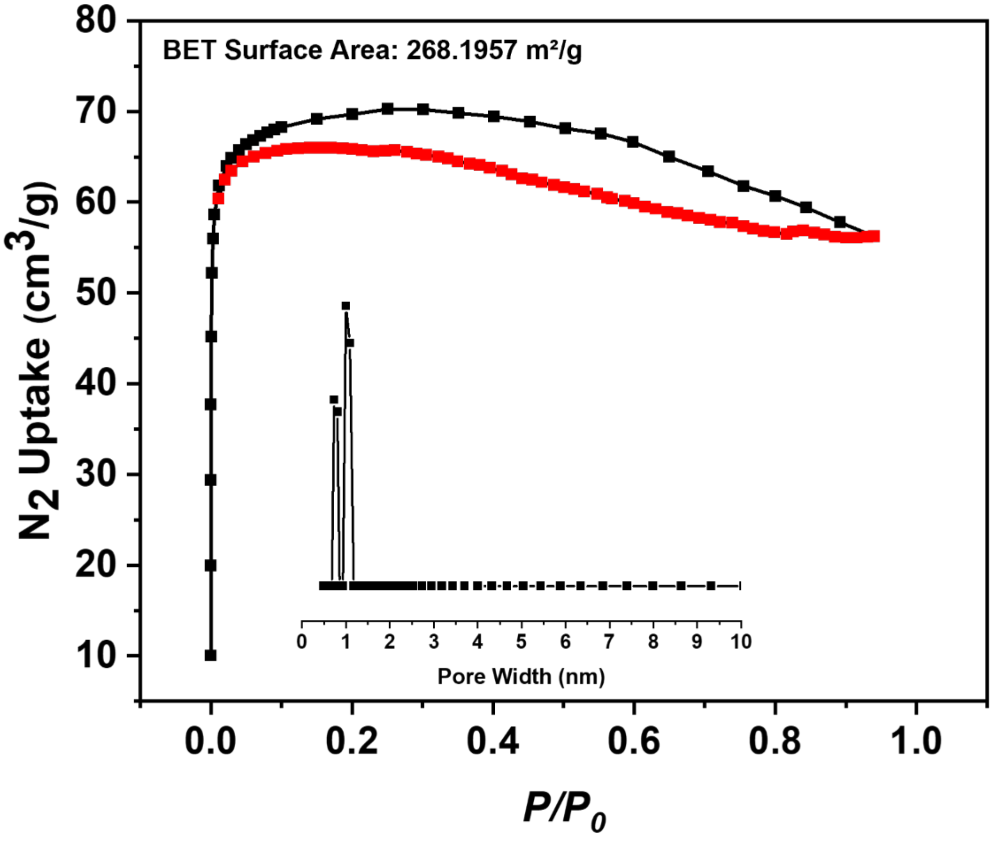

Figure S37. N_2 Adsorption isotherm and DFT pore size distribution of NPF-320-1.

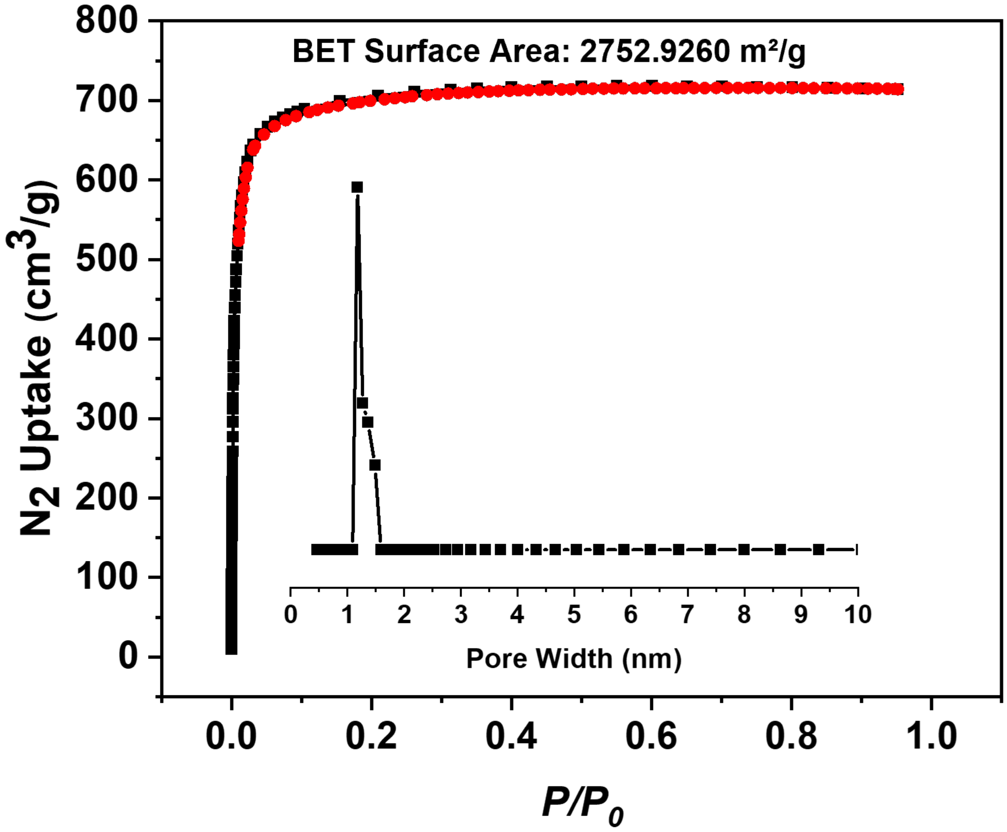

Figure S38. N_2 Adsorption isotherm and DFT pore size distribution of NPF-320-2.

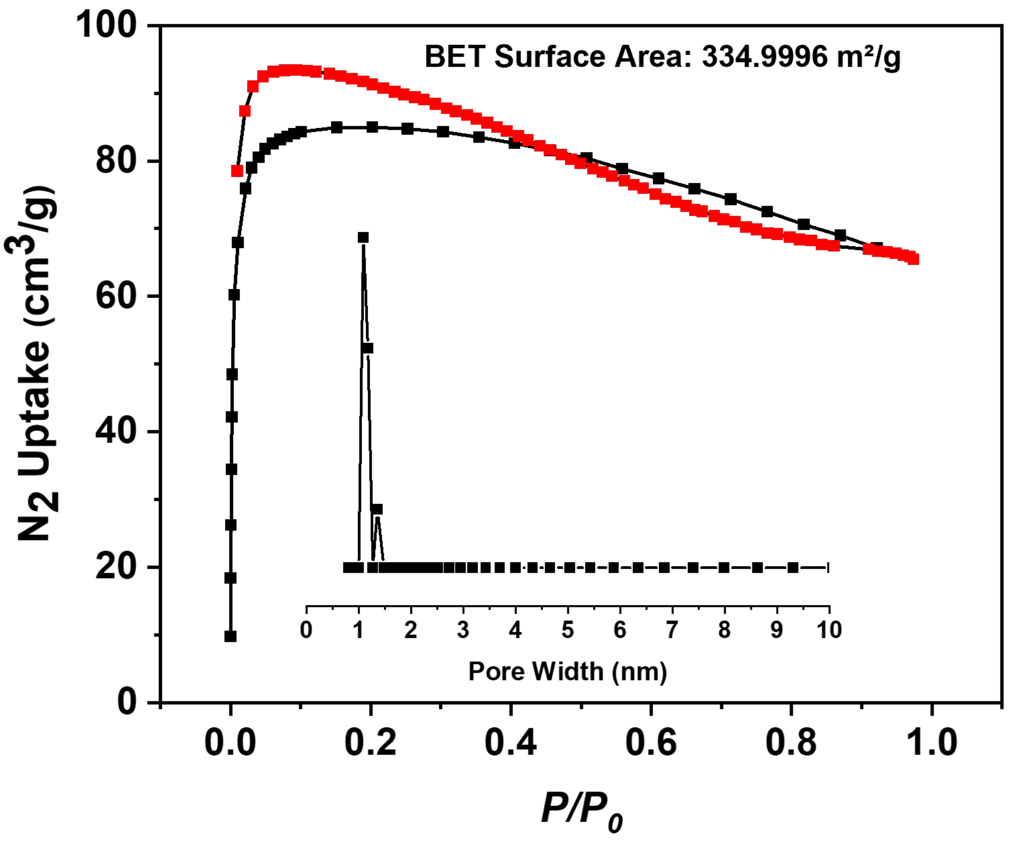

Figure S39. N_2 Adsorption isotherm and DFT pore size distribution of NPF-320-3.

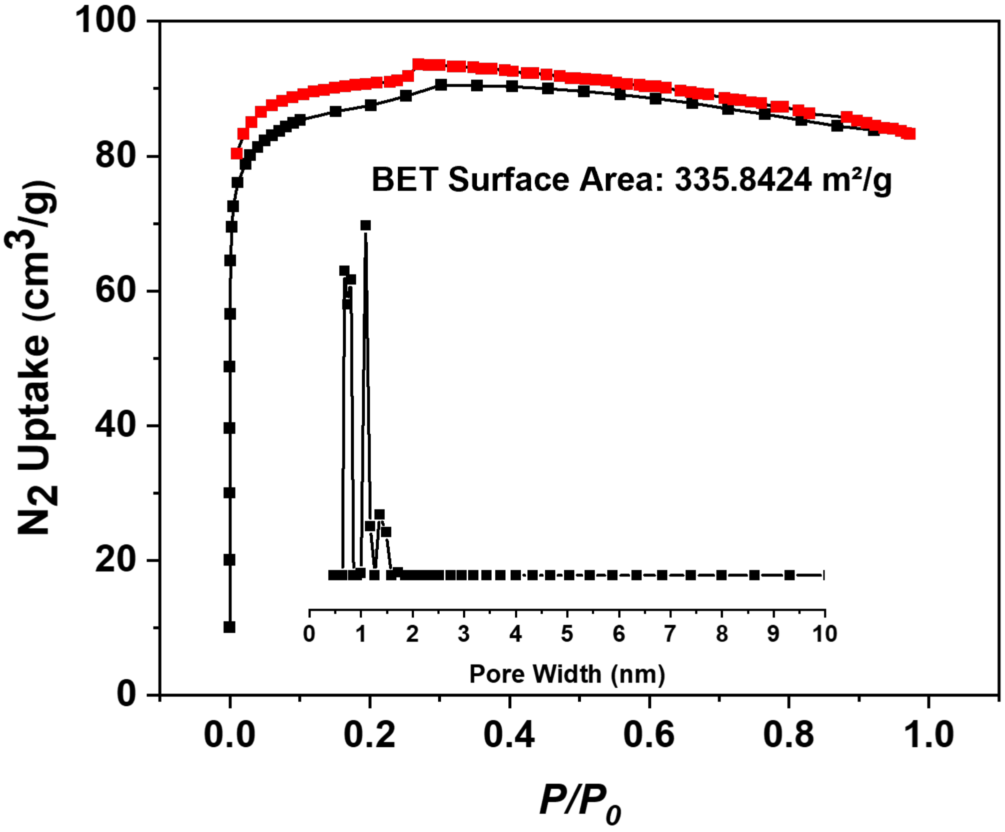

Figure S40. N_2 Adsorption isotherm and DFT pore size distribution of NPF-320-4.

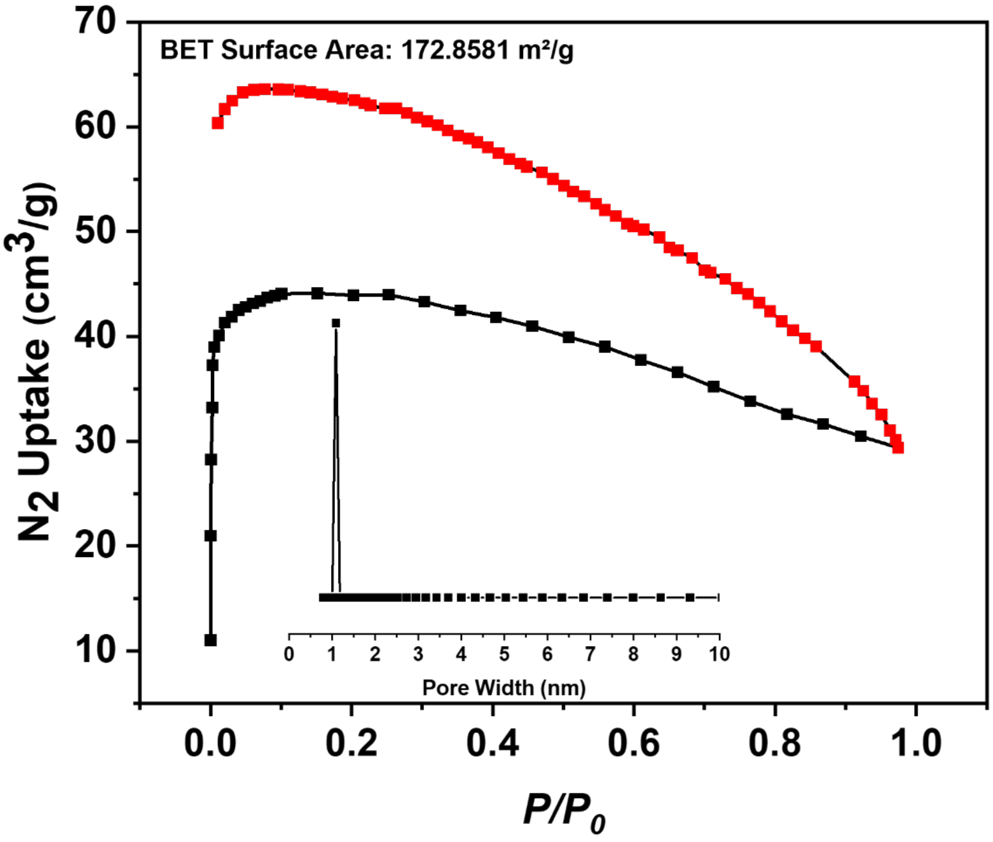

Figure S41. N_2 Adsorption isotherm and DFT pore size distribution of NPF-320-5.

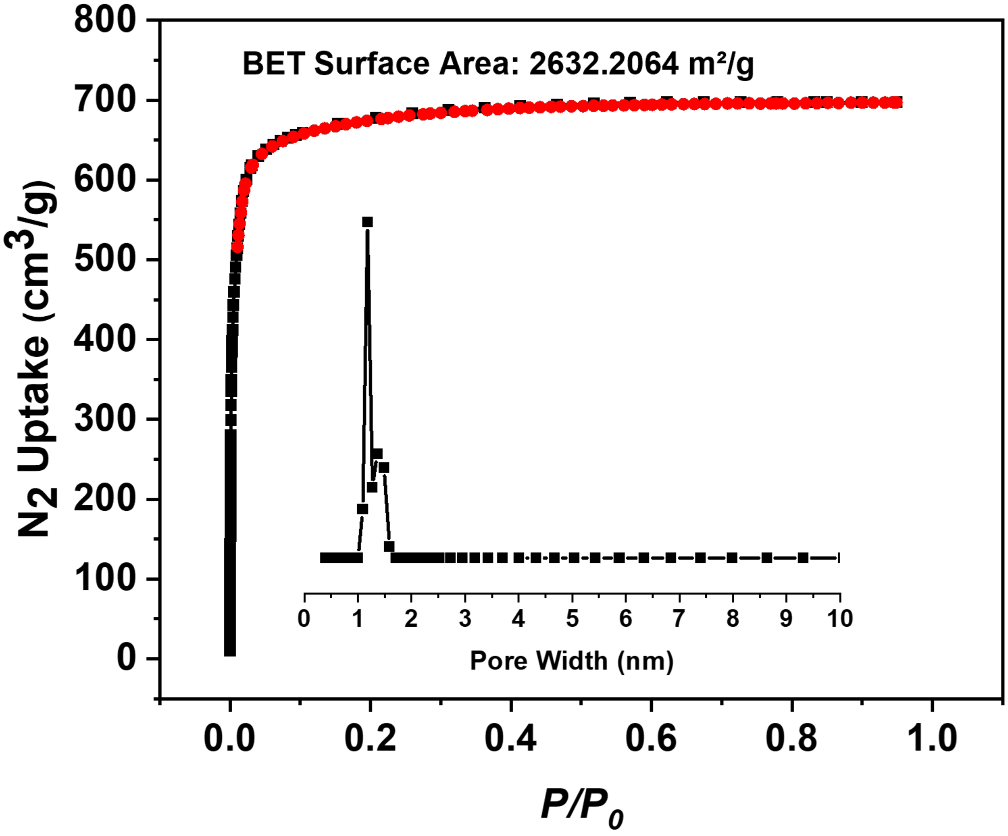

Figure S42. N_2 Adsorption isotherm and DFT pore size distribution of NPF-320-6.

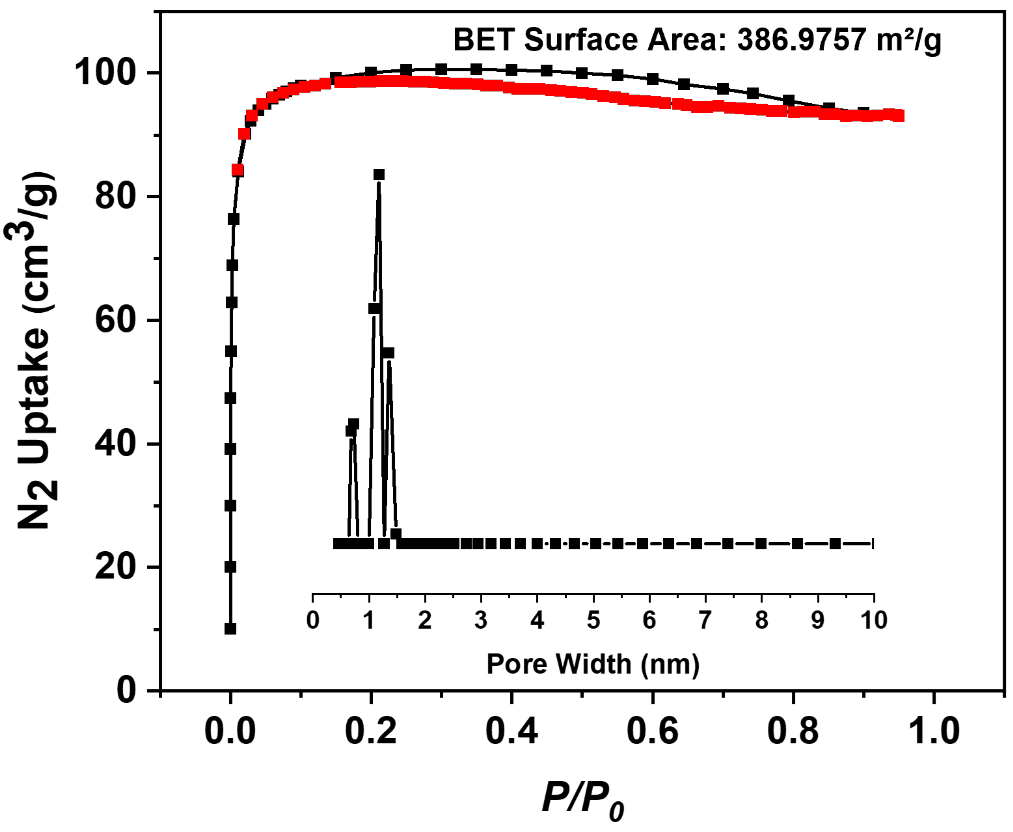

Figure S43. N_2 Adsorption isotherm and DFT pore size distribution of NPF-320-7.

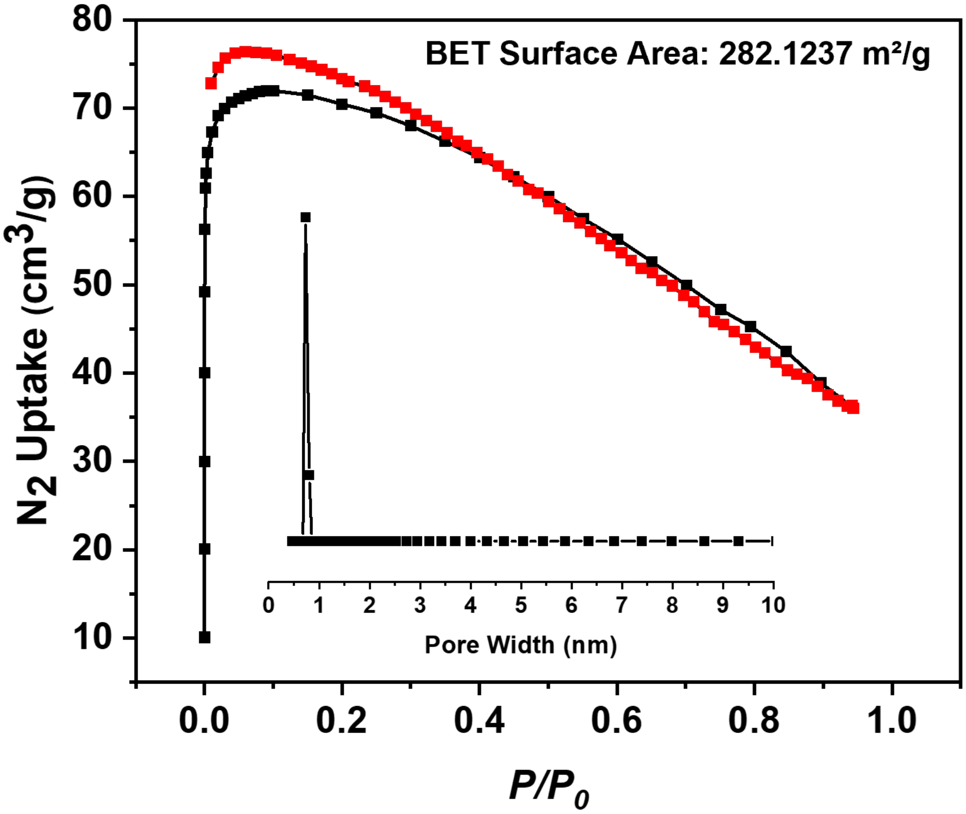

Figure S44. N_2 Adsorption isotherm and DFT pore size distribution of NPF-320-8.

S-10 Energy Transfer Within the MOFs

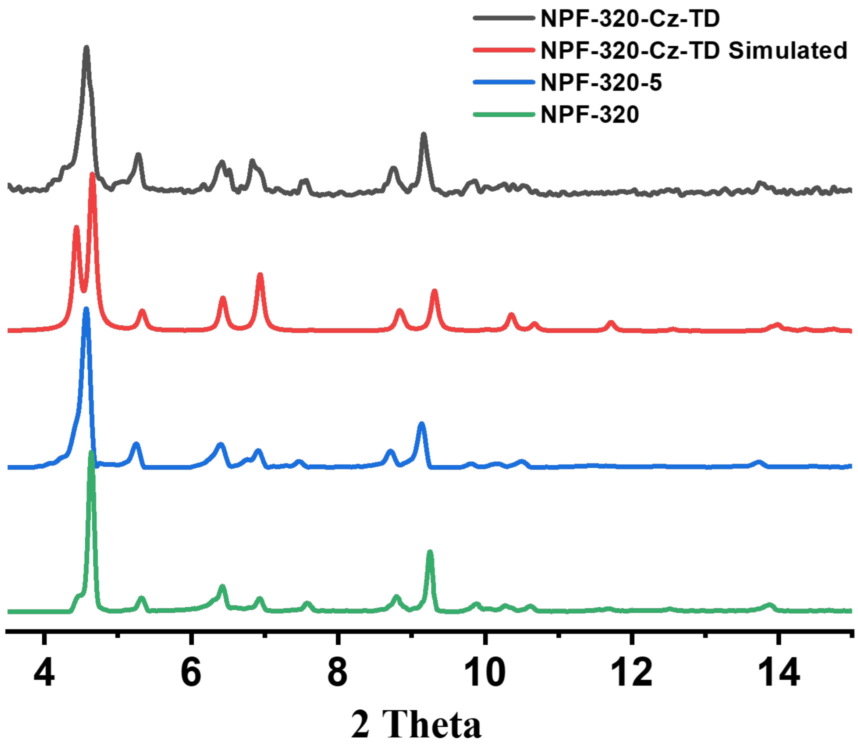

Figure S45. Experimental PXRD patterns of NPF-320-Cz-TD, NPF-320-5 and NPF-320 compared to the simulated PXRD pattern of NPF-320-Cz-TD.

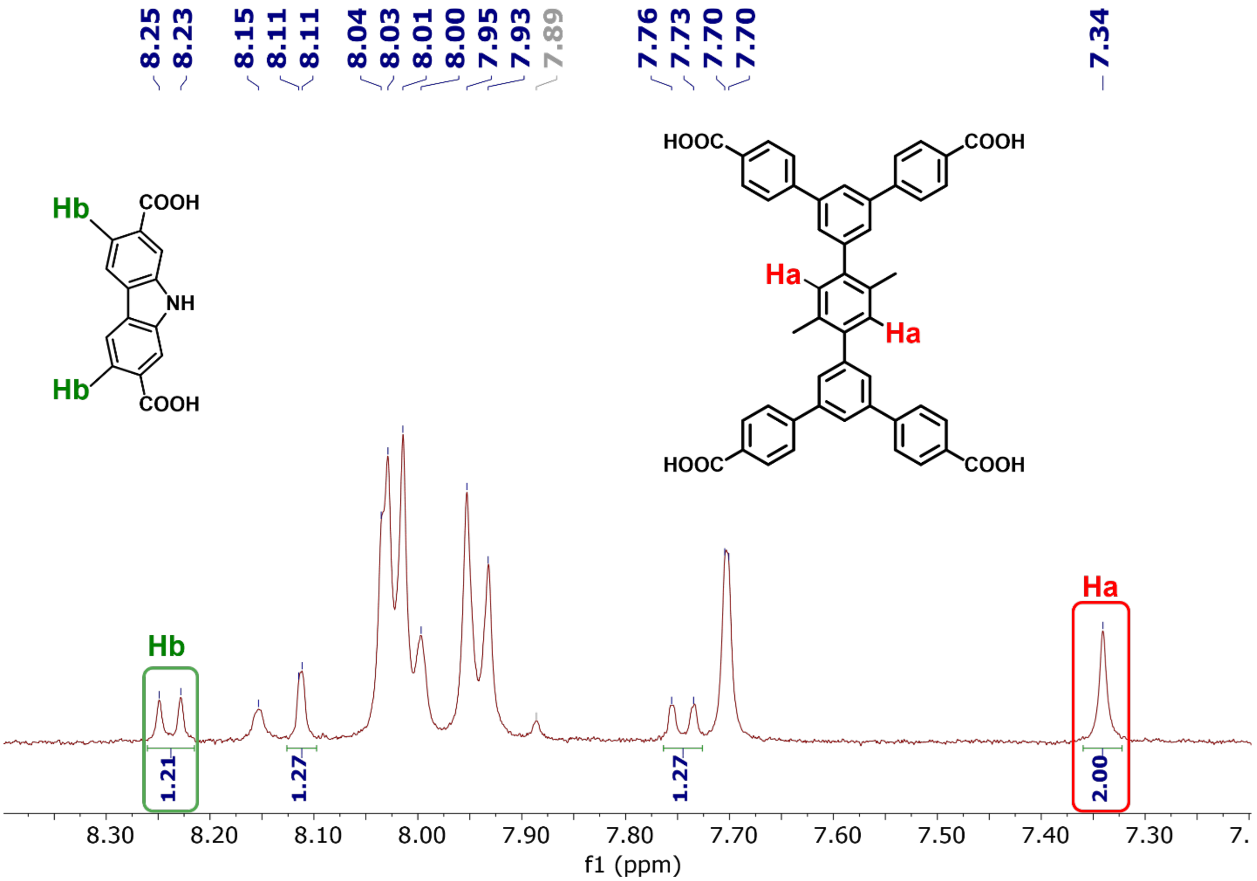

Figure S46. ^{1}H NMR spectrum of digested NPF-320-Cz. **L: Cz** = 2.00: 1.21 (theoretical ratio= 2: 1).

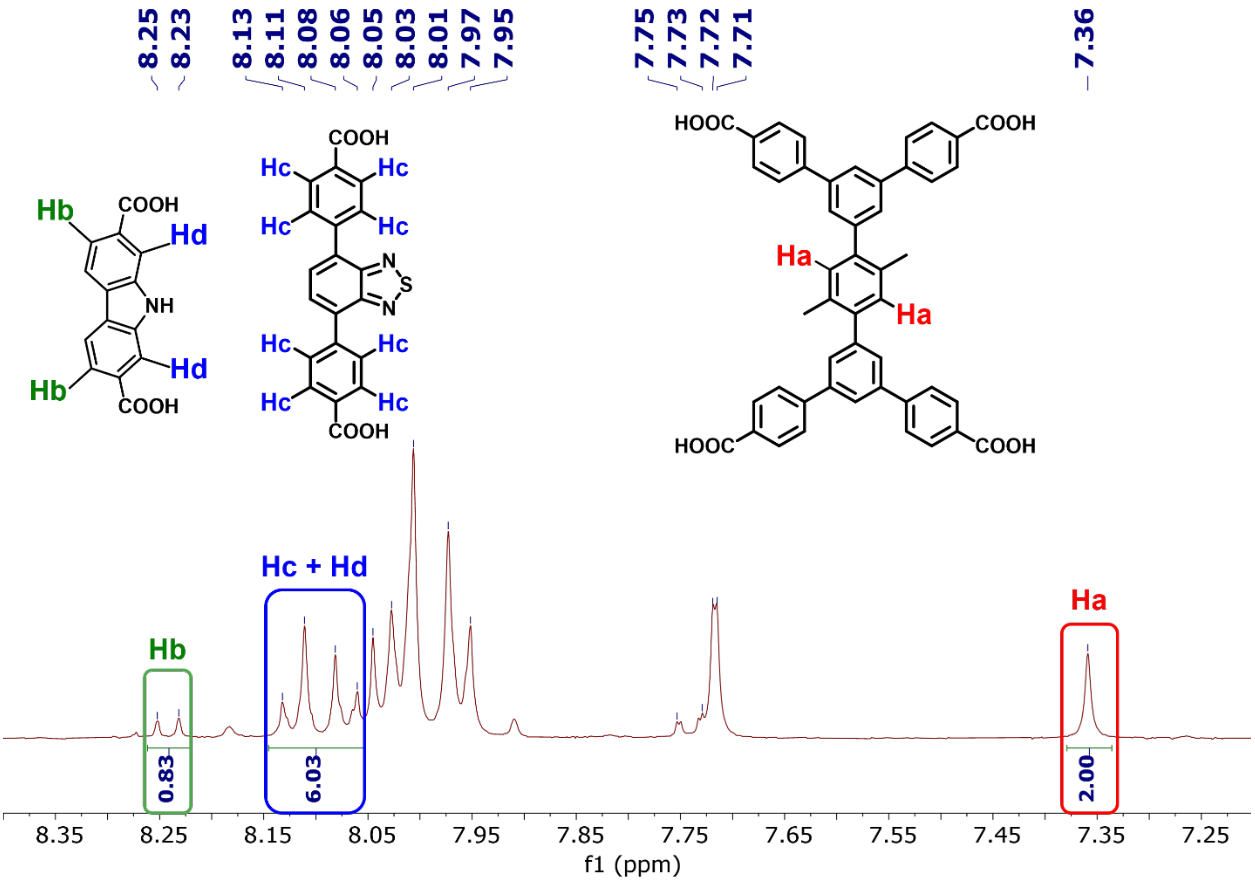

Figure S47. ^{1}H NMR spectrum of digested NPF-320-Cz-TD. **L: Cz: TD** = 2: 0.83: 1.30 (theoretical ratio= 2: 1: 1).

Reference:

1. Mallick, A.; El-Zohry, A. M.; Shekhah, O.; Yin, J.; Jia, J.; Aggarwal, H.; Emwas, A.-H.; Mohammed, O. F.; Eddaoudi, M., *J. Am. Chem. Soc.* **2019,** *141* (18), 7245.
